# Integrative GWAS and Mendelian Randomization Analysis Identifies IREB2 and CD27+ Memory B Cells as Core Drivers of COPD to Lung Cancer Progression

**DOI:** 10.1002/mco2.70473

**Published:** 2025-12-08

**Authors:** Erkang Yi, Qingyang Li, Wenqian Wu, Chengshu Xie, Hairong Wang, Erping Long, Fan Wu, Xuanyi Lu, Yu Liu, Ruiting Sun, Xinqing Lin, Xiaohong Xie, Yumin Zhou, Chengzhi Zhou, Pixin Ran

**Affiliations:** ^1^ State Key Laboratory of Respiratory Disease, National Clinical Research Center for Respiratory Disease, National Center for Respiratory Medicine, Guangzhou Institute of Respiratory Health the First Affiliated Hospital of Guangzhou Medical University Guangzhou Guangdong China; ^2^ Guangzhou National Laboratory Guangzhou Guangdong China; ^3^ State Key Laboratory of Respiratory Health and Multimorbidity Institute of Basic Medical Sciences Chinese Academy of Medical Sciences and Peking Union Medical College Beijing China

**Keywords:** chronic obstructive pulmonary disease, GWAS, IREB2, lung cancer, mendelian randomization

## Abstract

Chronic obstructive pulmonary disease (COPD) associates with increased lung cancer incidence and shares genetic susceptibility, yet its independent causal role and driver mechanisms are poorly understood. We integrated data from the National Health and Nutrition Examination Survey (NHANES) cohort with genome‐wide association studies (GWAS) summary statistics and Mendelian randomization analyses to map genetic correlations and infer causality between COPD phenotypes and lung cancer. Post‐GWAS methods—including transcriptome‐wide association study, colocalization, partitioned heritability via heritability estimation from summary statistics (ρ‐HESS), and cross‐phenotype association (CPASSOC)—identified shared susceptibility loci, highlighting IREB2 and CD27⁺ B cells as potential mediators. Elevated IREB2 expression correlated with accelerated lung‐function decline in COPD but predicted improved prognosis in lung cancer B cells, whereas higher CD27⁺ B cell levels in COPD were associated with protumorigenic activity. Single‐cell transcriptomic analysis and in vitro knockdown experiments confirmed IREB2's role in modulating B‐cell activation and apoptosis pathways within tumors. These results support COPD as an independent lung cancer risk factor and implicate IREB2 and CD27⁺ B cells in COPD‐to‐cancer progression, laying groundwork for early detection and targeted intervention in high‐risk individuals.

## Graphical abstract

This flowchart outlines our integrative pipeline. We linked COPD phenotypes and lung function with lung cancer in NHANES, then applied GWAS, Mendelian Randomization (MR), and colocalization to identify shared loci and driver genes. Candidate genes were refined by eQTL and single‐cell RNA‐seq analyses, and key findings validated in human tissues and B‐cell functional assays.

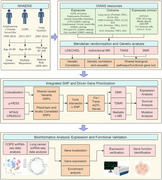

## 1 Introduction

Chronic obstructive pulmonary disease (COPD) is a prevalent age‐related condition strongly associated with smoking. It has become the fourth leading cause of death globally [1]. In China, the overall prevalence of spirometry‐defined COPD is 8.6%, increasing to 13.7% among individuals aged 40 years or older [2]. Moreover, China carries the heaviest global burden of COPD‐related mortality [1]. Lung cancer has emerged as the leading cancer worldwide [3], surpassing breast cancer as the most common malignancy with the highest mortality rate in China [4]. The association between COPD and lung cancer has long been a subject of intense debate [5].

Numerous studies have indicated a significantly higher incidence of lung cancer among individuals with COPD compared with those without the condition, suggesting that COPD itself may be an independent risk factor for lung cancer [6]. However, definitive evidence establishing a causal relationship between the two is lacking. Furthermore, the association between impaired lung function, as measured by forced expiratory volume in 1 s (FEV_1_) and forced vital capacity (FVC), and lung cancer risk remains controversial [7]. Research has additionally shown that each 1% increase in low attenuation areas is independently associated with elevated risks of lung cancer incidence, lung cancer mortality, and overall mortality [8].

Although genome‐wide association studies (GWAS) have identified shared biological alterations between COPD and lung cancer [9, 10], including retention of airborne carcinogens, activation of oncogenes, suppression of tumor suppressor genes, and complex molecular mechanisms of chronic inflammation in the lungs and airways, key driver genes or genetic loci critical for the progression from COPD to lung cancer remain elusive [11, 12]. Despite substantial evidence supporting a strong association between these two diseases, preventive strategies for lung cancer in COPD patients are limited. Early diagnosis remains crucial for improving prognosis.

This study leveraged the National Health and Nutrition Examination Survey (NHANES) database to identify individuals with COPD, emphysema, and chronic bronchitis, assessing lung cancer prevalence within these cohorts. Participants with available lung function data (FEV_1_ and FVC) were included to examine the association between lung function indicators and lung cancer prevalence. Subsequently, linkage disequilibrium score regression (LDSC), high‐definition likelihood (HDL), and Mendelian randomization (MR) analyses were employed to investigate causal relationships among COPD, chronic bronchitis, emphysema, lung function, and lung cancer. Transcriptome‐wide association studies (TWAS), colocalization analyses, sensitivity analysis using cross‐phenotype association test (CPASSOC), and heritability estimation from summary statistics (ρ‐HESS) were conducted to identify shared genetic loci and genes implicated in these conditions. Finally, mediation MR and bioinformatics approaches were utilized to explore the functional roles of key driver genes in the progression from COPD to lung cancer.

## 2 Results

### 2.1 Characteristics of Participants in NHANES and Association of Emphysema/Chronic Bronchitis, COPD, and Lung Function with Lung Cancer

Three NHANES cohorts (1999–2016) were analyzed after applying inclusion criteria. The emphysema/chronic bronchitis cohort (*n* = 29,678) included 117 lung cancer cases, the COPD cohort (2013–2016; *n* = 7160) contained 33 cases, and the lung function cohort (2007–2012; *n* = 8291) had 14 cases. Most cases occurred in individuals aged 40–80 years. Non‐Hispanic White individuals demonstrated the highest lung cancer prevalence. Smoking status showed significant relationship with lung cancer risk (Tables S1–S3 and Figure S1A).

As detailed in Table 1, unadjusted analyses (Model 1) revealed progressively increased lung cancer risk with advancing emphysema severity (*β* = 0.836, *p* < 0.001) and chronic bronchitis severity (*β* = 2.378, *p* < 0.001). Multivariable adjustment (Model 2) for sex, age, race, BMI, and smoking status maintained significant associations (chronic bronchitis: *β* = 0.433, *p* < 0.001; emphysema: *β* = 0.1080, *p* < 0.001). These relationships remained robust in age‐restricted analyses (40–80 years, Model 3–4), with stronger significance thresholds in both unadjusted (chronic bronchitis: *β* **_Model3_** = 1.044, *p* **_Model3_** < 0.001; emphysema: *β* **_Model3_** = 2.555, *p* **_Model3_** < 0.001) and adjusted models (chronic bronchitis: *β* **_Model4_** = 0.827, *p* **_Model4_** < 0.001; emphysema: *β* **_Model4_** = 1.732, *p* **_Model4_** < 0.001).

**TABLE 1:** Weighted Logistic regression analysis results of COPD, emphysema, chronic bronchitis, lung function, and lung cancer.

Independent variables	Model 1^a^ Effect estimate (95% CI)	Model 2^b^ Effect estimate (95% CI)	Model 3^c^ Effect estimate (95% CI)	Model 4^d^ Effect estimate (95% CI)	Model 5^e^ Effect estimate (95% CI)	Model 6^f^ Effect estimate (95% CI)
Chronic bronchitis (yes vs. no)	0.836 (0.827 to 0.844)^***^	0.433 (0.425 to 0.441)^***^	1.044 (1.036 to 1.051)^***^	0.827 (0.819 to 0.834)^***^	2.908 (2.880 to 2.936)^***^	1.780 (1.749 to 1.811)^***^
Emphysema (yes vs. no)	2.378 (2.370 to 2.385)^***^	1.080 (1.072 to 1.087)^***^	2.555 (2.549 to 2.562)^***^	1.732 (1.725 to 1.739)^***^	2.794 (2.772 to 2.817)^***^	2.432 (2.405 to 2.460)^***^
COPD (yes vs. no)	3.241 (3.236 to 3.246)^***^	2.050 (2.045 to 2.055)^***^	2.904 (2.898 to 2.909)^***^	2.084 (2.078 to 2.089)^***^	13.132 (11.223 to 17.966)^***^	13.131 (11.221 to 17.964)^***^
FEV_1_/FVC%	−0.140 (−0.140 to −0.140)^***^	−0.128 (−0.129 to −0.128)^***^	−0.132 (−0.132 to −0.131)^***^	−0.127 (−0.128 to −0.127)^***^	—	—
FEV_1_ (L)	−2.944 (−2.952 to −2.936)^***^	−2.529 (−2.538 to −2.520)^***^	−2.804 (−2.812 to −2.796)^***^	−2.494 (−2.503 to −2.485)^***^	—	—
FVC (L)	−1.670 (−1.676 to −1.664)^***^	−1.259 (−1.266 to −1.251)^***^	−1.436 (−1.442 to −1.430)^***^	−1.285 (−1.293 to −1.277)^***^	—	—

^a^ Model 1: adjusted for none (ages 20–80 years).

^b^ Model 2: adjusted for age (years), sex, race, smoking status, and BMI (ages 20–80 years).

^c^ Model 3: adjusted for none (ages 40–80 years).

^d^ Model 4: adjusted for age (years), sex, race, smoking status, and BMI (ages 40–80 years).

^e^ Model 5: adjusted for none (ages 40–80 years) after PSM.

^f^ Model 6: adjusted for age (years), sex, race, smoking status, and BMI after PSM (ages 40–80 years).

*** *p* < 0.001. All *p* values are two sided.

Abbreviations: CI: confidence interval; COPD: chronic obstructive pulmonary disease; FEV_1_: forced expiratory volume in 1 s; FVC: forced vital capacity; PSM: propensity score matching.

All regression models consistently showed elevated lung cancer risk in patients with physician‐diagnosed COPD versus non‐COPD controls (*β* **_Model1_** = 3.241; *β* **_Model2_** = 2.050; *β* **_Model3_** = 2.904; *β* **_Model4_** = 2.084; *p* **_Mode1‐4_** < 0.001). Moreover, reduced FEV_1_, FVC, and FEV_1_/FVC% predicted higher cancer risk across models (*p* **_Mode1‐4_** < 0.0001), with effect magnitudes persisting regardless of covariate adjustment as shown in Table 1.

Propensity score matching (PSM) balanced demographics (sex/age/race/smoke status) and respiratory conditions (chronic bronchitis/emphysema/COPD) between cases/controls (Tables S4–S7). Post‐PSM analyses confirmed persistent significant associations of chronic bronchitis, emphysema, and COPD with lung cancer, with COPD demonstrating a greater effect (*β* **_Model5_** = 13.132; *p* **_Model5_** < 0.001; *β* **_Model6_** = 13.131; *p* **_Model6_** < 0.001) magnitude compared with prematched estimates (Table 1). Odds ratios (ORs) were calculated for chronic bronchitis, emphysema, and COPD. All showed significant OR elevations across models. COPD demonstrated stronger associations than emphysema/chronic bronchitis. PSM increased effect sizes for chronic bronchitis and emphysema (Table S8). These respiratory disorders collectively exhibited robust lung cancer risk associations, with impaired pulmonary function demonstrating comparable risk elevation.

### 2.2 Genetic Correlation and Causal Effects of Emphysema/Chronic Bronchitis, COPD, and Lung Function with Lung Cancer

**FIGURE 1:**
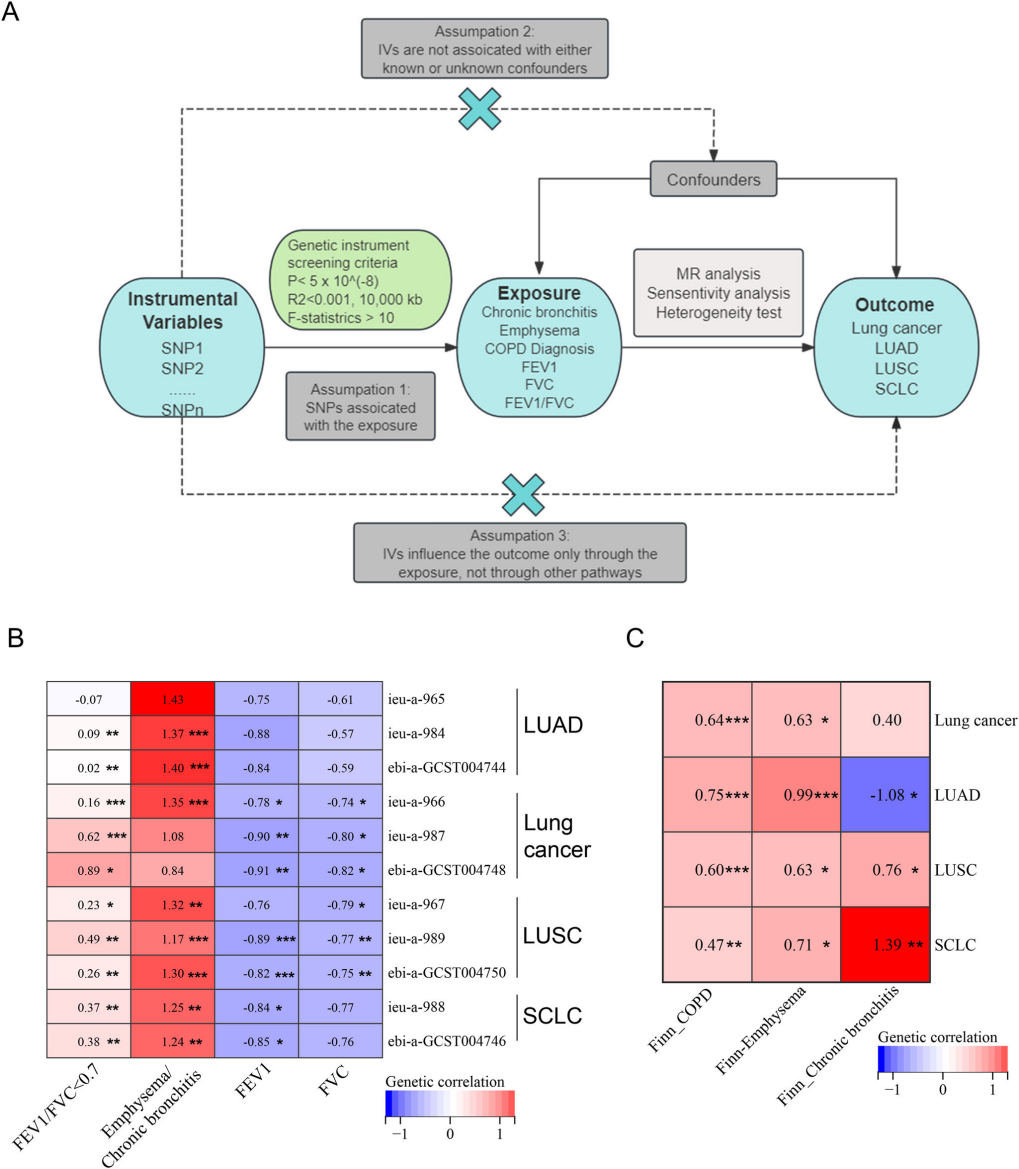
LDSC indicates that COPD, emphysema, chronic bronchitis, and lung function are genetically correlated with lung cancer. (A) MR analyses of COPD, emphysema, chronic bronchitis, lung function, lung cancer, LUAD, LUSC, and SCLC, after conditioning on the effects of SNPs on other cell types. Assumption 1, IVs are associated the exposure; assumption 2, IVs are not affected by confounders; assumption 3, IVs influence outcome solely through exposure, excluding alternative pathways. (B and C) Whole‐genome genetic correlations of COPD, emphysema, chronic bronchitis, and lung function in IEU GWAS (B) and FinnGen GWAS (C) with lung cancer using LDSC. Colors represent the magnitude of the genetic correlation of COPD, emphysema, chronic bronchitis, and lung function with lung cancer (lung cancer, LUAD, LUSC, and SCLC), using LDSC, with red indicating positive genetic correlation and blue indicating negative genetic correlation. Numbers represent the genetic correlation. * (*p* < 0.05), ** (*p* < 0.005), and *** (*p* < 0.001) represent significance. All *p* values are two sided.

First, genetic analyses were performed to reveal shared architecture between chronic respiratory diseases (emphysema/chronic bronchitis/COPD) and lung cancer subtypes [lung adenocarcinoma (LUAD)/lung squamous cell (LUSC)/small cell lung carcinoma (SCLC)], with causal inference through MR (Figure 1A and Data S1). GWAS integration (IEU/GWAS catalog) demonstrated positive genetic correlations between COPD and overall lung cancer, LUSC, and SCLC, though LUAD showed dataset‐specific nonsignificance. Emphysema/chronic bronchitis exhibited parallel positive associations across subtypes, despite null correlations in two cohorts (*ieu‐a‐965* and *GCST004746*). Lung function analysis revealed subtype‐specific patterns: FEV_1_ and FVC showed exclusive negative correlations with overall lung cancer and LUSC (Figure 1B and Data S2).

FinnGen cohort analyses (emphysema/chronic bronchitis/COPD/lung cancer subtypes) replicated the findings of IEU/GWAS catalog, demonstrating significant genetic correlations for emphysema/chronic bronchitis and COPD with lung cancer, LUAD, LUSC, and SCLC (Figure 1C and Data S2). High‐Definition Likelihood (HDL)‐based genetic analyses confirmed these patterns: emphysema/chronic bronchitis and COPD maintained positive correlations with lung cancer/LUAD/LUSC, whereas FEV_1_/FVC exhibited negative correlations. Limited heritability precluded SCLC analysis (Figure S1B,C and Data S3). COPD, emphysema/chronic bronchitis, and impaired pulmonary function demonstrated significant genetic correlations with lung cancer, LUAD, and LUSC, but not with SCLC.

**FIGURE 2:**
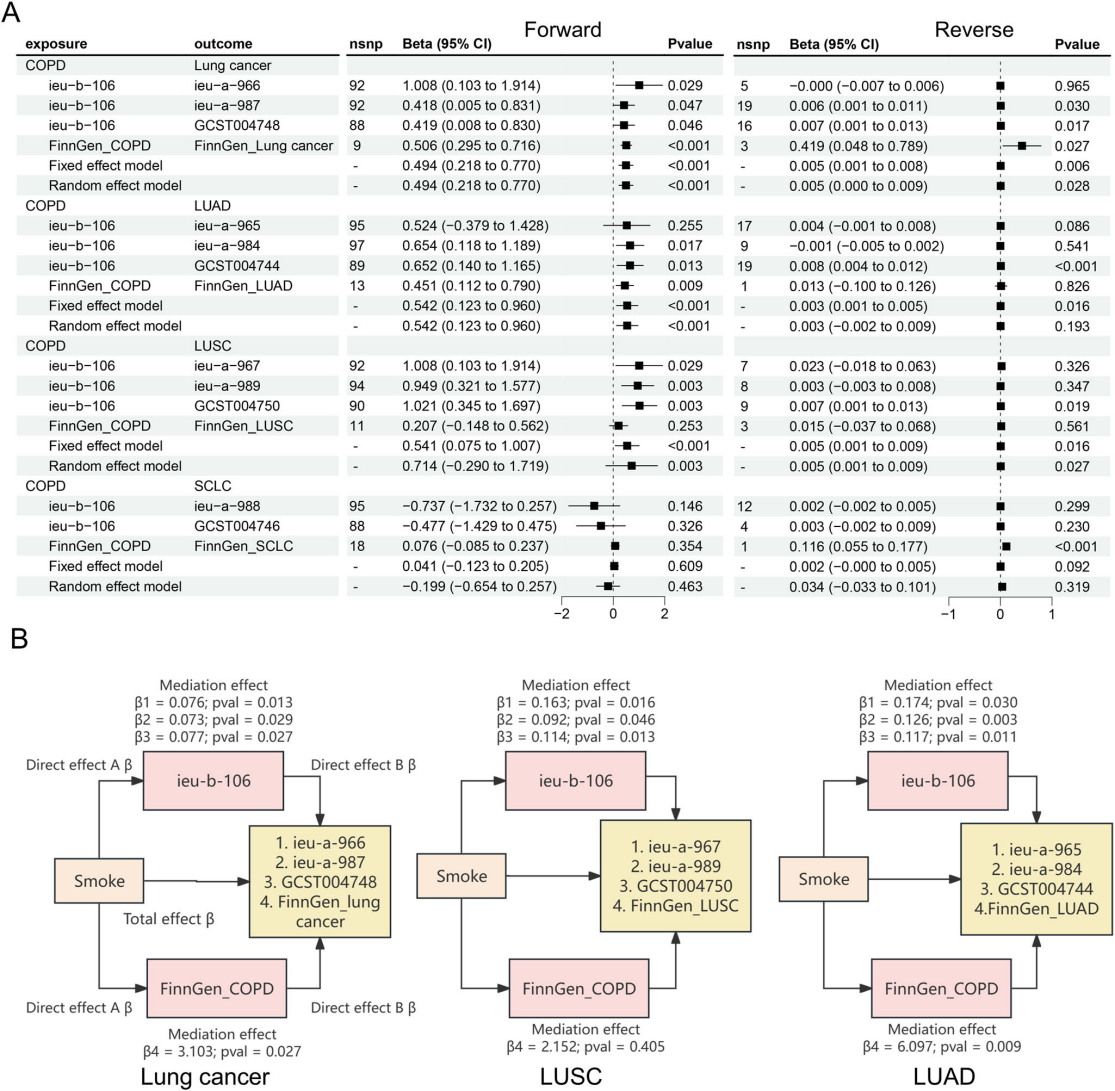
MR results suggest COPD is a driving factor for lung cancer. (A) Forest plot of forward and reverse two‐sample MR analyses between COPD, with lung cancer, LUAD, LUSC, and SCLC after using MR PRESSO after Steiger filtering outlier SNPs. Effect sizes (Beta, 95% CI) are shown as the standard deviation change in lung cancer, LUAD, LUSC, and SCLC per standard deviation increase in COPD. Points on the forest plot represent effect size estimates, while whiskers denote 95% confidence intervals (CIs). Nsnp represents the final number of SNPs included in the analysis. All *p* values are two sided. (B) The diagram illustrates Mendelian randomization assessing COPD‐mediated pathways in smoking‐associated lung carcinogenesis: lung cancer (left), LUSC (middle), and LUAD (right). Rectangular nodes denote exposure (smoke), mediators (COPD), and outcome (lung cancer). Directional arrows represent causal effects with annotated parameters: *β* coefficients for mediation effects (*β*1–*β*4) and total effect *β*, alongside corresponding *p* values.

MR analyses (inverse‐variance weighted, IVW) using GWAS data revealed COPD demonstrated positive causal associations with lung cancer (*β* = 0.419–1.008), LUAD (*β* = 0.451–0.654), and LUSC (*β* = 0.207–1.021), but not SCLC. FEV_1_ showed negative causality with LUSC (*β* = −0.328 to −0.251), while FVC exhibited no significant associations. Reverse MR indicated weaker bidirectional COPD–lung cancer links (Figure 2A and Data S4 and S5). Colocalization analyses identified shared loci between COPD/emphysema and lung cancer (*SNP.PP.H4>0.8*) in FinnGen datasets (Data S6).

It is well established that smoking is a primary risk factor for both COPD and lung cancer, smoking demonstrated dual causality as a risk factor for both COPD and lung cancer in two‐sample MR analyses (Data S7). Mediation MR quantified COPD's partial mediation of smoking‐induced lung cancer risk, with stronger mediation effects observed for LUAD and LUSC despite cohort heterogeneity (Figure 2B and Data S8). Sensitivity analyses excluding COPD‐mediated pathways revealed attenuated smoking‐lung cancer associations (Figure S2A). The above results indicated COPD significantly mediates smoking‐induced lung carcinogenesis.

### 2.3 TWAS Analysis and SMR Analysis for COPD and Lung Cancer

Transcriptome‐wide analyses (TWAS) revealed progressive genetic overlap among chronic respiratory diseases and lung cancer, with COPD showing the strongest associations (45 tissue/50 blood loci; Data S9), exceeding emphysema/chronic bronchitis (14/12 loci; Data S10). The FinnGen cohort corroborated these findings (COPD: 91/102 loci; emphysema: 56/51; chronic bronchitis: 28/35; Data S11–S13).

**FIGURE 3:**
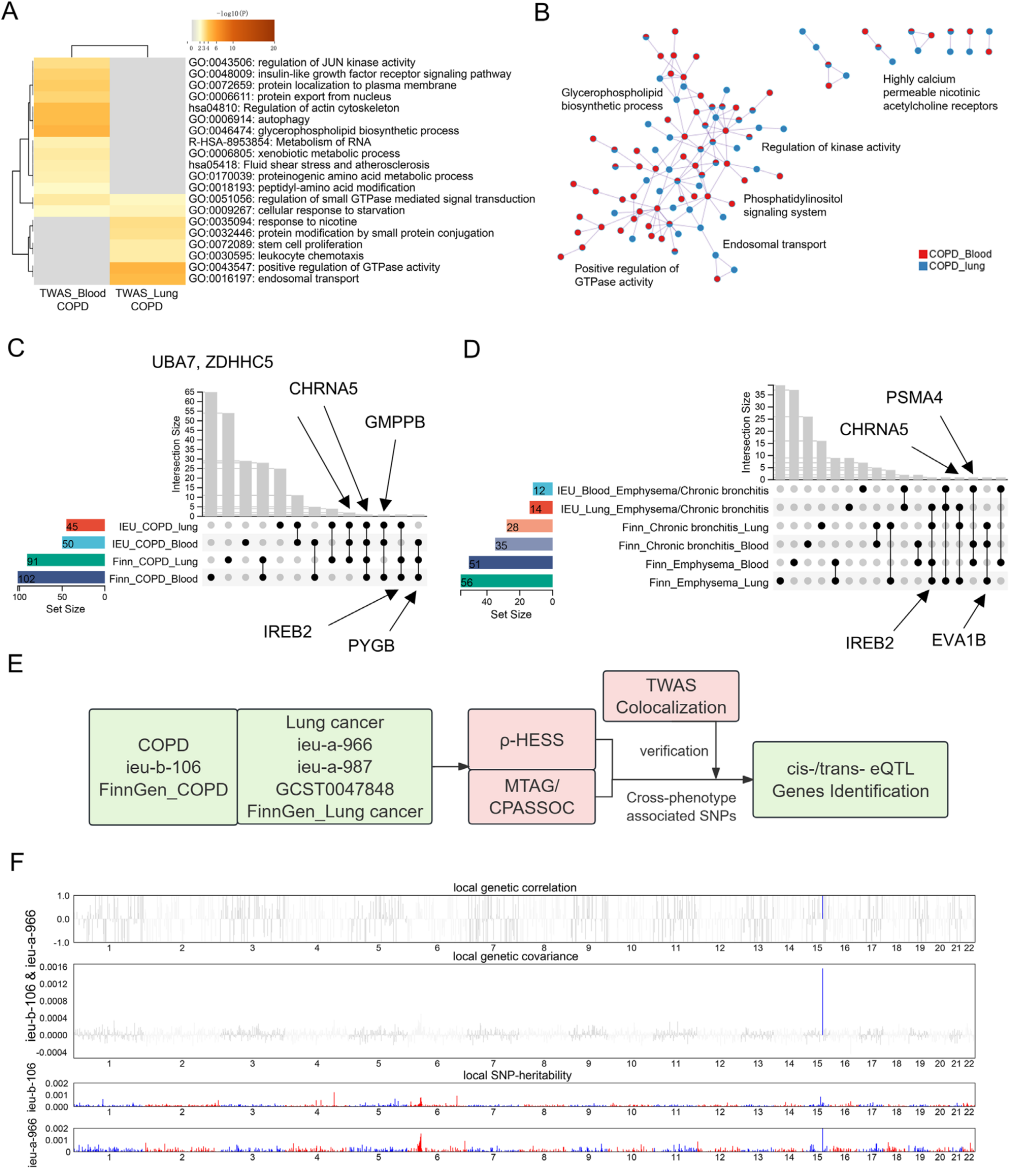
Overlapping gene loci and functions of COPD and lung cancer in lung tissue and peripheral blood. (A and B) Enrichment (A) and PPI network (B) analysis of overlapping genes from TWAS of COPD and lung cancer in lung tissue and peripheral blood using Metascape. (C and D) Upset plot showing the overlapping genes from TWAS of COPD (C) and emphysema/chronic bronchitis (D) with lung cancer in lung tissue and peripheral blood. (E) This workflow begins with COPD (*ieu‐b‐106*, *FinnGen_COPD*) and lung cancer (*ieu‐a‐966, ieu‐a‐987, GCST004784, FinnGen_Lung_cancer*) GWAS datasets. Cross‐phenotype associations are established through p‐HESS analysis and verified by MTAG/CPASSOC integration. Subsequent trans‐ethnic TWAS and colocalization analyses identify pleiotropic SNPs. cis‐/trans‐eQTL mapping (highlighted in light green) and secondary colocalization/TWAS validation (light red) prioritize causal genes underlying disease comorbidity. Arrows indicate analytical progression from raw data to gene prioritization. (F) Genome‐wide local heritability and genetic correlation between *ieu‐b‐106* (COPD) and *ieu‐a‐966* (lung cancer). The top two panels display the local SNP heritability estimates for COPD and lung cancer, where statistically significant positive correlations are indicated in blue and statistically significant negative correlations are indicated in red. The third panel presents local genetic covariance, with positive covariance shown in blue and negative covariance shown in red.

Pathway analysis showed COPD–lung cancer overlaps in small GTPase signaling and starvation response, with blood‐specific enrichment for JUN kinase, IGF1R signaling, and autophagy, and tissue‐specific enrichment for endosomal transport and leukocyte chemotaxis (Figure 3A). PPI networks implicated kinase regulation, endosomal transport, Phosphatidylinositol signaling, calcium‐permeable nicotinic receptors, and glycerophospholipid biosynthesis (Figure 3B).

Emphysema/chronic bronchitis exhibited fewer overlaps. Emphysema shared the peptidyl‐amino acid modification pathway across tissues, with FCER1 signaling in blood and tube morphogenesis in lung tissue; related PPIs mapped to FCER1 signaling and calcium‐permeable nicotinic receptors (Figure S2B). Chronic bronchitis showed minimal overlap, enriched for GTPase regulation in blood and ferroptosis/chemotaxis in lung tissue, with PPIs implicating chromosome segregation, ferroptosis, and chemotaxis (Figure S2C).

Intersection analysis identified core COPD–lung cancer genes (*UBA7, ZDHH5, GMPPB, IREB2, PYGB*; Figure 3C and Data S14), with emphysema‐specific *IREB2* (lung) and *PSMA4* (blood; Figure 3D). Overall, genetic correlation strength followed COPD > emphysema > chronic bronchitis, consistent with pathway enrichment patterns.

SMR analysis of eQTL/pQTL data identified 1552/1308 COPD eQTL genes and 231/151 pQTL proteins. Lung cancer datasets (*GCST004748, ieu‐a‐966, ieu‐a‐987, Finn Gen*) revealed ∼1000 eQTL genes and ∼120 pQTL proteins each (Figure S3A and Data S15 and S16). *AGPHD1*, *TMX2*, *ITGAL*, and *CCNA2* emerged as cross‐disease signatures (Figure S3B), while *UBE2L6*, *TLR4*, and *PRSS53* dominated pQTL findings (Figure S3C). Enrichment showed convergence on DNA damage response, cell cycle, TP53‐mediated transcription, MAPK regulation, and pathways of migration, adhesion, and proliferation (Figure S4A,B). Collectively, TWAS with SMR validation demonstrated shared pathobiological foundations between COPD and lung cancer.

### 2.4 Identification of Core Shared Genetic Loci and Genes Between COPD and Lung Cancer

Colocalization analysis (*PP.H4>0.8*) identified disease‐specific SNP signatures across respiratory disorders and lung cancer subtypes. COPD shared rs58034696 (*ieu‐b‐106*) and rs3840839/rs61496709 (*FinnGen_COPD*) with lung cancer, while emphysema/chronic bronchitis‐specific loci were detailed in Data S17.

PheWAS of colocalized SNPs revealed multiphenotype associations across COPD, emphysema, chronic bronchitis, lung function, and lung cancer. Core SNPs (rs4886572/rs12441354/rs61496709) demonstrated trans‐ancestry effects, while six loci showed no significant associations (Data S18). Cis‐eQTL mapping linked eight regulated genes to core SNPs, notably *CHRNA3, IREB2*, and *PSMA4* with tri‐SNP coregulation (Data S19).

Cross‐trait meta‐analyses and ρ‐HESS identified risk SNPs for COPD–lung cancer overlap (Figure 3E), primarily on chromosomes 4, 6, and 15 (Data S20). PheWAS confirmed chromosome 15 SNPs as jointly associated with respiratory traits and lung cancer (Data S21), regulating *IREB2, CHRNA3/5, HYKK*, and *PSMA4*. Variants on chr4 and chr6 mapped to *HHIP/NPNT* (lung development) and MHC pathways (Data S22).

Genome‐wide ρ‐HESS analyses revealed significant COPD–lung cancer genetic correlations, localized to chr6p21 and chr15q25 (Figure 3F and Figure S5A‐C and Data S23). The chr15:78.5–80.9 Mb region remained significant after correction (Data S24 and S25). Cross‐validated SNPs rs16969968/rs12914385, cis‐regulatory hubs for COPD–lung cancer pathogenesis, colocalized with ρ‐HESS‐identified regions, mechanistically linking dysregulation of *PSMA4, IREB2, CHRNA3*, and *CHRNA5* to disease etiology (Data S26).

### 2.5 IREB2 Identified as a Key Genetic Factor in the Progression from COPD to Lung Cancer

**FIGURE 4:**
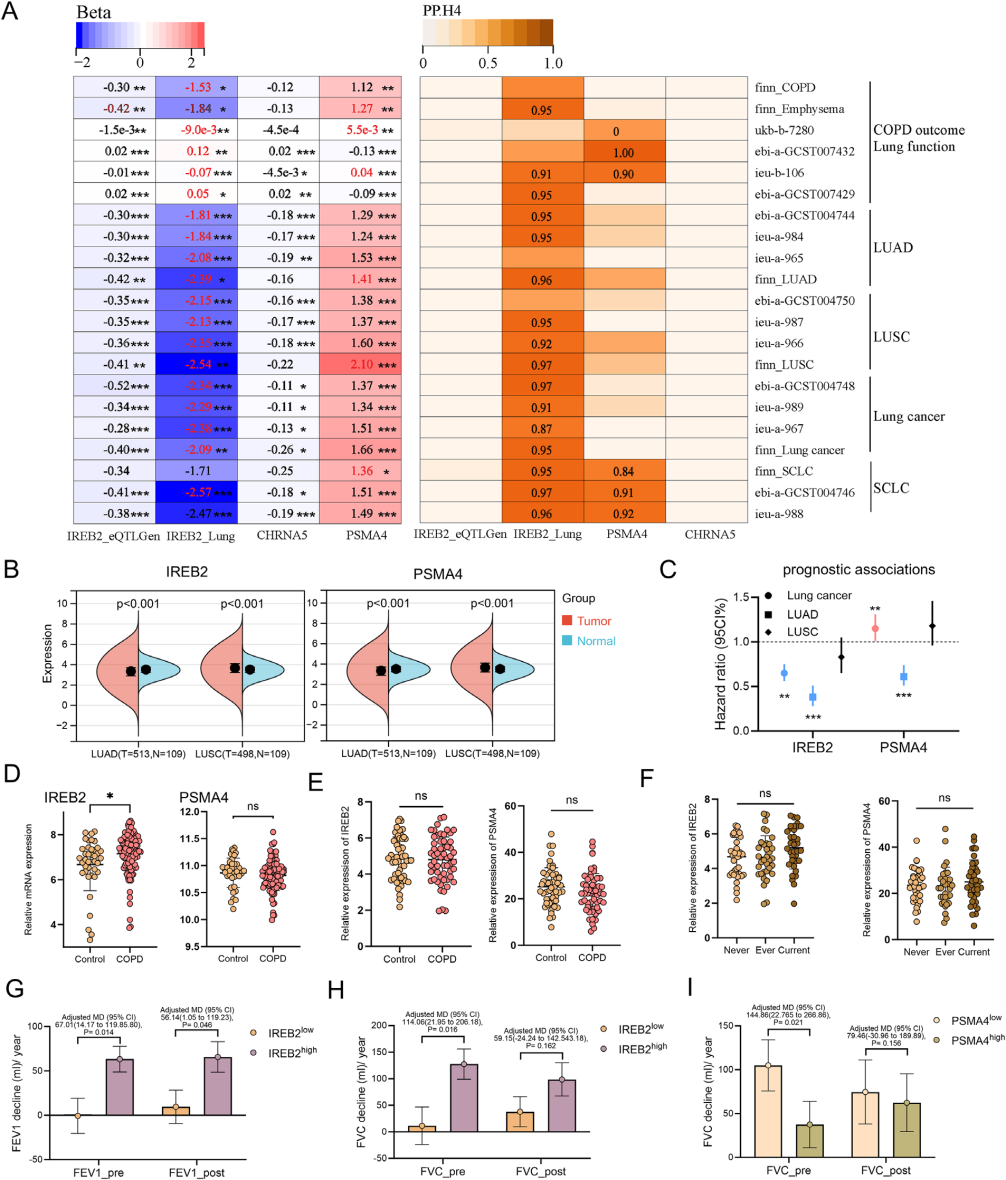
Overlapping gene loci and functions of COPD and lung cancer in lung tissue and peripheral blood. SMR (A) and colocalization (B) results of *IREB2, PSMA4*, and *CHRNA5* in lung tissue and peripheral blood for COPD, emphysema, chronic bronchitis, lung function, lung cancer, LUAD, LUSC, and SCLC. Colors represent the effects of IREB2, PSMA4, and CHRNA5 on these conditions, with red indicating positive genetic correlation and blue indicating negative genetic correlation. Numbers represent the beta value of SMR. * (*p* < 0.05), ** (*p* < 0.005), and *** (*p* < 0.001) represent significant. All *p* values are two sided. For colocalization results, deeper colors indicate higher *SNP.PP.H4* values, and numbers represent the *SNP.PP.H4* values that passed the colocalization analysis threshold (*SNP.PP.H4* > 0.8). (B) Expression of *IREB2* (left) and *PSMA4* (right) in LUAD and LUSC within the TCGA database. Two‐tailed *t*‐test. (C) Prognostic significance of *IREB2* and *PSMA4* expression in lung cancer subtypes: a Kaplan–Meier (KM) survival analysis of total lung cancer, LUAD, and LUSC cohorts.​ (D and E) RNA expression of *IREB2* and *PSMA4* in lung tissues of COPD patients from GSE76925 (D) and in whole blood from ECOPD cohort (E). Data are presented as mean ± SD. Two‐tailed *t*‐test. (F) Smoking status‐dependent peripheral blood expression heterogeneity of *IREB2* and *PSMA4* in the ECOPD cohort. Data are presented as mean ± SD. One‐way ANOVA. (G–I) Bar charts quantifying adjusted mean differences in annual lung function decline between high/low *IREB2* expression groups: pre‐/postbronchodilator FEV_1_ (G) and FVC (H); and between *PSMA4* expression groups: pre‐/postbronchodilator FVC (I) in the ECOPD cohort. ​​Stratification based on median mRNA levels (qRT‐PCR), covariate‐adjusted for age, sex, BMI, smoking status/pack‐years; SEM error bars shown.

SMR analysis revealed *IREB2* and *CHRNA5* (lung) and *PSMA4* (blood) as key candidates. Elevated *IREB2* expression in lung tissue and blood was positively associated with COPD, emphysema, chronic bronchitis, and lung cancer, but inversely with lung function; these results passed *HEIDI* test in lung but not blood. *CHRNA5* showed positive disease associations but failed HEIDI, while *PSMA4* exhibited limited *HEIDI*‐significant SNPs despite large ORs (Figure 4A and Data S27). Colocalization further validated *IREB2* (lung) and *PSMA4* (blood) as shared factors (*SNP.PP.H4>0.8*; Data S28).

TCGA data revealed *IREB2* and *PSMA4* downregulation in LUAD but upregulation in LUSC (Figure 4B). High *IREB2* correlated with improved lung cancer prognosis (nonsignificant in LUSC), high PSMA4 expression correlates with poor prognosis in lung cancer overall but demonstrates a favorable association in LUAD patients​ (Figure 4C). Protein Atlas confirmed elevated IREB2/PSMA4 in both subtypes. High IREB2 associated with improved LUAD prognosis but worse LUSC outcomes (nonsignificant); high PSMA4 linked to worse LUSC prognosis (Figure S6).

*IREB2* expression significantly increased in COPD lung tissue, while *PSMA4* remained unchanged (Figure 4D). *IREB2* inversely correlated with lung function and positively with emphysema; *PSMA4* showed no functional correlation but negative emphysema association (Figure S7A,B). In Early Chronic Obstructive Pulmonary Disease (ECOPD) cohort peripheral blood (*n* = 104; Table S9), no significant *IREB2*/*PSMA4* expression differences existed between controls and COPD, regardless of smoking status (Figures 4E,F). Both genes positively correlated with bronchodilator‐responsive lung function in controls, but not in COPD (Figure S7C,D). Notably, multivariate linear regression analysis—adjusted for age, sex, BMI, smoking status, and index—showed that *IREB2* was positively correlated with the rate of FEV_1_ decline, whereas *PSMA4* was negatively correlated with the rate of FVC decline, suggesting opposing roles in COPD progression (Figure4G‐I  and Table S10).

**FIGURE 5:**
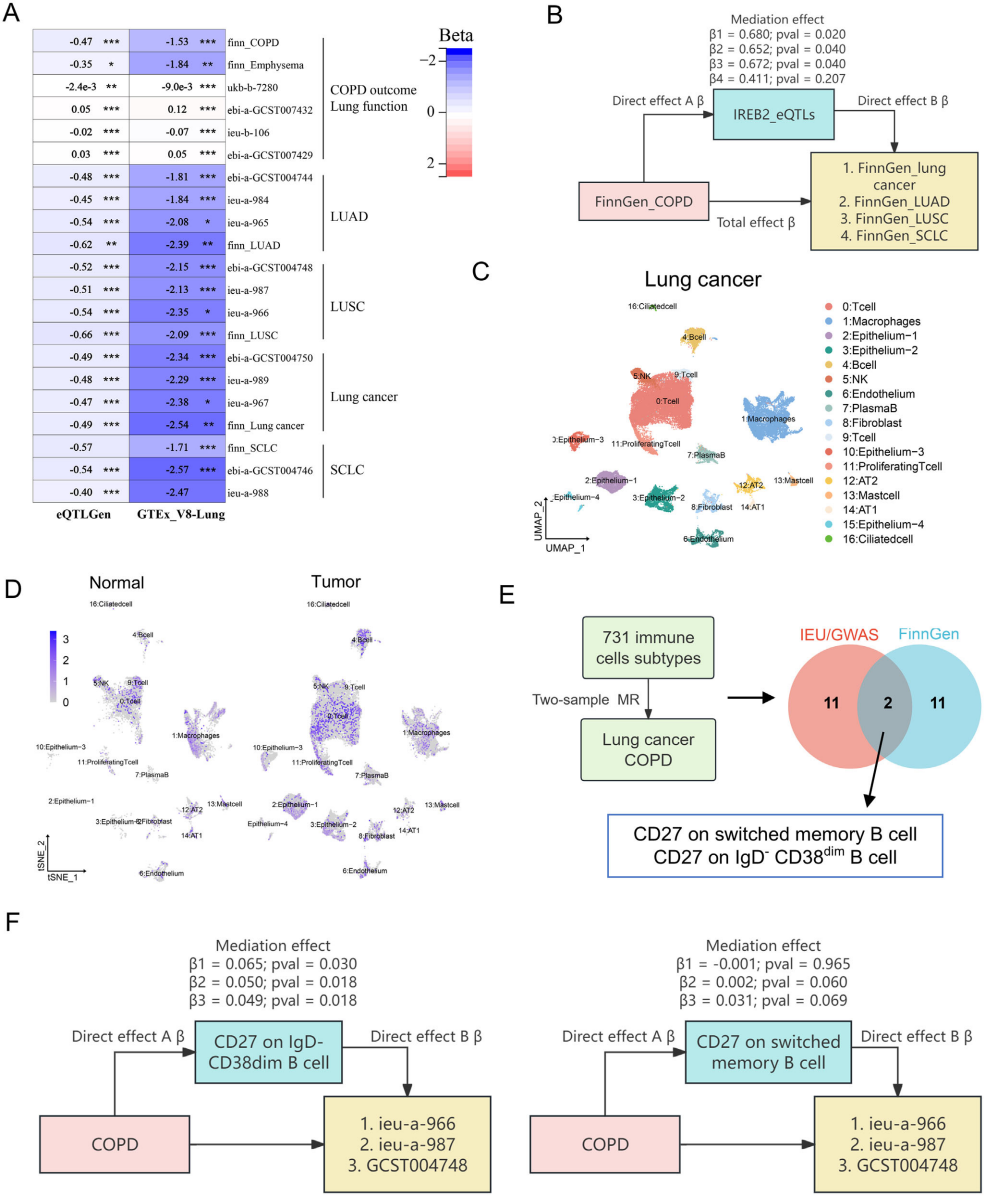
*IREB2* and memory B cells as drivers of COPD progression to lung cancer. (A) Two‐sample MR analysis of *IREB2* in the lung (GTEx_v8 database) and peripheral blood (eQTLGen) for COPD, emphysema, chronic bronchitis, lung function, lung cancer, LUAD, LUSC, and SCLC. * (*p* < 0.05), ** (*p* < 0.01), and *** (*p* < 0.001) represent significance. (B) The diagram illustrates Mendelian randomization assessing IREB2‐eQTLs‐mediated pathways in COPD‐associated lung cancer. Rectangular nodes denote exposure (COPD), mediators (IREB2 eQTLs), and outcome (lung cancer). Directional arrows represent causal effects with annotated parameters: *β* coefficients for mediation effects (*β*1–*β*4) and total effect *β*, alongside corresponding *p* values. (C) UMAP plot illustrating the clustering of cell subpopulations in the single‐cell dataset of NSCLC tumors. (D) UMAP plot showing the expression distribution of IREB2 in adjacent normal and cancerous tissues. (E) This schematic illustrates the analytical pipeline: causality testing of 731 peripheral immune cell traits (exposures) against COPD/lung cancer outcomes (*FinnGen* and *IEU‐GWAS* datasets) using inverse‐variance weighted MR, then Venn diagram identifying replicable causal mediators through cross‐dataset intersection, revealing two concordant features. (F) The diagram illustrates Mendelian randomization assessing CD27 on IgD^−^ CD38 dim B cell (left) or CD27 on switched memory B cell (right) mediated pathways in COPD‐associated lung cancer. Rectangular nodes denote exposure (COPD), mediators (IREB2 eQTLs), and outcome (lung cancer). Directional arrows represent causal effects with annotated parameters: *β* coefficients for mediation effects (*β*1–*β*3), alongside corresponding *p* values.

Two‐sample MR showed that higher *IREB2* expression in lung and blood was negatively associated with COPD, emphysema/chronic bronchitis, and lung cancer, but positively with lung function. In contrast, *PSMA4* expression in blood displayed the opposite pattern (Figure 5A and Data S29), consistent with SMR results.

Two‐step MR (FinnGen) further indicated that *IREB2* mediates COPD‐to‐lung cancer progression (including LUAD and LUSC), supporting its role as a potential driver gene (Figure 5B). This finding was not replicated in the IEU GWAS data. *PSMA4* mediation analyses yielded nonsignificant results, with insufficient SNPs for validation (Data S30). Current evidence therefore highlights *IREB2*, but not *PSMA4*, as a candidate driver in COPD–lung cancer transition.

Human protein Altas IHC database showed elevated IREB2 in LUAD/LUSC (Figure S8A). Gene Set Enrichment Analysis (GSEA) revealed *IREB2* in LUAD positively regulates Hedgehog/TGF‐β pathways but negatively regulates asthma/oxidative phosphorylation. In LUSC, it positively regulates Hedgehog/homologous recombination but negatively regulates IL‐17 signaling, oxidative phosphorylation, and cytokine interactions (Figure S8B). COPD cohorts showed high *IREB2* expression correlating with lung cancer pathways (Figure S8C,D).

cBioPortal identified IREB2 alterations in 1.6% of lung cancers (primarily missense mutations/amplifications; Figure S9A,B). High *IREB2* reduced *TP53/TTN/SYNE1/DNAH8/COL6A3/SYNE2/CDKN2A* mutation frequencies but increased *KRAS/ASTN1/MXRA5* mutations (Figure S9C). Immune infiltration analysis showed *IREB2* negatively correlates with ImmuneScore/ESTIMATEScore in LUSC/LUAD (Figure S10A), with significant immune checkpoint associations (Figure S10B–D).

### 2.6 Memory B Cells Identified as Key Immune Cells in the Progression from COPD to Lung Cancer

We analyzed a public single‐cell RNA‐seq dataset (Prazanowska et al.) in non‐small cell lung cancer (NSCLC) (Figure 5C) [13]. After unsupervised clustering and annotation (Figure S11A and Data S31), *IREB2* was expressed across multiple lu4ng cell types (Figure 5D). Notably, *IREB2* showed differential expression in B cells, endothelial cells, epithelial cells, and macrophages (Figure S11B). Using TIMER2.0, *IREB2* correlated with CD4⁺ T cells, CD8⁺ T cells, B cells, macrophages, monocytes, mast cells, and neutrophils in LUAD/LUSC (Figure S11C), suggesting functional interactions.

 To identify immune subsets driving COPD‐to‐lung cancer progression, two‐sample MR analyzed 731 subsets (Vosa et al.) [14] using IEU GWAS and FinnGen data (Figure S12A,B and Data S32–S34). Overlapping subsets included CD27⁺ switched memory B cells and CD27⁺ IgD^−^ CD38dim B cells (Figures 5E and S12C). Subsequently, COPD positively regulated CD27⁺ IgD^−^ CD38dim B cells, which *further* promoted lung cancer (Data S35). Two‐step MR indicated both subsets partially mediate progression, with significance only for CD27 on IgD^−^ CD38dim B cells in IEU GWAS (Figure 5F and Data S36).

Further GEPIA analysis revealed Decreasing naïve B cells but increasing memory/plasma cells from normal, adjacent normal and tumor tissue (Figure S13A), and increasing CD27/IREB2 in memory/plasma cells across these tissues (Figure S13B,C). High *CD27* mRNA correlated with poor LUAD but improved LUSC prognosis (Figure S14A), while elevated CD27 protein in paired LUAD/LUSC tissues showed no prognostic significance from Clinical Proteomic Tumor Analysis Consortium (CPTAC) datasets (Figure S14B,C).

Moreover, immune infiltration analyses (CIBERSORT/EPIC/MCPcounter/xCELL) showed *IREB2* negatively correlated with B cell function in LUAD/LUSC (Figure S15A–D). Consistently, GEPIA2 revealed *IREB2*'s negative association with memory B cell markers (MS4A1/CD19/CD27/CD80; Figure S15E). Collectively, memory B cells play crucial roles in COPD‐to‐lung cancer progression, with *IREB2* significantly impacting their function.

### 2.7 CD27‐Positive B Cells are Elevated in the Lungs of COPD Patients and May Promote Lung Cancer Development

**FIGURE 6:**
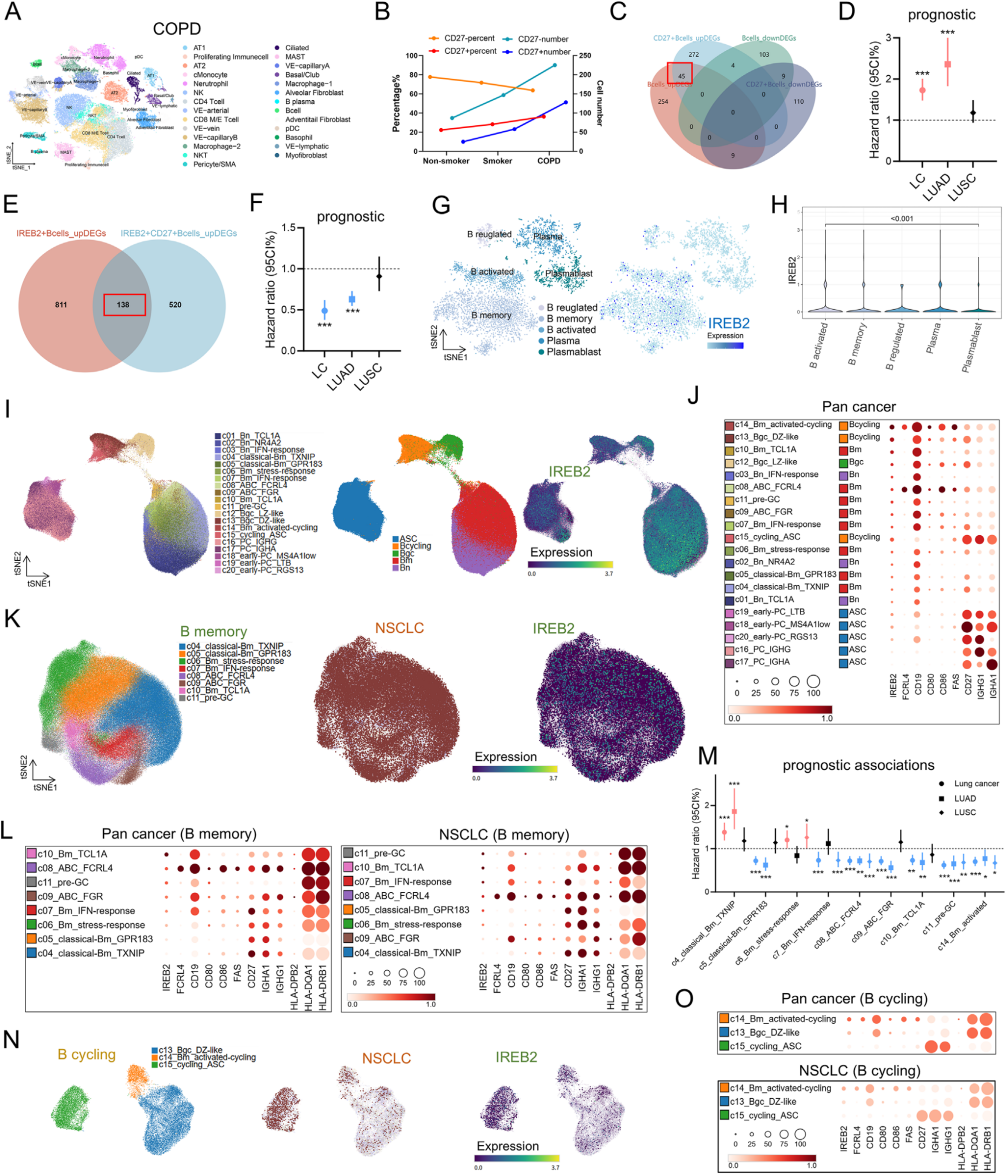
IREB2‐ and CD27‐expressing B cells as pivotal regulatory immune cells driving COPD‐to‐lung cancer progression. (A) UMAP plot illustrating the clustering of cell subpopulations in the single‐cell dataset of COPD patients from GSE173896. (B) Numbers and proportions of CD27‐positive and CD27‐negative B cells in nonsmokers, smokers, and COPD, derived from single‐cell sequencing data from GSE173896. (C) Venn diagram showing the overlap between DEGs in B cells from COPD patients and DEGs between CD27‐positive and CD27‐negative B cells from GSE173896. (D) Prognostic analysis of the signature constructed from 45 overlapping genes between B cells in COPD and CD 27^+^ B cells in lung cancer, LUAD, and LUSC, using KMplot. (E) Venn diagram showing the overlap of upregulated DEGs between *IREB2*‐positive B cells and IREB2+CD27+ double‐positive B cells. (F) Hazard ratios (HR) with 95% confidence intervals for a gene set evaluated in lung cancer, LUAD, and LUSC using KM analysis. Data are presented as bar chart with error bars depicting 95% CIs. Prognostic markers colored blue indicate favorable outcomes. (G) UMAP illustrates the integration and reclustering of B cells and plasma cells extracted from COPD and lung cancer scRNA‐seq datasets into five distinct subsets, with IREB2 expression distribution across these subsets. (H) Violin plots depict differential IREB2 expression among B cell subsets. (I) UMAP presents the integrated pan‐cancer B cell scRNA‐seq dataset annotated with 20 refined B cell clusters and five canonical subsets, alongside IREB2 expression patterns (Cirrocumulus database). (J) Dot plot displaying expression of IREB2 and B cell function/prognosis‐associated genes across pan‐cancer B cell subsets. (K) UMAP illustrates B memory cell subsets within the integrated pan‐cancer B memory cell scRNA‐seq dataset, with specific visualization of the NSCLC compartment and IREB2 expression distribution. (L) Dot plot showing *IREB2* and B cell function/prognosis‐related gene expression in pan‐cancer and lung cancer B memory subsets. (M) Hazard ratios (HR) with 95% confidence intervals for a gene set evaluated in lung cancer, LUAD, and LUSC using KM analysis. Data are presented as bar chart with error bars depicting 95% CIs. Prognostic markers colored blue indicate favorable outcomes. (N) UMAP illustrates B memory cell subsets within the integrated pan‐cancer cycling B cell scRNA‐seq dataset, with specific visualization of the NSCLC compartment and *IREB2* expression distribution. (O) Dot plot illustrating IREB2 and B cell function/prognosis‐linked gene expression in pan‐cancer versus lung cancer B cycling subsets. Data visualization (I, J, K, L, N, O) partly generated by pan‐B (http://pan‐b.cancer‐pku.cn/).

To investigate memory B cells and IREB2, we analyzed single‐cell data from 16 subjects (nine COPD, four smokers, three never‐smokers; Figures 6A and S16A). Memory B cells (CD27⁺) progressively increased from never‐smokers to COPD patients (Figure 6B). COPD B cells showed DEGs enriched in antigen presentation, BCR signaling, and inflammatory pathways (MAPK/NF‐κB/IL‐17/TLR; Figure S16B,C and Data S37), while CD27⁺ subsets were linked to neutrophil degranulation, cytokine signaling, and immune regulation (Figure S17A,B and Data S38). Cancer‐associated genes (*VIM, IGHG1‐4*, *IGKC*, *IGHA1*) were upregulated (Figure S17C), and CPCAT data revealed correlations of IREB2 with IGHG1 and VIM, and CD27 with IGHG1 (Figure S17D). Notably, 45 overlapping DEGs between COPD B cells and CD27⁺ subsets suggested roles in COPD–cancer transition, and this signature predicted poor lung cancer/LUAD prognosis (Figure 6C,D).

CellChat analysis indicated CD27⁺ B cells primarily signal via MIF and BAFF pathways, with unique crosstalk with CD8⁺ T, mast, NK, and NKT cells (Figure S17E,F). Further, ligand–receptor interactions differed: never‐smokers exhibited CD27⁺ B cell interactions via LGALS9–HAVCR2/CD44/CD45 and ANXA1–FPR1/FPR2, whereas smokers utilized CD74‐CXCR4/CD44 pathways linked to cancer progression (Figure S18A–C). This interaction was stronger in COPD CD27⁺ B cells.

In lung cancer specimens, B cell number/proportion increased (Figure S19A), with CD27⁺ subsets enriched (Figure S19B). B cells from COPD and cancer shared biological functions (Figure S19C), and tumor‐infiltrating B cells overexpressed genes (*IGHG1‐4*,* IGKC*, *IGHA1*) consistent with COPD (Figure S19D,E). Pseudotemporal analysis categorized B cells into five states (State_1–5; Figure S20A). CD27⁺ B cells accumulated in State_4 (high *IGHG1‐4/IGKC/VIM/IGHA1*), replacing State_3 (Figure S20B–D). Strikingly, State_4 markers correlated with poor lung cancer prognosis, while State_3 markers associated with better outcomes (Figure S20E and Data S39), implying CD27⁺ B cells differentiate into malignant State_4 subsets.

IREB2 was widely expressed across lung cell types (Figure S21A,B and Data S40). Functional grouping showed immune roles in regulation/inflammation, and structural roles in vascular development, RNA metabolism, and VEGFA–VEGFR2 signaling (Figure S21D–G). In B cells, 138 DEGs were shared by IREB2⁺ and IREB2⁺CD27⁺ cells (Figure 6E), and their signature predicted favorable prognosis in lung cancer and LUAD (Figure 6F), suggesting *IREB2* may suppress malignancy within B cells.

### 2.8 Exploration of IREB2's Regulatory Potential in Tumor B Cells

Integration of COPD and lung cancer B cells identified five subsets, with *IREB2* highest in activated B cells and lowest in regulated subsets (Figures 6G–H and S22A). *IREB2* correlated positively with *CD19, MS4A1, HLA* genes, while *CD27* correlated with *IL‐10*, consistent with reports linking IL‐10⁺ B cells to poor prognosis (Figure S22B) [15].

In a public pan‐cancer B cell dataset [16], *IREB2* was enriched in memory and cycling B cells, positively associated with (Figure 6I,J), positively associated with functional/prognostic markers (*CD19*, *CD80*, *CD86*, *FCRL4*, *FAS*), and negatively with poor‐prognosis genes (*IGHG1*, *IGHA1*, *CD27*), consistent across lung cancer subsets (Figure 6I–O). Prognostic analysis showed *IREB2*‐high memory B cell clusters (c7, c10, c11) associated with better survival, while *IREB2*‐low clusters (c4, c6) correlated with poor outcomes (Figure 6M).

**FIGURE 7:**
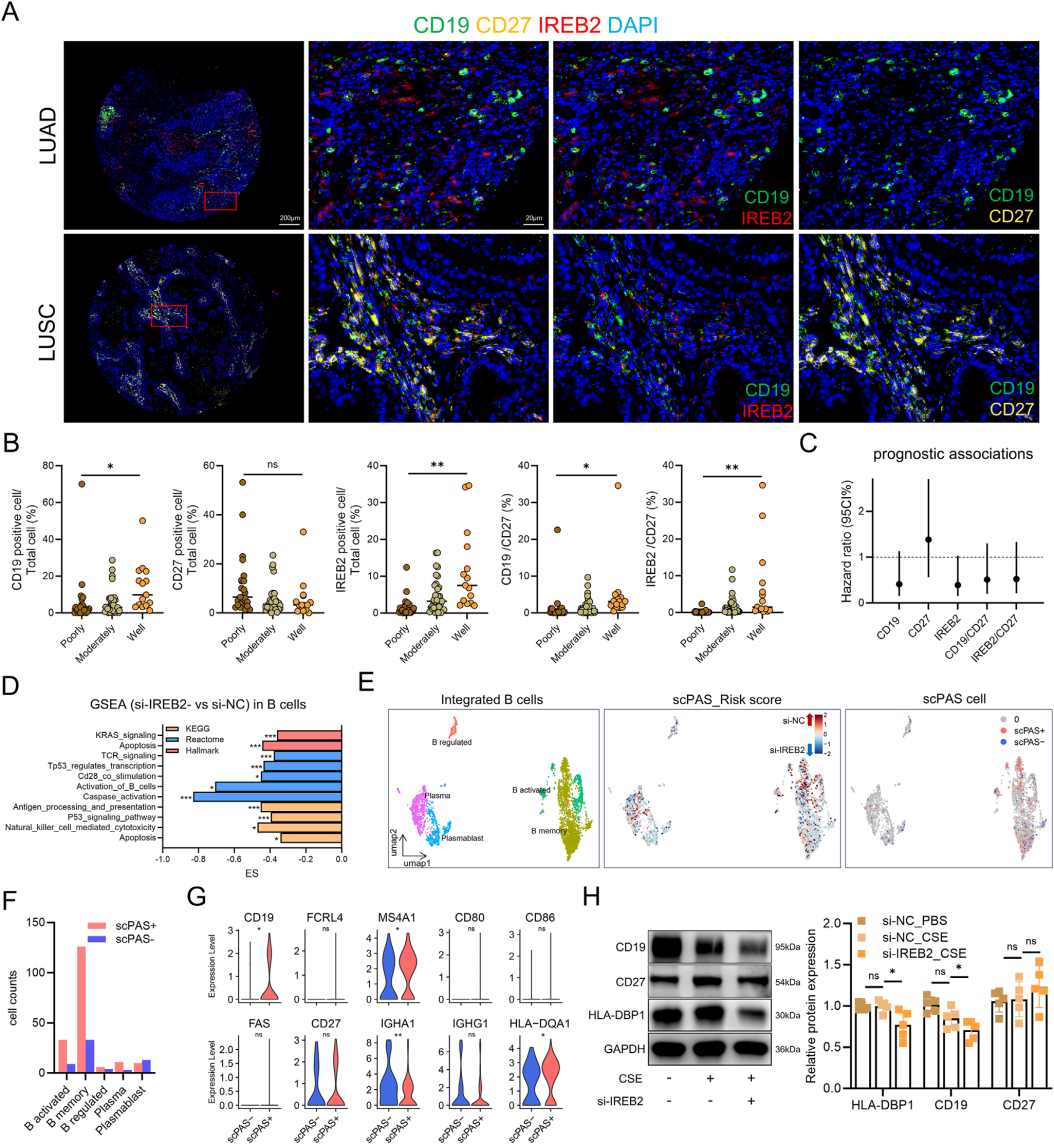
IREB2 exhibits regulatory potential for both intrinsic B cell functions and antigen‐presenting capabilities. (A) Multiplex immunofluorescence demonstrates the expression patterns of *CD19, IREB2*, and *CD27* on lung cancer tissue microarrays. Red: IREB2; green: CD19; yellow: CD27. (B) The proportions of CD19⁺, CD27⁺, and IREB2⁺ cells and the CD19/CD27, IREB2/CD27 ratios across histological grades of lung tumors. (C) Forest plots show prognostic correlations of CD19⁺/CD27⁺/IREB2⁺ cell proportions and CD19/CD27, IREB2/CD27 ratios with survival by Kaplan–Meier analysis. (D) GSEA results showcase KEGG, Reactome, and Hallmark pathway enrichment in si‐IREB2 versus si‐NC in SU‐DHL‐4 cells from RNA sequencing. (E) UMAP visualization of B cells (left), scPAS‐derived risk scores (middle), and scPAS‐selected cells (right) from integration of B cells and plasma cells from COPD and lung cancer scRNA‐seq datasets, comparing si‐NC group versus si‐IREB2 group. Colors reflect risk score gradients (red: high; blue: low). (F) Distribution of scPAS+ and scPAS− cells across B cell subpopulations. (G) Violin plots depict expression distributions of *CD19, FCRL4, MS4A1, CD80, CD86, FAS, CD27, IGHA1, IGHG1*, and *HLA‐DQA1* across scPAS^+^ and scPAS^−^ cell populations.​ (H) Western blot analysis showing changes in protein expression levels of CD19, CD27, and HLA‐DPB1 in SU‐DHL‐4 cells following CSE stimulation and IREB2 knockdown (*n* = 5). Multiple *t*‐tests. Data are presented as mean ± SD.

Multiplex immunofluorescence of a lung tumor tissue microarray (Table S11) showed CD19⁺ and IREB2⁺ cells positively correlated with differentiation, while CD27⁺ cells correlated negatively; CD19⁺/CD27⁺ and IREB2⁺/CD27⁺ ratios also tracked with differentiation, though prognostic differences were not statistically significant (Figures 7A–C and S22C).

To assess *IREB2* function in B cells, we validated siRNA knockdown in SU‐DHL‐4 cells and performed bulk RNA‐seq (Figure S22D–F). GSEA of downregulated DEGs showed enrichment in apoptosis, TCR, TP53, and NK pathways, indicating impaired antigen presentation (Figure 7D). Knockdown also decreased *CD19, CD80, FAS*, and *HLA* family expression while increasing *IGHA1/IGHG1* (Figure S23A).

Single‐cell phenotype‐associated subpopulation identifier (scPAS) analysis integrating bulk‐ and single‐cell data revealed that IREB2 loss mainly affected memory B cells, with scPAS^−^ subsets enriched in tumors (Figures 7E,F and S23B). These cells showed reduced interferon, BCR, and TCR signaling (Figure S23C). Compared with scPAS^−^ cells, scPAS⁺ cells expressed higher *CD19, MS4A1*, and *HLA‐DQA1*, but *CD27* was unchanged (Figure 7G). IREB2 knockdown in SU‐DHL‐4 B cells reduced mRNA levels of *MS4A1, CD80, CD86, FAS, FCRL4*, and *HLA‐DQA1* and lowered CD19 and HLA‐DPB1 protein, while CD27 remained unaffected (Figures 7H and S23D). These findings suggest that IREB2 can modulate B cell function, yet it exerts no significant effect on CD27.

## 3 Discussion

This study established COPD as a significant risk factor for lung cancer. Using multimodal evidence, we identified COPD as a potential precursor to lung cancer. We explored underlying molecular mechanisms and immune cell roles in this relationship. Key genetic loci and genes, including *IREB2*, were implicated as potential drivers of COPD progression to lung cancer. Critically, we observed elevated CD27+ B cells in COPD, suggesting their contribution to cancer progression via inflammatory response modulation and lung microenvironment changes, mirroring their role in lung cancer. These findings underscore the immune‐cancer interplay in COPD patients.

The COPD–lung cancer link is well established. Epidemiological evidence confirms higher lung cancer incidence in COPD patients, consistent with our findings [6]. Shared risk factors like smoking and pollution contribute to this association [17]. Additionally, COPD‐associated chronic inflammation and airway remodeling foster a cancer‐conducive microenvironment [5].

Collectively, COPD heterogeneity and lung cancer polygenicity are recognized [18]. Our findings confirm distinct genetic loci for COPD and lung cancer via TWAS, with minimal overlap. Enrichment indicates shared loci have tissue‐specific functional profiles in blood versus lung, highlighting pathogenic gene heterogeneity. Conversely, SMR showed distinct COPD and lung cancer loci could induce comparable biological changes, potentially critical for progression. Two‐sample bidirectional MR, mediator MR, and colocalization collectively support COPD as a causal factor in lung cancer. Notably, reverse MR suggested a weaker potential causal link from lung cancer to certain COPD traits.

This study identifies shared COPD and lung cancer genetic loci and genes using multiple methods. MR, colocalization, and TWAS pinpointed loci consistently associated with *IREB2*, *CHRNA3*, *CHRNA5*, and *PSMA4* expression. SNPs linked to these genes are reported in both diseases. For example, Hopkins et al. [11] associated the *CHRNA5* rs16969968 AA genotype with COPD and lung cancer; Nedeljkovic et al. linked chromosome 15q25.1 variants to COPD [19]; and Zhang et al. [20] connected rs16969858 in *IREB2* to COPD. While SNPs differed from our study, the shared target genes support our findings, suggesting the genetic risk involves multiple variants rather than one critical SNP. Given their roles as nicotine receptors, *CHRNA3* and *CHRNA5* may explain associations with smoking‐related diseases like COPD and lung cancer [21]. Notably, *IREB2* and *PSMA4* showed significant eQTLGen SMR results for both diseases, positioning them as potential key shared genes. Furthermore, *PSMA4* consistently demonstrated significance in peripheral blood across analyses, suggesting a potential role in its biology. However, their protein‐level relationship with COPD and lung cancer remains unassessed due to absent pQTL data in UKB.

Identified earlier, the IREB2 gene exhibits elevated RNA and protein levels in COPD [22], Although ECOPD cohort data showed no significant peripheral blood IREB2 change in COPD patients, its levels positively correlated with lung function decline rate, suggesting progression linkage. ​Furthermore,​​ IREB2 polymorphisms, notably rs2568494, was associatde with COPD and lung cancer risk and reduced lung function. Its polymorphisms are closely associated with the risk of developing COPD and lung cancer, and may also contribute to reduced lung function, with rs2568494 being the most frequently reported SNP [21, 23, 24]. While most COPD GWAS suggest protective IREB2 mutations, we found novel IREB2 SNPs linked to lung cancer in COPD patients, implying its potential role as a driver gene in COPD progression to lung cancer. Notably, while our findings position IREB2 as a driver of COPD‐to‐lung cancer progression, paradoxically, several reports suggest high IREB2 expression in established lung cancer may associate with improved prognosis. This suggests potential dual functionality: IREB2 may promote early carcinogenesis yet exert tumor‐suppressive effects in established malignancies.​ IREB2 regulates intracellular iron homeostasis by binding iron‐responsive elements (IREs) on ferritin transcripts and iron metabolism‐related genes [25]. Despite potential importance, few studies explore *IREB2* roles in COPD and lung cancer [26]. Its iron metabolism involvement may influence oxidative stress and apoptosis. Notably, Xia et al. [27] reported IREB2 can promote apoptosis, *IREB2* dysfunction may contribute to tumor cell proliferation. Our in vitro experiments validated that IREB2 impairment in B cells, particularly memory B cells, downregulates genes related to antigen presentation and T‐cell help, resulting in impaired antigen presentation. ​Critically, given differential IREB2 expression across COPD and lung cancer cell subtypes, its effects may extend beyond memory B cells. However, its role in other lung cancer cell subpopulations requires further study.

Brandsma et al. [28] identified increased memory B cells in COPD patients. Furthermore, IgA^+^ B cells and locally impaired IgA secretion link to COPD small airway inflammation [29]; Habener et al. [30] also associated memory B cells with asthma and small airway disease. ​However, the precise role of B cells in COPD progression and lung cancer pathogenesis remains unclear. Notably, while lung cancer immunobiology emphasizes macrophages and T cells, Hao et al. [31] observed that memory B cells, particularly CD27^+^ types, showed highest B cell receptor characteristic scores and were exclusively in early‐stage NSCLC tumors, suggesting a procancer role. Furthermore, COPD patients exhibit higher proportions of B cells, including memory cells. Several B‐cell malignancies derive from memory B cells [32]; ​however, some memory B‐cell subsets have been associated with improved prognosis [16]. Thus, elucidating their influence on COPD progression and lung cancer development warrants further research.

Single‐cell sequencing revealed these cells express IGHG family and IGHA1—immunoglobulins linked to cancer progression and metastasis [33]. Moreover, IgA^+^ memory B cells associate with exacerbated small airway dysfunction [29]. ​Notably, stimulated CD27^+^ cells interact via CD74–CD44 and CD74–CXCR4 pathways, which enhance malignant cell viability/migration and promote immune escape through NK cell modulation [34, 35, 36]. ​Collectively, these findings connect CD27^+^ memory B cells to respiratory disease symptoms, supporting their procancer potential, though their exact mechanisms within the lung microenvironment require further study.

MR demonstrated a robust causal link between COPD and lung cancer, while some individual GWAS lacked statistical significance due to population heterogeneity, highlighting the necessity of multicenter GWAS to ensure stable and reliable results. Despite​ associations between emphysema, chronic bronchitis, lung function changes, and lung cancer in our cohort after confounder adjustment, and confirmed genetic correlations via LDSC, bidirectional MR revealed significant causality solely between FEV_1_ and LUSC. No causal links were evident for emphysema, chronic bronchitis, or FVC with lung cancer. ​Conversely, reverse MR indicated lung cancer causally influences certain COPD‐related genetic data, including emphysema, chronic bronchitis, and lung function alterations. This suggests lung cancer may impact respiratory physiology, exacerbate symptoms, and contribute to common respiratory diseases, aligning with our proposed “oncology‐respirology” concept [37]. Thus, even successfully treated early‐stage lung cancer patients warrant ongoing monitoring of lung function, respiratory symptoms, and pulmonary pathology. Early intervention should be considered if chronic bronchitis/emphysema develop or lung function declines. In addition, for COPD patients, prevention mainly relies on smoking cessation and avoidance of carcinogenic exposures, while early detection depends on low‐dose CT screening with risk stratification models [38]; genetic markers are promising but remain investigational and require validation in large cohorts.

While​ providing valuable COPD–lung cancer insights, limitations exist. NHANES’ cross‐sectional design precluded definitive causal inferences despite MR's utility. ​Critically, exclusively European GWAS cohorts limit Asian generalizability. Furthermore, the COPD–lung cancer causal relationship is intricate and multifactorial (e.g., smoking, environmental exposures, genetics). Despite rigorous control efforts, confounding effects remain challenging. Additionally, while single‐cell sequencing yielded insights, limited sample size may constrain capturing all biological alterations. Finally, though key genes/cell subtypes were identified, mechanisms driving COPD‐to‐lung cancer transition remain highly complex. While preliminary in vitro phenotypic exploration of IREB2 in B cells was conducted, its functions in other cellular subsets remain unexplored, and corresponding in vivo functional validation is lacking. Additionally, our findings indicate that IREB2 may exert stage‐ and cell type‐specific effects in COPD and lung cancer. While genetic and functional data suggest a role in COPD susceptibility through oxidative stress and iron regulation [39], other evidence implies potential compensatory effects in tumor contexts, possibly via ferroptosis modulation or immune regulation [40]. These interpretations remain provisional and require further experimental validation to fully elucidate IREB2's mechanistic roles.

Collectively, this study offers novel insights into COPD–lung cancer links. Our integrated analyses elucidated disease associations, identifying ​IREB2‐regulated CD27^+^ memory B cells​ as potential COPD progression risk factors. Single‐cell sequencing revealed their mechanistic contributions, advancing understanding of disease pathogenesis and providing foundations for future research/therapeutics. ​Clinically, these findings warrant enhanced monitoring in COPD patients to mitigate lung cancer risk.

## 4 Materials and Methods

### 4.1 Study Design and Population

The NHANES, conducted by the National Center for Health Statistics at the Centers for Disease Control and Prevention (https://www.cdc.gov/nchs/nhanes/index.htm), is a nationally representative, cross‐sectional survey of the civilian, noninstitutionalized US population with data released in 2‐year cycles. This study utilized data from nine cycles (1999–2000 to 2015–2016) for chronic bronchitis and emphysema analysis, combining data after excluding participants missing information on these conditions (*n* = 8627), smoking status (*n* = 45), or BMI (*n* = 999). A final sample of 46,111 participants (29,084 aged 40+ years) was included. For doctor‐diagnosed COPD analysis, data from two cycles (2013–2014, 2015–2016) was used, excluding participants missing information on physician‐diagnosed COPD (*n* = 2671), smoking status (*n* = 12), or BMI (*n* = 135). This resulted in a final sample of 10,905 participants (*n* = 7193 aged 40+ years). Finally, the lung function analysis combined data from three cycles (2007–2008 to 2011–2012), excluding participants missing information on lung function (*n* = 2671), smoking status (*n* = 3417), or BMI (*n* = 65). The final sample for lung function analysis included 13,313 participants (8305 aged 40+ years).

### 4.2 GWAS Summary Statistics for Lung Cancer, COPD, Emphysema/Chronic Bronchitis, and Lung Function

In this study, we compiled GWAS data for various lung cancer subtypes, including lung cancer overall, LUAD, LUSC, and SCLC. These data were obtained from diverse sources such as TRICL [41], ILCCO [42], and the International Agency for Research on Cancer, World Health Organization, via the UK Biobank. Additionally, we used GWAS data for COPD (FEV_1_/FVC < 0.7) [43] from the GWAS catalog (https://www.ebi.ac.uk/gwas/), as well as data on FEV_1_, FVC, emphysema/chronic bronchitis, and “ever smoked” from the *IEU GWAS data* (https://gwas.mrcieu.ac.uk/) [44]. *FinnGen R10* (https://r10.finngen.fi/) provided independent validation data for NSCLC, LUAD, LUSC, SCLC, COPD, emphysema, and chronic bronchitis. All​ summary‐level data derived from public GWAS Catalog, FinnGen, and IEU GWAS sources (Data S1). Participants were European‐ancestry with 1000 Genomes‐imputed genotypes. ​This research used exclusively public GWAS summary statistics.​​ All participants provided written informed consent with cohort‐specific ethics approval.

### 4.3 Assessing Local SNP‐Heritability and Genetic Correlations by ρ‐HESS Method

The ρ‐HESS method [45] was employed to evaluate local SNP heritability and local genetic correlations from summary GWAS data. This method divides the genome into 1703 regions, each with an average size of approximately 1.5 MB. Using the 1000 Genomes Project European reference based on the hg19 genome build [46], ρ‐HESS estimates local genetic heritability for each trait and the genetic covariance between traits, subsequently calculating local genetic correlations from these heritability and covariance estimates.

### 4.4 Statistical Analysis

Statistical analyses were performed using SPSS (version 27.0), GraphPad Prism version 8.0.1 and R version 4.2.2. Logistic regression evaluated risk factors; multivariable models adjusted for age, sex, BMI, race, smoking status; Firth Logistic regression addressed rare outcomes in propensity‐matched cohorts. Differences between paired means were assessed using a two‐tailed Student's *t*‐test and one way‐ANOVA with significance levels indicated as follows: ns (not significant) for *p* ≥ 0.05, *p < 0.05, **p < 0.01, and ***p < 0.001.

## Author Contributions

Erkang Yi and Qingyang Li conceived and designed the experiments. Yu Liu, Fan Wu, Ruiting Sun, Xinqing Lin, and Xiaohong Xie performed the cohort analysis and cancer analysis. Hairong Wang performed cell experiment. Erkang Yi, Chengshu Xie, Erping Long, and Xuanyi Lu performed bioinformatic analyses. Erkang Yi, Qingyang Li, and Wenqian Wu wrote the manuscript and analyzed the data. Erkang Yi, Qingyang Li, Yumin Zhou, Chengzhi Zhou, and Pixin Ran supervised the research and acquired the funding. Erkang Yi, Qingyang Li, Wenqian Wu, and Chengshu Xie contributed equally to this paper. All authors have read and approved the final manuscript.

## Funding Information

This study received grants from the National Natural Science Foundation of China (Nos. 82200045, 82270043 and 81970045), the Foundation of Guangzhou National Laboratory (No. SRPG22‐016), the Youth Foundation of the National Key Laboratory of Respiratory Diseases (No. SKLRD‐Z‐202326), the Plan on Enhancing Scientific Research in Guangzhou Medical University (No. GMUCR2024‐01012), the Guangzhou Science and Technology Plans (No. 202201020372), Noncommunicable Chronic Diseases‐National Science and Technology Major Project (No. 2024ZD0528400), and Major Project of Guangzhou National Laboratory (No. GZNL2023A02001).

## Conflicts of Interest

The authors declare no conflicts of interest.

## Ethics Statement

Peripheral blood samples from COPD patients and healthy controls were collected through the ECOPD study (Trial registration: Chinese Clinical Trial Registry, ChiCTR1900024643; registered July 19, 2019). Written informed consent was obtained from all human participants prior to sample collection.

## Supporting information




**Figure S1**: (A) Inclusion and exclusion process for the population analyzed in the NHANES database. The populations include those with chronic bronchitis and emphysema (1999–2016), physician‐diagnosed COPD (2013–2016), and individuals with lung function data (2007–2012). Individuals without information on emphysema, chronic bronchitis, COPD, lung function, smoking status, or BMI were excluded. The final analysis included individuals aged 20–80 years. Subsequently, those aged 20–39 years were excluded, resulting in a final analysis cohort of individuals aged 40–80 years. (B) Whole‐genome genetic correlations of COPD, emphysema, chronic bronchitis, and lung function in IEU GWAS (B) and FinnGen GWAS (C) with lung cancer using HDL. Colors represent the magnitude of the genetic correlation of COPD, emphysema, chronic bronchitis, and lung function with lung cancer (lung cancer, LUAD, LUSC, and SCLC), using LDSC, with red indicating positive genetic correlation, blue indicating negative genetic correlation and white indicating low true heritability where results could not be calculated. Numbers represent the genetic correlation. * (*p* < 0.05), ** (*p* < 0.005) and *** (*p* < 0.001) represent significant genetic correlation. All *p* values are two sided.
**Figure S2**: (A) Forest plot of two‐sample MR analyses between ever smoked and lung cancer including LUAD and LUSC before and after removing the mediator effect of COPD. Effect sizes (Beta, 95% CI) are shown as the standard deviation change in lung cancer per standard deviation increase in ever smoked. Points on the forest plot represent effect size estimates, while whiskers denote 95% CIs. All *p* values are two‐side. (B) Enrichment and PPI network analysis of overlapping genes from TWAS of Emphysema and lung cancer in lung tissue and peripheral blood using Metascape. (C) Enrichment and PPI network analysis of overlapping genes from TWAS of chronic bronchitis and lung cancer in lung tissue and peripheral blood using Metascape.
**Figure S3**: (A) Volcano plot illustrating the SMR results of COPD and lung cancer using eQTLGen database. (B and C) Upset plot displaying the overlapping genes in the SMR results for COPD and lung cancer using *eQTLGen* (B) and *UKB‐PPP* database (C).
**Figure S4**: (A and B) Enrichment analysis results of SMR eQTLs for COPD and lung cancer, derived from eQTLGen (A), and pQTLs from *UKB‐PPP* (B), using the Metascape database.
**Figure S5**: (A–C) The local heritability and genetic correlation between COPD and Lung Cancer including *ieu‐b‐106* and *ieu‐a‐987* (A), *ieu‐b‐106* and *GCST004748* (B) and *FinnGEN_COPD* and *FinnGen lung cancer* (C) across the genome. The top two panels display the local heritability estimates for COPD and lung cancer, where blue indicates statistically significant positive correlation and red indicates statistically significant negative correlation. The third panel shows local genetic covariance, with blue indicating positive covariance and red indicating negative covariance.
**Figure S6**: (A–D) Protein‐level expression of *IREB2* (A) and *PSMA4* (B) in LUAD and LUSC, and its survival prognosis (C and D), utilizing data from the PCAS database.
**Figure S7**: (A and B) Association analyses of IREB2 and PSMA4 expression in lung tissue with pulmonary function parameters (FEV₁%predicted and FEV₁/FVC%) (A) and the emphysema index (LAA950%) (B) from the GSE76925 dataset. Pearsons *correlation* *analysis*. (C) Association analyses of IREB2 and PSMA4 expression in peripheral blood with pre‐ and postbronchodilator FEV₁ and FVC in control subjects and COPD patients from the ECOPD cohort. Pearsons *correlation* *analysis*.
**Figure S8**: (A) Immunohistochemistry results of *IREB2* in LUAD and LUSC, utilizing data from the HPA database. (B) KEGG analysis results of *IREB2* in LUAD and LUSC. (C and D) KEGG (C) and Reactome (D) enrichment results of *IREB2* in lung tissue sequencing from COPD patients using GSEA from GSE76925.
**Figure S9**: (A and B) The mutational plot for *IREB2* in lung cancer, visualized with variant type, classification, and frequently mutated genes, using data from the cBioPortal database. (C) Waterfall plot of the top 10 mutation genes associated with IREB2 in LUAD and LUSC. Two‐tailed *t*‐test, * (*p* < 0.05), ** (*p* < 0.01), *** (*p* < 0.001), **** (*p* < 0.001).
**Figure S10**: (A) Analysis of the relationship between IREB2 and immune infiltration in LUAD and LUSC using ImmunoScore, StromaScore, and ESTIMATEscore. Pearsons correlation analysis. (B–D) Correlation of *IREB2* with immune immunomodulation‐related genes (B), RNA methylation‐related genes (B) and Immune checkpoint‐related genes (C) in LUAD and LUSC. Pearsons correlation analysis.
**Figure S11**: (A) Dotplot represented the top 3 marker genes for each cell subpopulation in the single‐cell sequencing dataset. (B) Violin plots visualize the distribution of IREB2 expression across cellular subclusters in tumor and adjacent normal tissues from lung cancer scRNA‐seq data. Two‐tailed *t*‐test. (C) Correlation analysis of *IREB2* with the degree of immune cell infiltration in LUAD and LUSC using the TIMER2.0 database. Pearsons correlation analysis.
**Figure S12**: (A and B) Volcano plot showing the two‐sample MR results for 731 immune cells in COPD (A) and lung cancer (B). (C) Venn diagram illustrating the screening process for identifying shared immune cells in COPD and lung cancer based on two‐sample Mendelian randomization results for 731 immune cells.
**Figure S13**: (A) Relative abundance of memory B cells, naive B cells and plasma cells in LUSC, LUAD and normal lung tissues, using data from the GEPIA database. One‐way ANOVA analysis. (B) Relative abundance of *IREB2* in memory B cells, naive B cells and plasma cells in LUSC, LUAD, and normal lung tissues. One‐way ANOVA analysis. (C) Relative abundance of *PSMA4* in memory B cells, naive B cells and plasma cells in LUSC, LUAD, and normal lung tissues. One‐way ANOVA analysis.
**Figure S14**: (A) Kaplan–Meier plots showing the prognostic significance of CD27 in lung cancer including LUAD and LUSC, using data from the KMplot database. (B) Protein expression profiles of CD27 in tumor versus adjacent normal tissues from LUAD and LUSC samples derived from the *CPTAC* database. Two‐tailed *t*‐test, * (*p* < 0.05), *** (*p* < 0.001). (C) Kaplan–Meier survival curves illustrating the prognostic value of CD27 protein expression levels in LUAD and LUSC cohorts, analyzed using CPTAC datasets.
**Figure S15**: (A–D) Correlation analysis of *IREB2* with B cell immune infiltration in LUAD and LUSC using CIBERSORT (A), EPIC (B), MCPcounter (C), and xCELL (D) scoring. Pearsons correlation analysis. (E) Correlation analysis of *IREB2* with memory B cell marker genes (*CD19, MS4A1, CD27*, and *CD80*) in LUAD and LUSC using the GEPIA2 database. Pearsons correlation analysis.
**Figure S16**: (A) UMAP plot illustrating the clustering of cell subpopulations in the single‐cell dataset of COPD patients from GSE173896. (B) Volcano plot showing the DEGs in B cells between COPD patients and smokers. (C) KEGG enrichment results of DEGs in B cells between COPD patients and smokers.
**Figure S17**: (A) Volcano plot showing the DEGs in B cells between COPD patients and smokers. (B) Volcano plot showing the DEGs between CD27‐positive and CD27‐negative cells. (C) Dotplot showing the expression of *VIM, IGHG1, IGHG2, IGHG3, IGHG4, IGKC*, and *IGHA1* in CD27‐positive and CD27‐negative cells from GSE173896. (D) Correlation analysis of protein expression levels of IREB2, CD27, IGHG1, and VIM in tumor and paired adjacent nontumor tissues from LUAD and LUSC was performed using the CPCAT database. Pearson correlation analysis. (E) Analysis of secretory‐type intercellular interactions among various cell types within CD27+ and CD27− B cell clusters, including the degree of enrichment of incoming and outgoing signaling pathways, as identified in single‐cell transcriptomes. (F) Network graphs comparing pulmonary cell interactions in CD27− (top) and CD27+ (bottom) B cells as ligand cells. Each vertex represents a cellular subpopulation; edges signify ligand–receptor interactions. The thickness of the edges quantifies the cumulative expression of ligand–receptor genes, while the size of each vertex reflects Kleinberg centrality, indicating the cell's role in signaling. Cellular subpopulations are differentiated by color and number.
**Figure S18**: (A) Bubble chart visualizing cell‐to‐cell interactions involving CD27− or CD27+ B cells with other cell types sourced from GSE173896. B cells function as ligand‐expressing cells in these interactions. ① and ② Significant ligand–receptor interactions between CD27− and CD27+ B cells. (B and C) Bubble chart visualizes cell‐to‐cell interactions including LGALS9, ANXA1 (B) and CD74 (C) involving CD27− or CD27+ B cells with other cell types cells in nonsmokers, smokers and COPD, sourced from GSE173896. B cells function as ligand‐expressing cells in these interactions.
**Figure S19**: (A) Numbers and proportions of B cells in cancerous and adjacent normal tissues, derived from single‐cell sequencing data of lung cancer. (B) Numbers and proportions of CD27‐positive and CD27‐negative B cells in cancerous and adjacent normal tissues, derived from single‐cell sequencing data of lung cancer. (C) Overlap between gene lists of upregulated DEGs in B cells from COPD and lung cancer: purple curves link identical genes, and blue curves link genes that belong to the same enriched ontology term. (D) Enrichment analysis results of upregulated DEGs in B cells from COPD and lung cancer, using Metascape. (E) Average expression and percent expressed of *IGHG1, IGHG2, IGHG3, IGHG4, IGKC*, and *IGHA1* in B cells from adjacent normal and cancerous tissues, derived from single‐cell sequencing data of lung cancer.
**Figure S20**: (A) Pseudotime trajectory analysis illustrates the developmental progression of B cells from single‐cell sequencing data of lung cancer, color‐coded by five distinct states. (B and C) Pseudotime trajectory analysis delineates the developmental progression of CD27+ and CD27− B cells (B) and the cell counts and relative proportions of CD27+ and CD27− B cells within five distinct states of pseudotime trajectory (C). (D) Average expression and percent expressed of IGHG1‐4, IGKC, VIM, and IGHA1 in five distinct states of pseudotime trajectory of B cells. (E) Prognostic analysis of the top 10 markers of State_3 and State_4 B cells identified by pseudotime trajectory in lung cancer, LUAD, and LUSC using KMplot.
**Figure S21**: (A) UMAP plot showing the expression distribution of IREB2 across cell subpopulations from the GSE173896 dataset. (B) Dotplot showing the differential expression of IREB2 across different cell subpopulations in nonsmokers, smokers, and COPD patients. (C) Fold change plot of DEGs between IREB2‐positive and IREB2‐negative cells across various cell subpopulations. (D and E) Enrichment analysis results of up‐regulated DEGs in IREB2‐positive and IREB2‐negative pulmonary immune (D) and structural cells (E), using the Metascape database. (F and G) Enrichment analysis results of downregulated DEGs in IREB2‐positive and IREB2‐negative pulmonary immune (F) and structural cells (G), using the Metascape database.
**Figure S22**: (A) Dot plot delineating marker gene expression profiles characteristic of distinct cellular subpopulations within integrated B cells. (B) Pearson correlation analysis evaluating associations between IREB2 and B cell function/prognosis‐related genes in integrated B cells. (C) Pearson correlation statistics quantifying relationships of CD19⁺/CD27⁺/IREB2⁺ cell proportions and CD19/CD27, IREB2/CD27 ratios with maximum tumor diameter. (D) qRT‐PCR validation of IREB2 knockdown efficiency using three distinct siRNAs in SU‐DHL‐4 cells. (E) Volcano plot exhibiting differentially expressed genes in si‐IREB2 versus si‐NC treated SU‐DHL‐4 cells from RNA sequencing. (F) Principal component analysis (PCA) plot illustrating sample distribution of si‐NC and si‐IREB2 groups (left), with outlier‐excluded clustering after quality control (right).
**Figure S23**: (A) Heatmap depicting the expression of selected genes in RNA‐seq data from si‐NC and si‐IREB2 groups. (B) Proportions of scPAS⁺ and scPAS^−^ B cells in adjacent nontumor versus tumor tissues. (C) KEGG and Reactome pathway enrichment analyses of genes differentially expressed between scPAS⁺ and scPAS^−^ cells. (D) qRT‐PCR assessment of mRNA levels of CD27, MS4A1, CD80, CD86, FAS, FCRL4, HLA‐DPB1, and HLA‐DQA1 in SU‐DHL‐4 cells following IREB2 knockdown (*n* = 5). Multiple t‐tests.
**Supplementary Table 1**: The weighted demographics and prevalence of COPD and lung cancer from 2013 to 2016 from NHANES.
**Supplementary Table 2**: The weighted demographics and prevalence of emphysema/chronic bronchitis and lung cancer from 1999 to 2016 from NHANES,
**Supplementary Table 3**: The weighted demographics, lung function, and prevalence of lung cancer from 2007 to 2012 from NHANES.
**Supplementary Table 4**: Weighted demographics and prevalence of lung cancer from 2013 to 2016 in NHANES after performing PSM analysis for COPD status.
**Supplementary Table 5**: Weighted demographics and prevalence of lung cancer from 2013 to 2016 in NHANES after performing PSM analysis for chronic bronchitis status.
**Supplementary Table 6**: Weighted demographics and prevalence of lung cancer from 2013 to 2016 in NHANES after performing PSM analysis for emphysema status.
**Supplementary Table 7**: Weighted demographics and prevalence of lung cancer from 2013 to 2016 in NHANES after performing PSM analysis for COPD diagnosed by lung function.
**Supplementary Table 8**: Odds ratio (OR) analysis of COPD, emphysema, chronic bronchitis, lung function, and lung cancer
**Supplementary Table 9**: Demographic and clinical baseline characteristics of peripheral blood specimens in the ECOPD cohort.
**Supplementary Table 10**: Multiple linear regression analysis​ of peripheral blood IREB2 and PSMA4 expression ​versus​ lung function decline rate ​in the ECOPD cohort.
**Supplementary Table 11**: Demographic and clinical baseline characteristics of peripheral blood specimens in the ECOPD cohort.
**Supplementary Table 12**: Primers used in experiments.

Supporting File 2: mco270473‐sup‐0002‐SuppMat.xlsx

## Data Availability

All transcriptome sequencing data referenced in our manuscript are available in the CNCB‐NGDC database, with the corresponding OMIX IDs: OMIX011091. The datasets supporting the conclusions of this article are available in online repositories. Names of repositories/repositories and accession number(s) are provided in the article or in the Supporting Information. All other relevant materials are available from the corresponding author upon reasonable request.
