# Supplementary material for: Integrative GWAS and Mendelian Randomization Analysis Identifies IREB2 and CD27+ Memory B Cells as Core Drivers of COPD to Lung Cancer Progression
Source: MedComm (2020). 2025 Dec 8;6(12):e70473. doi: 10.1002/mco2.70473 (PMC12685611; doi:10.1002/mco2.70473)
Supplement: Supplementary file 1 — Figure S1: (A) Inclusion and exclusion process for the population analyzed in the NHANES database. The populations include those with chronic bronchitis and emphysema (1999–2016), physician‐diagnosed COPD (2013–2016), and individuals with lung function data (2007–2012). Individuals without information on emphysema, chronic bronchitis, COPD, lung function, smoking status, or BMI were excluded. The final analysis included individuals aged 20–80 years. Subsequently, those aged 20–39 years were excluded, resulting in a final analysis cohort of individuals aged 40–80 years. (B) Whole‐genome genetic correlations of COPD, emphysema, chronic bronchitis, and lung function in IEU GWAS (B) and FinnGen GWAS (C) with lung cancer using HDL. Colors represent the magnitude of the genetic correlation of COPD, emphysema, chronic bronchitis, and lung function with lung cancer (lung cancer, LUAD, LUSC, and SCLC), using LDSC, with red indicating positive genetic correlation, blue indicating negative genetic correlation and white indicating low true heritability where results could not be calculated. Numbers represent the genetic correlation. * (p < 0.05), ** (p < 0.005) and *** (p < 0.001) represent significant genetic correlation. All p values are two sided. Figure S2: (A) Forest plot of two‐sample MR analyses between ever smoked and lung cancer including LUAD and LUSC before and after removing the mediator effect of COPD. Effect sizes (Beta, 95% CI) are shown as the standard deviation change in lung cancer per standard deviation increase in ever smoked. Points on the forest plot represent effect size estimates, while whiskers denote 95% CIs. All p values are two‐side. (B) Enrichment and PPI network analysis of overlapping genes from TWAS of Emphysema and lung cancer in lung tissue and peripheral blood using Metascape. (C) Enrichment and PPI network analysis of overlapping genes from TWAS of chronic bronchitis and lung cancer in lung tissue and peripheral blood using Metascape. Figure [file MCO2-6-e70473-s002.docx]

Title

Integrative GWAS and Mendelian Randomization analysis identifies IREB2 and CD27+ memory B cells as core drivers of COPD to lung cancer progression

Authors

Erkang Yi ^1,^^2#^, Qingyang Li^1#^, Wenqian Wu^3#^, Chengshu Xie^2^, Hairong Wang^2^, Erping Long^3^, Fan Wu^1^, Xuanyi Lu^2^, Yu Liu^1^, Ruiting Sun^1^, Xinqing Lin^1^, Xiaohong Xie^1^, Yumin Zhou^1,2^, Chengzhi Zhou^1,2*^, Pixin Ran^1,2*^

Affiliations

^1^State Key Laboratory of Respiratory Diseases, National Clinical Research Center for Respiratory Disease, National Center for Respiratory Medicine, Guangzhou Institute of Respiratory Health, the First Affiliated Hospital of Guangzhou Medical University, Guangzhou, Guangdong, China.

^2^Guangzhou National Laboratory, Guangzhou, Guangdong, China.

^3^State Key Laboratory of Respiratory Health and Multimorbidity, Institute of Basic Medical Sciences, Chinese Academy of Medical Sciences and Peking Union Medical College, Beijing, China

^#^Erkang Yi, Qingyang Li and Wenqian Wu contributed equally to this paper.

^*^Correspondence to Chengzhi Zhou or Pixin Ran

Email address: pxran@gzhmu.edu.cn, State Key Laboratory of Respiratory Diseases, National Clinical Research Center for Respiratory Diseases, the First Affiliated Hospital of Guangzhou Medical University, 195 Dongfeng Xi Road, Guangzhou, Guangdong 510182, China.

Supplementary Method and Materials

**Statistical analysis for NHANES**

The National Health and Nutrition Examination Survey (NHANES) study utilizes a sophisticated, multistage probability sampling design to select a representative sample of the U.S. population. Given the incorporation of multiple NHANES survey cycles into this analysis, a revised sampling weight was calculated to accurately reflect the combined dataset. This methodological adjustment ensures that all statistical analyses appropriately account for the complex survey design and weight variables, thereby preserving the integrity of the NHANES sampling framework.

Demographic characteristics, anthropometric measurements, smoking status, and diagnoses of chronic bronchitis/emphysema and chronic obstructive pulmonary disease (COPD) were statistically described for the total sample, as well as for lung cancer and non-lung cancer subgroups. To account for potential confounders, analyses were adjusted for age (both categorically and continuously), gender, Body Mass Index (BMI), smoking status, and race/ethnicity. Additionally, weights were applied to the data to enable pooling across survey waves.

Four multivariable linear regression models were constructed. Model 1 was a crude model for participants aged 20-80 years without adjusting for any covariates. Model 2 expanded upon Model 1 by adjusting for age, sex, race, smoking status, and BMI (kg/m²). A separate set of models was developed for participants aged 40-80 years: Model 3 was a crude model without adjustments, and Model 4 built upon Model 3 by incorporating adjustments for age, sex, race, smoking status, and BMI (kg/m²).

To assess the robustness of the associations between chronic bronchitis, emphysema, COPD, and lung cancer, sensitivity analyses were conducted using propensity score matching (PSM). Three separate PSM models were estimated, each comparing a disease group (chronic bronchitis, emphysema, or COPD) to a no-disease control group, matched on sex, age, race, smoking status, and BMI. In each model, participants were matched 1:1 based on their propensity score, with a caliper width of 0.01 to ensure precise matching.

Weighted multivariate linear regression was performed on propensity score-matched data to assess the persistence of the association between chronic bronchitis, emphysema, COPD, and lung cancer after controlling for potential confounders. A crude model was initially constructed, followed by a second model adjusted for age, sex, race, smoking status, and BMI (kg/m²).

Odds ratios (OR) were calculated for three distinct models: Model 1 included all participants aged 20-80 years, Model 2 included participants aged 40-80 years, and Model 3 included participants aged 40-80 years following propensity score matching.

Analytical models incorporated chronic bronchitis, emphysema, and COPD status (categorical variables) alongside pulmonary function metrics (continuous) as predictors of lung cancer outcomes (binary). Multivariable logistic regression with covariate adjustment (age, sex, BMI, race, smoking status) was performed for primary analysis, supplemented by Firth's bias-reduced logistic regression in propensity score-matched cohorts. All analyses were conducted using R 4.2.2 and SPSS 27.0, with statistical significance thresholded at *p*<0.05.

**Genetic correlation of lung cancer with COPD, emphysema/chronic bronchitis, and lung function parameters using LD score regression (LDSC) and high-definition likelihood (HDL)**

To estimate the shared heritability of lung cancer, LUAD, LUSC, and SCLC with COPD, emphysema/chronic bronchitis, and lung function parameters, we first performed cross-trait linkage disequilibrium (LD) score regression^1^ (LDSC, <https://github.com/bulik/ldsc/wiki/Heritability-and-Genetic-Correlation>) based on GWAS summary statistics. This analysis aimed to estimate the genetic correlation between lung cancer (including LUAD, LUSC, and SCLC), COPD, emphysema, chronic bronchitis, and lung function parameters. Utilizing LDSC with precomputed LD scores derived from approximately 1.2 million common and well-imputed HapMap3^2^ SNPs within European populations (excluding the HLA region) and anchored in the HapMap3 reference panel, genetic correlation estimates were generated on a scale of -1 to 1, with statistical significance defined as *p < 0.05*. Despite suggestions of cross-trait LDSC's resilience to sample overlap bias, a sensitivity analysis employing GWAS summary-level data from non-overlapping FinnGen cohorts was conducted to rigorously assess and mitigate any potential influence of sample overlap on the estimated genetic correlations between lung cancer, COPD, emphysema, chronic bronchitis, and lung function.

To estimate genetic correlations for COPD, emphysema, chronic bronchitis, lung function, and lung cancer, we employed the HDL method, a recent extension of bivariate LDSC that capitalizes on LD information across the entire autosomal genome^3^, excluding the MHC region (chromosome 6: 28,477,797-33,448,354 bp), using GWAS summary statistics. By incorporating an additional variance-covariance LD matrix, HDL significantly reduces the variance of genetic correlation (rg) estimates, thereby enhancing power compared to bivariate LDSC. The analysis was conducted using the default 1000G reference panel with 1 million imputed HapMap3 autosomal SNPs, after excluding ambiguous SNPs.

**Multi-trait analysis of GWAS**

To identify risk SNPs associated with joint phenotypes of COPD and lung cancer, we conducted a cross-trait meta-analysis of GWAS summary statistics using the MTAG tool (Python 2.7)^4^. We selected MTAG over traditional inverse-variance weighted meta-analyses with trait-specific effect sizes due to its ability to handle potential sample overlap between GWAS datasets. MTAG was configured under the assumption of equal SNP heritability across traits and perfect genetic covariance between them. To further investigate whether deviations from the assumptions of equal SNP heritability and perfect genetic covariance might bias our MTAG results, we performed a sensitivity analysis using cross-phenotype associationtest (CPASSOC)^5^. CPASSOC accounts for heterogeneous effects across traits and estimates the cross-trait statistic SHet and p-value through a sample size-weighted meta-analysis of GWAS summary data. We prioritized independent SNPs that reached genome-wide significance in both the MTAG and CPASSOC cross-trait meta-analyses. These genome-wide significant SNPs were identified using LD clumping (r² < 0.001 within 10,000-kb windows) in PLINK v1.970, based on the UK Biobank European reference panel imputed with the Haplotype Reference Consortium (HRC).

**MR Analysis**

**Data source**

This study employed MR analyses using R (version 4.2.3) ^6^ with the TwoSampleMR package. We investigated the causal effects of exposures including COPD, emphysema/chronic bronchitis, and lung function parameters on outcomes like lung cancer, LUAD, LUSC, and SCLC. Summary-level data for *IREB2* expression quantitative trait loci (eQTLs) was obtained from the eQTLGen consortium (<https://eqtlgen.org/>)^7^, encompassing blood-derived data from 31,684 participants (predominantly European ancestry) and lung tissue data from 515 samples (mostly European ancestry) from the GTEx project^8^. We identified eQTL genes influenced by SNP loci within the eQTLGen database. Protein quantitative trait locus (pQTL) data for IREB2 was sourced from the UK Biobank Pharma Proteomics Project (UKB-PPP)^9^, which includes protein measurements in 54,219 UK Biobank participants and has identified 14,287 primary genetic associations across 2,923 proteins.

**Selection of Instrumental Variables (IVs)**

Single nucleotide variants (SNVs) were selected as instrumental variables (IVs) for COPD, FEV1, FVC, lung cancer, LUAD, LUSC, and SCLC from the exposure dataset if they were significantly associated with the exposure (*p < 5.0 × 10⁻⁸*) and were independent of other SNPs (r² < 0.001, distance > 10,000 kb). For emphysema/chronic bronchitis (ukb-a-7280), IVs were selected from the IEU GWAS (*p < 5.0 × 10*⁻⁵) and FinnGen GWAS (*p < 1.0 × 10*⁻⁵). Additionally, SNVs from the GTEx project were chosen as IVs if significantly associated with the exposure (*p < 1.0 × 10⁻⁶*) and meeting the aforementioned LD criteria. IVs with an F-statistic below 10 were discarded as weak instruments. A reverse MR analysis was subsequently performed, treating lung cancer, LUAD, LUSC, and SCLC as exposures and COPD, emphysema/chronic bronchitis, and lung function parameters as outcomes, using the same methodology.

The exposure instruments for 731 immune cells were derived from the research conducted by Valeria Orrù and colleagues^10^, which investigated the influence of approximately 22 million genetic variants on 731 immune cell traits in a cohort of 3,757 Sardinians; variants were selected for each cell subset based on a stringent threshold of *p < 5.0 × 10*⁻⁶ and were required to be independent (r² < 0.001 and distance > 10,000 kb).

**Statistical Analysis**

For the MR analysis, the Wald ratio was utilized for proteins with a single genetic instrument, while the inverse variance weighted MR (MR-IVW) method, accompanied by heterogeneity analysis, was employed for proteins with multiple applicable instruments. Additionally, Bayesian Weighted Mendelian Randomization (BWMR)^11^ was incorporated to validate result sensitivity. To assess the robustness of findings, several sensitivity analyses were conducted: the Weighted Median Method for handling up to 50% invalid instruments, the Weighted Mode Method for identifying the mode of causal effects distribution, and the MR-Egger Intercept for evaluating horizontal pleiotropy, with a *p-value > 0.05* indicating minimal pleiotropy. Cochran’s Q Statistic was used to detect heterogeneity in IV associations, with a *p > 0.05* signifying non-heterogeneity and prompting a fixed-effects model, while *p < 0.05* indicated heterogeneity and necessitated a random-effects model. MR-PRESSO Global Test was employed to detect and correct for horizontal pleiotropy by identifying and adjusting outliers when significant (*p < 0.05*). Finally, Leave-One-Out Analysis assessed the influence of individual SNPs on the overall estimate, ensuring results were not driven by a single SNP.

**Meta-Analysis**

A meta-analysis was performed using METAL software (<https://github.com/statgen/METAL>). Based on heterogeneity evaluation, we implemented either a fixed-effects model or an inverse-variance weighted random-effects model.

**Mediation MR Analysis**

We conducted a two-step MR (<https://mr-dictionary.mrcieu.ac.uk/term/two-step/>)^12^ analysis to evaluate mediation within an MR framework. In the first step, we investigated the associations between the exposure and the outcome, the exposure and mediators, and the mediators and the outcome using univariable MR. Specifically, we employed IVW MR to assess the association between “Ever smoked” and COPD (exposure to mediators), as well as the association between COPD and the liability to ever having smoked with lung cancer, LUAD, LUSC, and SCLC (mediators to outcome). Moreover, we utilized IVW MR to assess the association between COPD and the eQTLs of *IREB2* (exposure to mediators), as well as the association between the eQTLs of *IREB2* and the liability to COPD with lung cancer, LUAD, LUSC, and SCLC (mediators to outcome). Finally, we employed IVW MR to assess the association between COPD and *CD27* on B cells (exposure to mediators), as well as the association between *CD27* on B cells and the liability to COPD with lung cancer (mediators to outcome).

**Summary-data-based Mendelian (SMR) randomization analyses**

We employed SMR and Heterogeneity in Dependent Instruments (HEIDI) tests within cis-regulatory regions using the SMR software (<https://yanglab.westlake.edu.cn/software/smr/#Overview>)^13^. This methodology, based on the original SMR publication, leverages a single nucleotide variant (SNV) at a primary cis-eQTL as an IV. This IV, in conjunction with summary-level eQTL, pQTL, and GWAS data, facilitated the investigation of potential causal or pleiotropic associations between gene expression and traits of interest. While the SMR approach, due to its reliance on a single IV, cannot definitively distinguish between causal and pleiotropic associations, the HEIDI test provides an additional layer of analysis to differentiate causality from linkage. We further extended the SMR and HEIDI analyses by utilizing summary-level sQTL data as exposures. Standard settings within the SMR software were maintained, including a p-value threshold of 5.0 × 10⁻⁸ for selecting top eQTLs and a 2 Mb window around the probe center for cis-eQTL selection. Consistent with the use of a single probe, SMR analyses were confined to cis regions and statistical significance was set at a p-value ≤ 0.05. For HEIDI, a *p*-value < 0.05 indicated the presence of significant linkage, suggesting the observed association was not purely causal.

**Colocalization Analysis**

COLOC analysis (<https://chr1swallace.github.io/coloc/index.html>)^14^ assesses shared causal variants for gene expression and phenotypes at the same locus. It utilizes a Bayesian framework to evaluate five hypotheses: no causal variant (H_0_), a causal variant specific to trait 1 (H_1_), a causal variant specific to trait 2 (H_2_), distinct causal variants for each trait (H_3_), and a shared causal variant for both traits (H_4_). Using the 'coloc' R package, we estimated the posterior probability (PP) of shared causal variants (H_4_) for the association between COPD, emphysema/chronic bronchitis, and lung cancer subtypes (LUAD, LUSC, and SCLC). We specifically focused on H_3_ and H_4_, considering a PP score above 0.70 as evidence for shared causal variants influencing both gene expression and phenotype. Notably, we only analyzed regions within 1 Mb flanking the lead GWAS variant with the most significant *p*-value.

**TWAS and Phenome-wide association studies (PheWAS)**

To identify genes potentially involved in lung cancer, LUAD, LUSC, SCLC, COPD, and emphysema/chronic bronchitis, TWAS (<http://gusevlab.org/projects/fusion/#installation>)^15^ was conducted, leveraging gene expression data from GTEx v.8 lung tissues and whole blood, along with GWAS summary statistics for the aforementioned traits. By comparing gene-based models of genetic effects on gene expression, TWAS estimated the strength of the association between concordant genetic influences and the traits of interest. Bonferroni correction was applied to account for multiple comparisons across the substantial number of gene-tissue pairs tested (approximately 200,000). Significant expression-trait associations were defined as *p < 0.05*. This approach identified genes with significant associations for lung cancer, lung function parameters, or respiratory diseases. Additionally, to identify an independent set of gene-based genetic models, we employed an extension of TWAS that incorporates conditional tests of SNPs using summary statistics from the PheWAS Web (<https://pheweb.org/UKB-TOPMed/>).

**Gene Expression, Prognostic Analysis, and Enrichment Analysis**

We utilized publicly available data from several sources for our analysis. Normalized pan-cancer datasets The Cancer Genome Atlas (TCGA) ^16^ were downloaded from the UCSC Xena browser (<https://xenabrowser.net>) for LUAD, LUSC, and control samples. Expression data for *IREB2* and *PSMA4* genes were extracted and transformed using a log^2(x+0.001)^ function. Moreover, lung tissue sequencing data for COPD patients (GSE76925) ^17^ were obtained from the Gene Expression Omnibus (GEO) database, normalized, and used to extract *IREB2* and *PSMA4* expression levels. Finally, immunohistochemistry (IHC) images of *IREB2* in LUAD and LUSC tissues, along with adjacent normal tissues, were retrieved from the Human Protein Atlas (HPA) website (<https://www.proteinatlas.org/>)^18^.

To assess the prognostic significance of IREB2 and PSMA4 RNA expression levels in lung cancer subtypes, LUAD and LUSC, we utilized the Kaplan-Meier plotter (KMplot) database^19^. Protein level data and prognostic analysis for IREB2 and PSMA4 in LUAD and LUSC were obtained from the Protein Atlas (PCAS) website (<https://jingle.shinyapps.io/PCAS/>)^20^. Additionally, mutation analysis of *IREB2* in LUAD and LUSC was performed using the *cBioPortal* database (<https://www.cbioportal.org/>).

To investigate functional enrichment, overlapping genes were uploaded to Metascape (<https://metascape.org/gp/index.html>)^21^ for Gene Ontology (GO) analysis. We then conducted a Gene Set Enrichment Analysis (GSEA)^22^ on LUAD and LUSC expression matrices. Samples were divided into high and low *IREB2* expression groups based on the median expression level. Subsequently, pathway enrichment analyses were performed using KEGG^23^ and Reactome^24^ databases for both groups. Likewise, KEGG and Reactome enrichment analyses were applied to lung tissue sequencing data from COPD patients following the same criteria.

**Immune Infiltration Analysis**

Pearson's correlation coefficients were calculated between *IREB2* gene expression and immune infiltration scores in LUAD and LUSC samples. The R package 'psych' (version 2.1.6) and its 'corr.test' function were used for this analysis. Immune infiltration scores included ESTIMATE (<https://bioinformatics.mdanderson.org/estimate/>), CIBERSORT (<https://cibersortx.stanford.edu/>)^25^, EPIC (<http://epic.gfellerlab.org>)^26^, MCPcounter (<https://github.com/ebecht/MCPcounter>)^27^, and xCELL (<https://github.com/dviraran/xCell>)^28^. We further assessed the correlation between *IREB2* and immune regulatory genes, immune checkpoints, and RNA modification genes to identify statistically significant associations between IREB2 expression and immune infiltration.

To investigate the relationship between IREB2 expression and immune cell infiltration, the TIMER2.0 database (<http://timer.cistrome.org/>)^29^ was used to analyze correlations between IREB2 and various immune cell subtypes. Additionally, the GEPIA2 database (<http://gepia2.cancer-pku.cn/>)^30^ was employed to evaluate the correlation between IREB2 expression and markers of memory B cells specifically within LUAD and LUSC.

**Sample collection, RNA extraction, cDNA synthesis, and qRT-qPCR**

Peripheral blood specimens from 49 control subjects and 55 COPD patients enrolled in the Early COPD (ECOPD) trial (Chinese Clinical Trial Registry: ChiCTR1900024643)^31^ were randomly selected for inclusion. Subsets of this cohort have been reported in earlier publications. Venous blood collected by certified personnel using EDTA-coated tubes was centrifuged within 2 hours and cryopreserved at −80°C for downstream analysis. RNA isolation from blood was conducted with Trizol Reagent (Invitrogen) following standardized procedures^32^. Reverse transcription employing the Evo M-MLV RT Kit (AG, China) converted 1 μg RNA templates into cDNA, with genomic DNA elimination per manufacturer guidelines. Quantitative amplification was implemented through SYBR Green-based chemistry (Pro Taq HS Kit, AG) using a Bio-Rad CFX Connect thermocycler. Relative quantification against the GAPDH endogenous reference was calculated via the comparative threshold cycle (2-^ΔΔCT^) approach. Primer sequences are detailed in Supplementary Table 12.

**Immunofluorescence (IF) assay**

Immunofluorescence (IF) staining was conducted following standard protocols^33^. Lung cancer tissue microarray underwent incubation with primary antibodies targeting IREB2 (A6382, Abclonal), CD19 (A19013, Abclonal), and CD27 (A11505, Abclonal) for 2 hours at 37°C. Sections were subsequently probed with fluorescent secondary antibodies: Alexa Fluor 488-conjugated goat anti-rabbit IgG (H+L) (1:500, Invitrogen) and Alexa Fluor 594-conjugated rabbit anti-mouse IgG (H+L) (1:500, Invitrogen) for 40 minutes. Images were acquired using a Leica DM6 M microscope system.

**Single-cell sequencing and analysis of COPD and lung cancer**

Two publicly available single-cell RNA-sequencing (scRNA-seq) datasets were utilized for this study. The first dataset^34^, accessible at <https://gbiomed.kuleuven.be/english/cme/research/laboratories/54213024/scRNAseq-NSCLC>, includes 10 samples encompassing cancerous and adjacent non-cancerous tissues from 5 lung cancer patients. This dataset comprises a total of 51,780 cells and 24,075 genes. The second dataset, obtained from the GEO database (dataset GSE173896)^35^focuses on COPD. It encompasses 16 samples across three groups: 9 COPD patients, 4 non-COPD smokers, and 3 never-smokers. This dataset comprises a total of 52,764 cells and 33,538 genes. Integrated Pan-Cancer B cell dataset analysis and visualization were generated from the Cirrocumulus database (<http://pan-b.cancer-pku.cn/>)^36^. Established cell type-specific marker genes were used for analysis. The R environment and Seurat package (<https://satijalab.org/seurat/>)^37^ were employed for single-cell RNA-seq data analysis and visualization. The analysis pipeline involved data normalization, dimensionality reduction, and clustering, following established protocols. Differentially expressed genes (DEGs) across cell subsets were identified and subjected to enrichment analysis using Metascape. Intercellular communication was analyzed using the "CellChat" R package (version 1.1.3). Pseudotime analysis was validated using the "Monocle 2" R package. Visualization of single-cell analysis results was achieved using the R package "ggplot2". Batch effect correction was performed on isolated B cells and plasma cells in scRNA-seq data using "Harmony" from integration. Prognostic analysis of cell subpopulations was conducted using the top 20 marker genes per subcluster from scRNA-seq data, implemented via the KMplot database^19^.

**Single-cell phenotype-associated subpopulation (scPAS)**

The scPAS framework^38^ systematically identified phenotype-associated subpopulations in human scRNA-seq data. Gene-gene interaction networks were constructed using Pearson correlation (|r| > 0.25) and shared nearest neighbor (k = 20) algorithms. A network-regularized sparse regression (NRSR) model trained *using* single-cell data jointly implemented L1-penalized feature selection (α = 0.1) and topology-aware regularization to align coefficients with network connectivity. The regularization parameter λ was optimized to 0.05 through tenfold cross-validation. Phenotype association scores (PAS) were computed for each cell, followed by permutation testing (500 iterations) to compute normalized PAS (|NPAS| > 1.96, *p* < 0.05). scPAS⁺/scPAS⁻ cells were subsequently classified, with pulmonary fibroblasts exhibiting transcriptomic signatures either concordant with or inversely correlated to sequencing-derived profiles.

**Cell culture, siRNA transfection and stimulation**

The human B-lymphoma cell line SU-DHL-4 (Procell Life Science, China) was cultured in RPMI 1640 medium (Gibco, USA) supplemented with 10% fetal bovine serum and 1% penicillin-streptomycin at 37°C in 5% CO₂. For siRNA transfection, cells were centrifuged at 1,200 rpm for 3 min, quantified, and seeded in 6-well plates at 3 × 10⁵ cells/well. Transfection complexes containing si-NC or si-IREB2 (Hiyobiotechnology, China) were prepared using Lipofectamine 3000 (Thermo Fisher Scientific, USA) according to the manufacturer’s protocol, gently mixed with cells, and incubated. At 24 h post-transfection, experimental groups were stimulated with 0.5% cigarette smoke extract in RPMI 1640 medium while controls received phosphate-buffered saline (PBS) at equivalent concentration; subsequent analyses were performed after 48 h of stimulation.

**Western blot (WB)**

Western blotting was performed following standard procedures. In brief, cells or lung tissue samples were homogenized in RIPA buffer (89901, Thermo) containing a protease inhibitor cocktail (78430, Thermo) and incubated on ice for 20 minutes. Equal amounts of protein were resolved on 10% SDS–polyacrylamide gels and subsequently transferred onto polyvinylidene difluoride (PVDF) membranes (Bio-Rad, USA). After blocking, membranes were incubated overnight at 4 °C with primary antibodies against CD19 (A19013, Abclonal), CD27 (A1945, Abclonal), HLA-DBP1 (A25794, Abclonal), and GAPDH (10494-1-AP, Proteintech). Following several washes, membranes were exposed to horseradish peroxidase–conjugated secondary antibodies (Proteintech). Protein signals were detected by enhanced chemiluminescence and imaged using an Amersham Imager 680 system (Thermo Fisher Scientific).

**References**

1. Bulik-Sullivan BK, Loh PR, Finucane HK, et al. LD Score regression distinguishes confounding from polygenicity in genome-wide association studies. *Nat Genet*. 2015;47(3):291-5.

2. Duan S, Zhang W, Cox NJ, Dolan ME. FstSNP-HapMap3: a database of SNPs with high population differentiation for HapMap3. *Bioinformation*. 2008;3(3):139-41.

3. Ning Z, Pawitan Y, Shen X. High-definition likelihood inference of genetic correlations across human complex traits. *Nat Genet*. 2020;52(8):859-864.

4. Turley P, Walters RK, Maghzian O, et al. Multi-trait analysis of genome-wide association summary statistics using MTAG. *Nat Genet*. 2018;50(2):229-237.

5. Li X, Zhu X. Cross-Phenotype Association Analysis Using Summary Statistics from GWAS. *Methods Mol Biol*. 2017;1666:455-467.

6. Walker VM, Davies NM, Hemani G, et al. Using the MR-Base platform to investigate risk factors and drug targets for thousands of phenotypes. *Wellcome Open Res*. 2019;4:113.

7. Vosa U, Claringbould A, Westra HJ, et al. Large-scale cis- and trans-eQTL analyses identify thousands of genetic loci and polygenic scores that regulate blood gene expression. *Nat Genet*. 2021;53(9):1300-1310.

8. Consortium GT. Human genomics. The Genotype-Tissue Expression (GTEx) pilot analysis: multitissue gene regulation in humans. *Science*. 2015;348(6235):648-60.

9. Sun BB, Chiou J, Traylor M, et al. Plasma proteomic associations with genetics and health in the UK Biobank. *Nature*. 2023;622(7982):329-338.

10. Orru V, Steri M, Sidore C, et al. Complex genetic signatures in immune cells underlie autoimmunity and inform therapy. *Nat Genet*. 2020;52(10):1036-1045.

11. Zhao J, Ming J, Hu X, Chen G, Liu J, Yang C. Bayesian weighted Mendelian randomization for causal inference based on summary statistics. *Bioinformatics*. 2020;36(5):1501-1508.

12. Xu L, Borges MC, Hemani G, Lawlor DA. The role of glycaemic and lipid risk factors in mediating the effect of BMI on coronary heart disease: a two-step, two-sample Mendelian randomisation study. *Diabetologia*. 2017;60(11):2210-2220.

13. Zhu Z, Zhang F, Hu H, et al. Integration of summary data from GWAS and eQTL studies predicts complex trait gene targets. *Nat Genet*. 2016;48(5):481-7.

14. Wallace C. A more accurate method for colocalisation analysis allowing for multiple causal variants. *PLoS Genet*. 2021;17(9):e1009440.

15. Gusev A, Ko A, Shi H, et al. Integrative approaches for large-scale transcriptome-wide association studies. *Nat Genet*. 2016;48(3):245-52.

16. Tomczak K, Czerwinska P, Wiznerowicz M. The Cancer Genome Atlas (TCGA): an immeasurable source of knowledge. *Contemp Oncol (Pozn)*. 2015;19(1A):A68-77.

17. Morrow JD, Zhou X, Lao T, et al. Functional interactors of three genome-wide association study genes are differentially expressed in severe chronic obstructive pulmonary disease lung tissue. *Sci Rep*. 2017;7:44232.

18. Thul PJ, Akesson L, Wiking M, et al. A subcellular map of the human proteome. *Science*. 2017;356(6340)

19. Lanczky A, Gyorffy B. Web-Based Survival Analysis Tool Tailored for Medical Research (KMplot): Development and Implementation. *J Med Internet Res*. 2021;23(7):e27633.

20. Wang J, Song X, Wei M, et al. PCAS: An Integrated Tool for Multi-Dimensional Cancer Research Utilizing Clinical Proteomic Tumor Analysis Consortium Data. *Int J Mol Sci*. 2024;25(12)

21. Zhou Y, Zhou B, Pache L, et al. Metascape provides a biologist-oriented resource for the analysis of systems-level datasets. *Nat Commun*. 2019;10(1):1523.

22. Subramanian A, Tamayo P, Mootha VK, et al. Gene set enrichment analysis: a knowledge-based approach for interpreting genome-wide expression profiles. *Proc Natl Acad Sci U S A*. 2005;102(43):15545-50.

23. Kanehisa M, Furumichi M, Sato Y, Kawashima M, Ishiguro-Watanabe M. KEGG for taxonomy-based analysis of pathways and genomes. *Nucleic Acids Res*. 2023;51(D1):D587-D592.

24. Rothfels K, Milacic M, Matthews L, et al. Using the Reactome Database. *Curr Protoc*. 2023;3(4):e722.

25. Rusk N. Expanded CIBERSORTx. *Nat Methods*. 2019;16(7):577.

26. Racle J, Gfeller D. EPIC: A Tool to Estimate the Proportions of Different Cell Types from Bulk Gene Expression Data. *Methods Mol Biol*. 2020;2120:233-248.

27. Becht E, Giraldo NA, Lacroix L, et al. Estimating the population abundance of tissue-infiltrating immune and stromal cell populations using gene expression. *Genome Biol*. 2016;17(1):218.

28. Aran D. Cell-Type Enrichment Analysis of Bulk Transcriptomes Using xCell. *Methods Mol Biol*. 2020;2120:263-276.

29. Li T, Fu J, Zeng Z, et al. TIMER2.0 for analysis of tumor-infiltrating immune cells. *Nucleic Acids Res*. 2020;48(W1):W509-W514.

30. Tang Z, Kang B, Li C, Chen T, Zhang Z. GEPIA2: an enhanced web server for large-scale expression profiling and interactive analysis. *Nucleic Acids Res*. 2019;47(W1):W556-W560.

31. Wu F, Zhou Y, Peng J, et al. Rationale and design of the Early Chronic Obstructive Pulmonary Disease (ECOPD) study in Guangdong, China: a prospective observational cohort study. *J Thorac Dis*. 2021;13(12):6924-6935.

32. Yi E, Wang X, Liu Y, et al. Long Noncoding RNA Interleukin 6 Antisense RNA 1 Promotes Inflammatory Effects in Lung Macrophages via Exosomes Through the S100A9/TLR4 Pathway in Chronic Obstructive Pulmonary Disease Progression. *MedComm (2020)*. 2025;6(6):e70204.

33. Yi E, Li H, Liu Y, et al. An integrated machine learning model of transcriptomic genes in multi-center chronic obstructive pulmonary disease reveals the causal role of TIMP4 in airway epithelial cell. *Respir Res*. 2025;26(1):158.

34. Prazanowska KH, Lim SB. An integrated single-cell transcriptomic dataset for non-small cell lung cancer. *Sci Data*. 2023;10(1):167.

35. Watanabe N, Fujita Y, Nakayama J, et al. Anomalous Epithelial Variations and Ectopic Inflammatory Response in Chronic Obstructive Pulmonary Disease. *Am J Respir Cell Mol Biol*. 2022;67(6):708-719.

36. Yang Y, Chen X, Pan J, et al. Pan-cancer single-cell dissection reveals phenotypically distinct B cell subtypes. *Cell*. 2024;187(17):4790-4811 e22.

37. Hao Y, Stuart T, Kowalski MH, et al. Dictionary learning for integrative, multimodal and scalable single-cell analysis. *Nat Biotechnol*. 2024;42(2):293-304.

38. Xie A, Wang H, Zhao J, Wang Z, Xu J, Xu Y. scPAS: single-cell phenotype-associated subpopulation identifier. *Brief Bioinform*. 2024;26(1)

Supplementary Figure


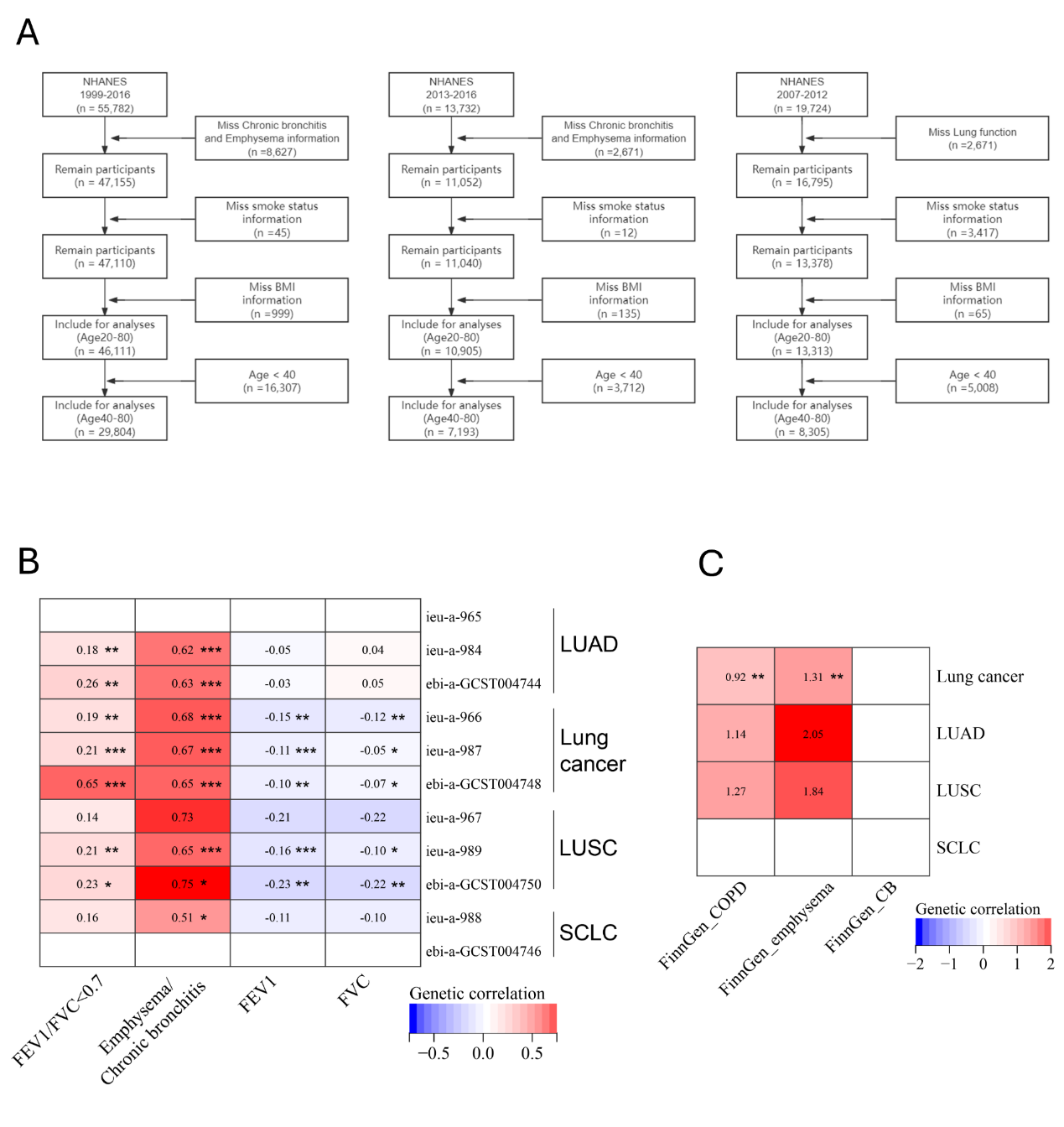


**Figure S1**

**A** Inclusion and exclusion process for the population analyzed in the NHANES database. The populations include those with chronic bronchitis and emphysema (1999-2016), physician-diagnosed COPD (2013-2016), and individuals with lung function data (2007-2012). Individuals without information on emphysema, chronic bronchitis, COPD, lung function, smoking status, or BMI were excluded. The final analysis included individuals aged 20-80 years. Subsequently, those aged 20-39 years were excluded, resulting in a final analysis cohort of individuals aged 40-80 years.

**B** Whole-genome genetic correlations of COPD, emphysema, chronic bronchitis, and lung function in IEU GWAS **(B)** and FinnGen GWAS **(C)** with lung cancer using HDL. Colors represent the magnitude of the genetic correlation of COPD, emphysema, chronic bronchitis, and lung function with lung cancer (lung cancer, LUAD, LUSC, and SCLC), using LDSC, with red indicating positive genetic correlation, blue indicating negative genetic correlation and white indicating low true heritability where results could not be calculated. Numbers represent the genetic correlation. * (*p<0.05*), ** (*p<0.005*) and *** (*p<0.0001*) represent significant genetic correlation. All *p* values are two-sided.


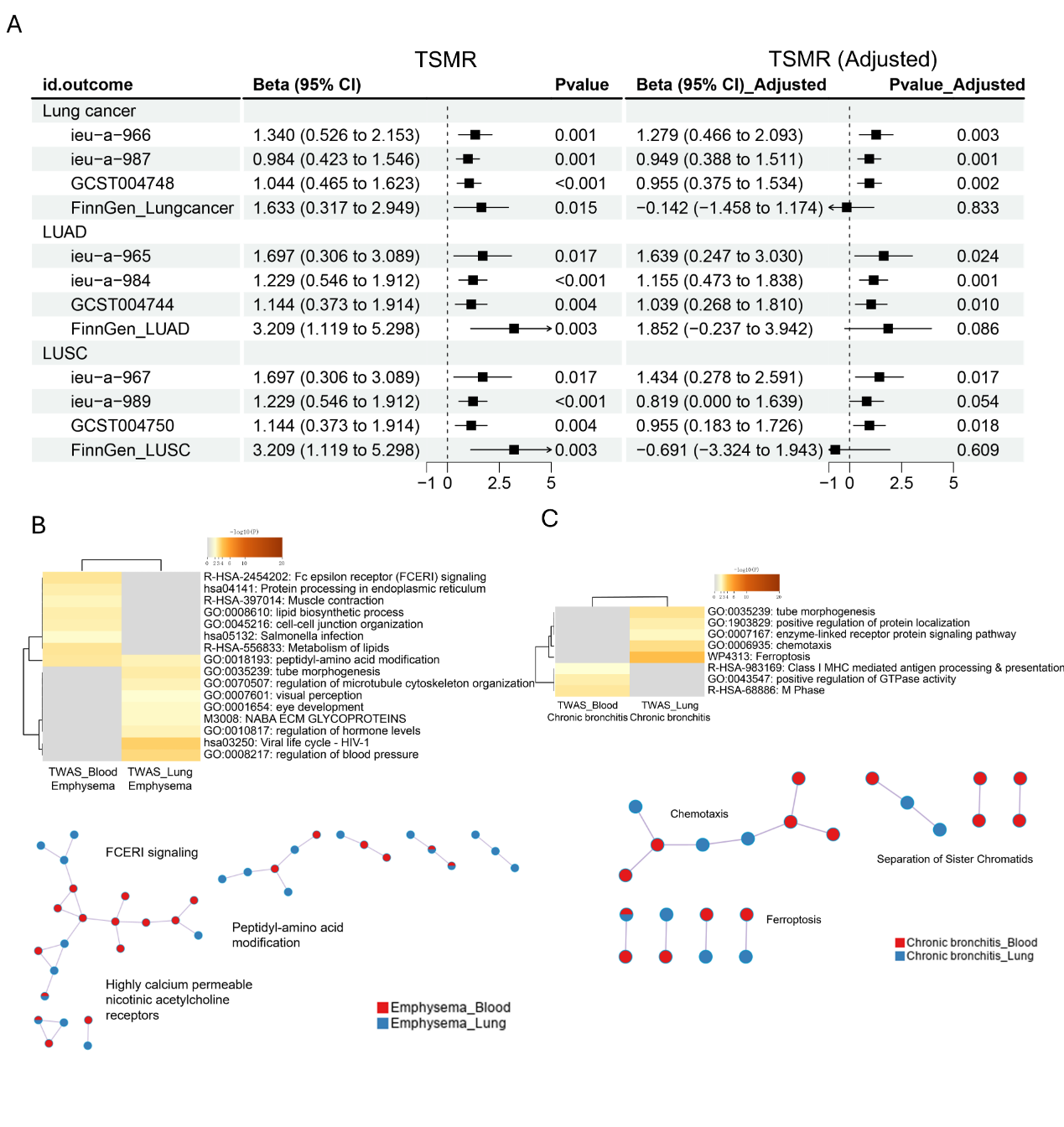


**Figure S2**

**A** Forest plot of Two-Sample MR analyses between Ever smoked and lung cancer including LUAD and LUSC before and after removing the mediator effect of COPD. Effect sizes (Beta, 95% CI) are shown as the standard deviation change in lung cancer per standard deviation increase in Ever smoked. Points on the forest plot represent effect size estimates, while whiskers denote 95% CIs. All *P values* are two-side.

**B** Enrichment and PPI network analysis of overlapping genes from TWAS of Emphysema and lung cancer in lung tissue and peripheral blood using Metascape.

**C** Enrichment and PPI network analysis of overlapping genes from TWAS of Chronic bronchitis and lung cancer in lung tissue and peripheral blood using Metascape.


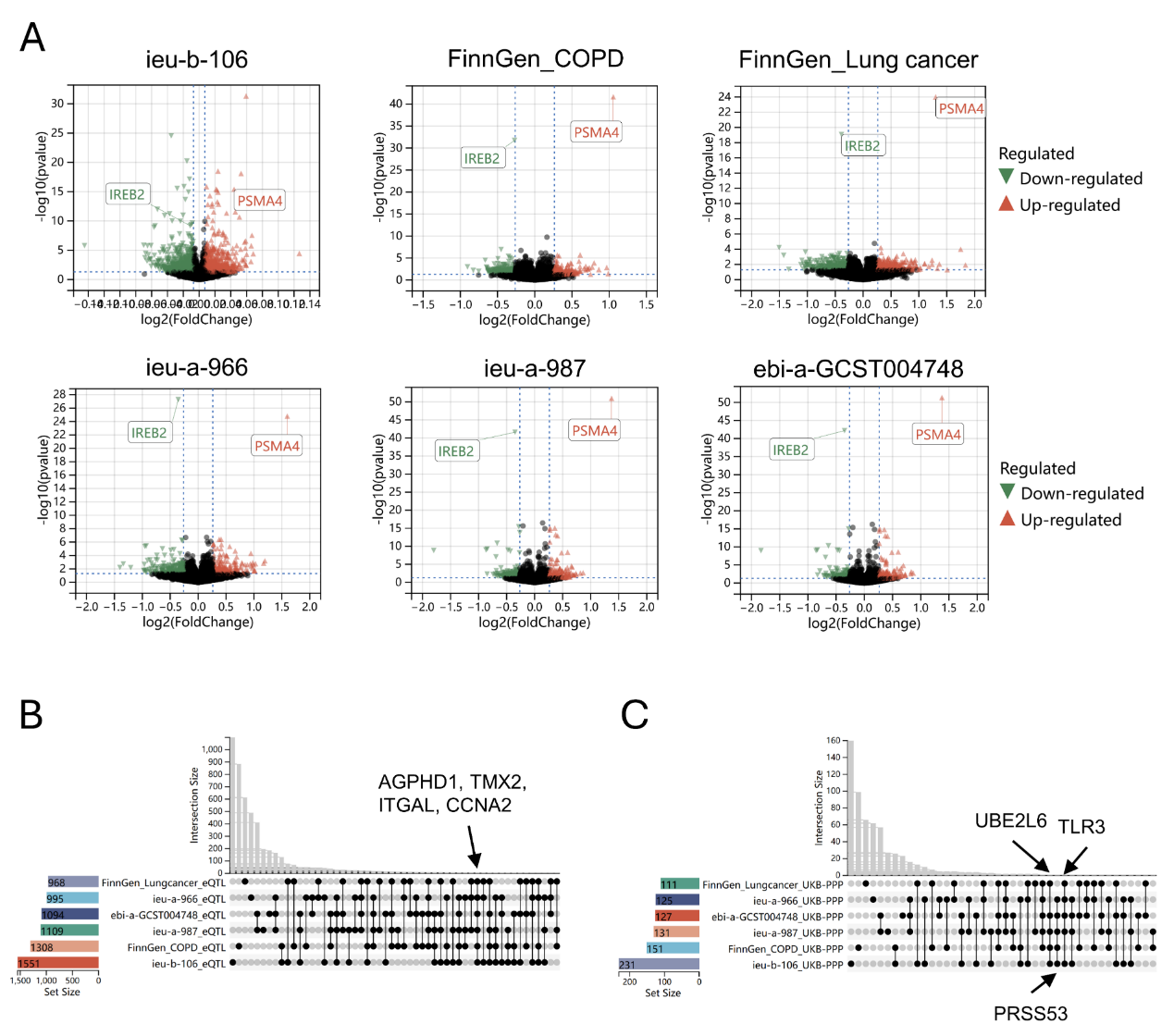


**Figure S3**

**A** Volcano plot illustrating the SMR results of COPD and lung cancer using eQTLGen database.

**B-C** Upset plot displaying the overlapping genes in the SMR results for COPD and lung cancer using eQTLGen (B) and UKB-PPP database (C).


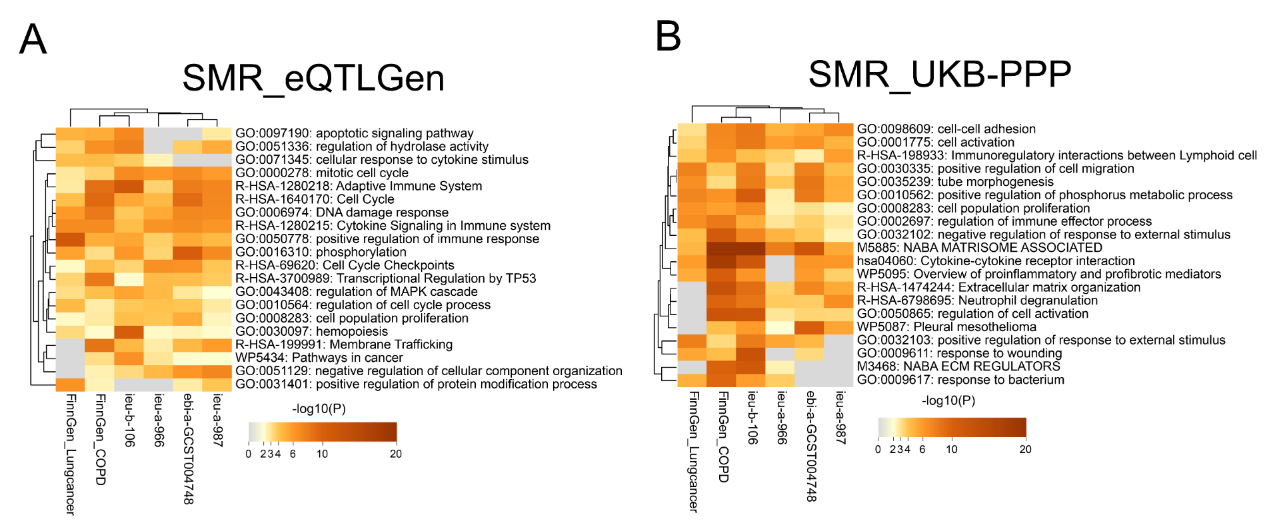


**Figure S4**

**A-B** Enrichment analysis results of SMR eQTLs for COPD and lung cancer, derived from eQTLGen (A), and pQTLs from UKB-PPP (B), using the Metascape database.


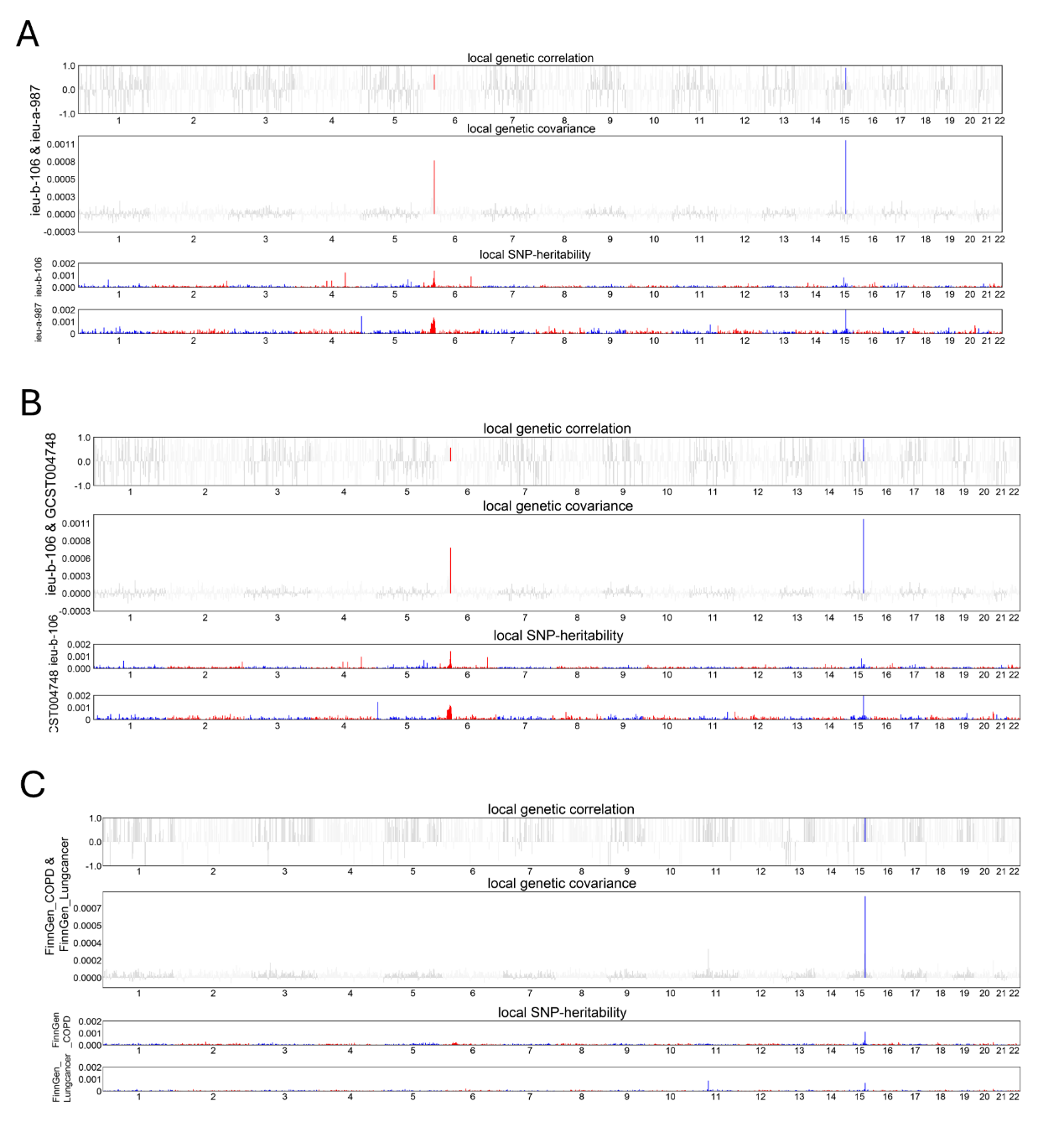


**Figure S5**

**A-C** The local heritability and genetic correlation between COPD and Lung Cancer including ieu-b-106 and ieu-a-987 (A), ieu-b-106 and ieu-a-GCST004748 (B) and FinnGEN_COPD and FinnGen lung cancer (C) across the genome. The top two panels display the local heritability estimates for COPD and lung cancer, where blue indicates statistically significant positive correlation and red indicates statistically significant negative correlation. The third panel shows local genetic covariance, with blue indicating positive covariance and red indicating negative covariance.


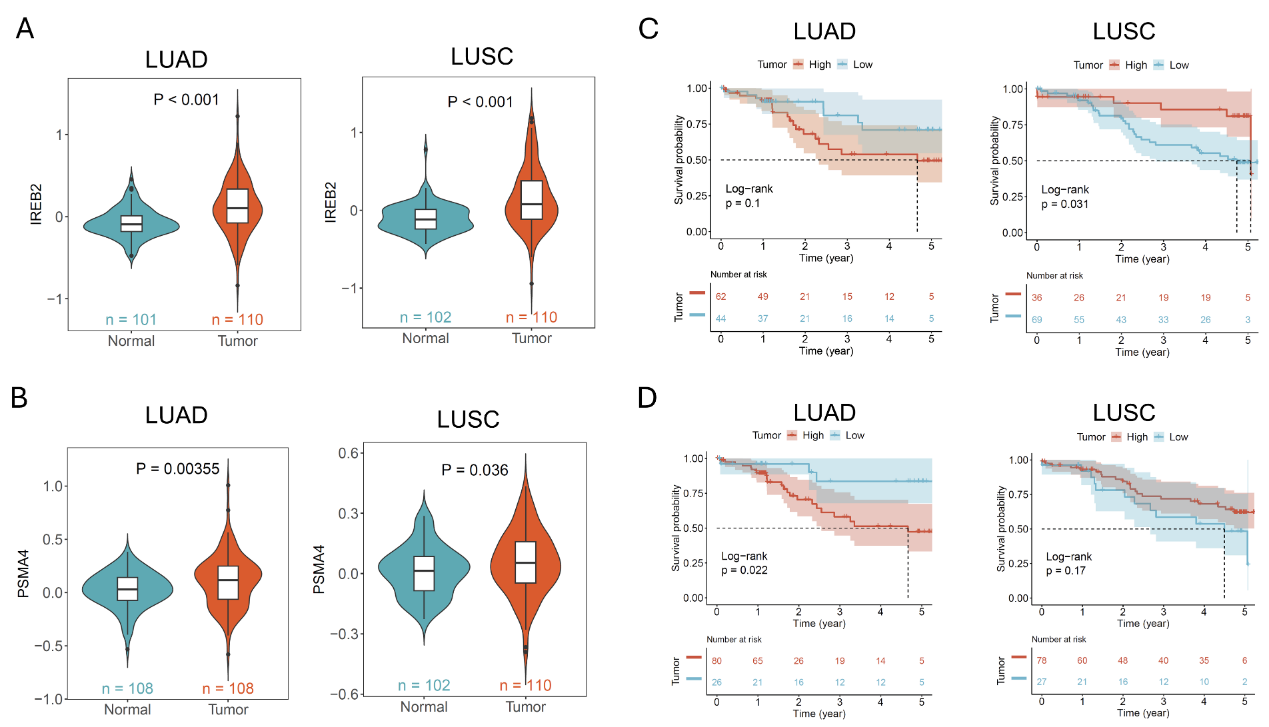


**Figure S6**

**A-D** Protein-level expression of IREB2 (A) and PSMA4 (B) in LUAD and LUSC, and its survival prognosis (C-D), utilizing data from the PCAS database.


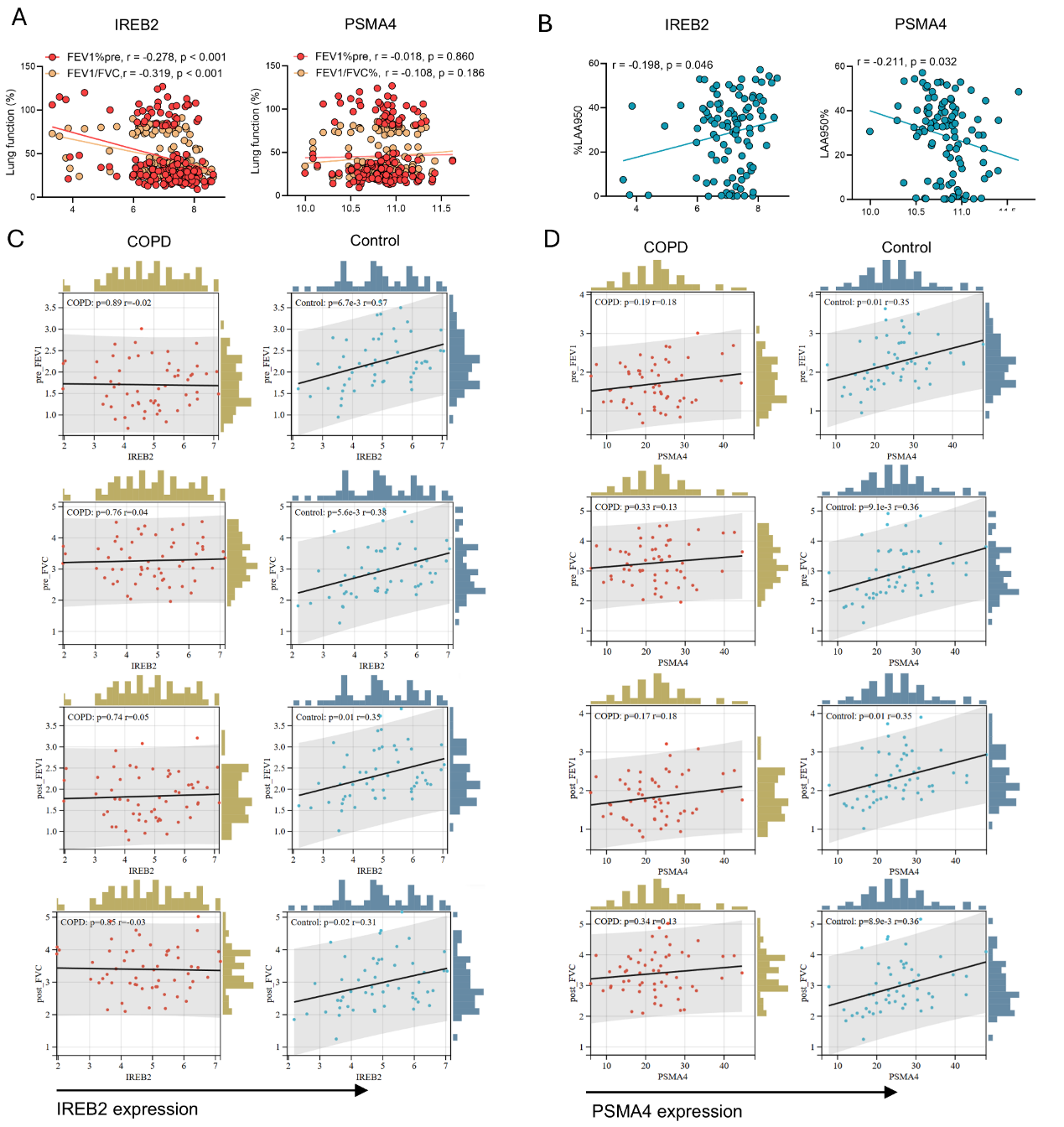


**Figure S7**

A-B Association analyses of IREB2 and PSMA4 expression in lung tissue with pulmonary function parameters (FEV₁%predicted and FEV₁/FVC%) (A) and the emphysema index (LAA₉₅₀%) (B) from the GSE76925 dataset. Pearsons *correlation* *analysis*.

**C** Association analyses of IREB2 and PSMA4 expression in peripheral blood with pre- and post-bronchodilator FEV₁ and FVC in control subjects and COPD patients from the ECOPD cohort. Pearsons *correlation* *analysis*.


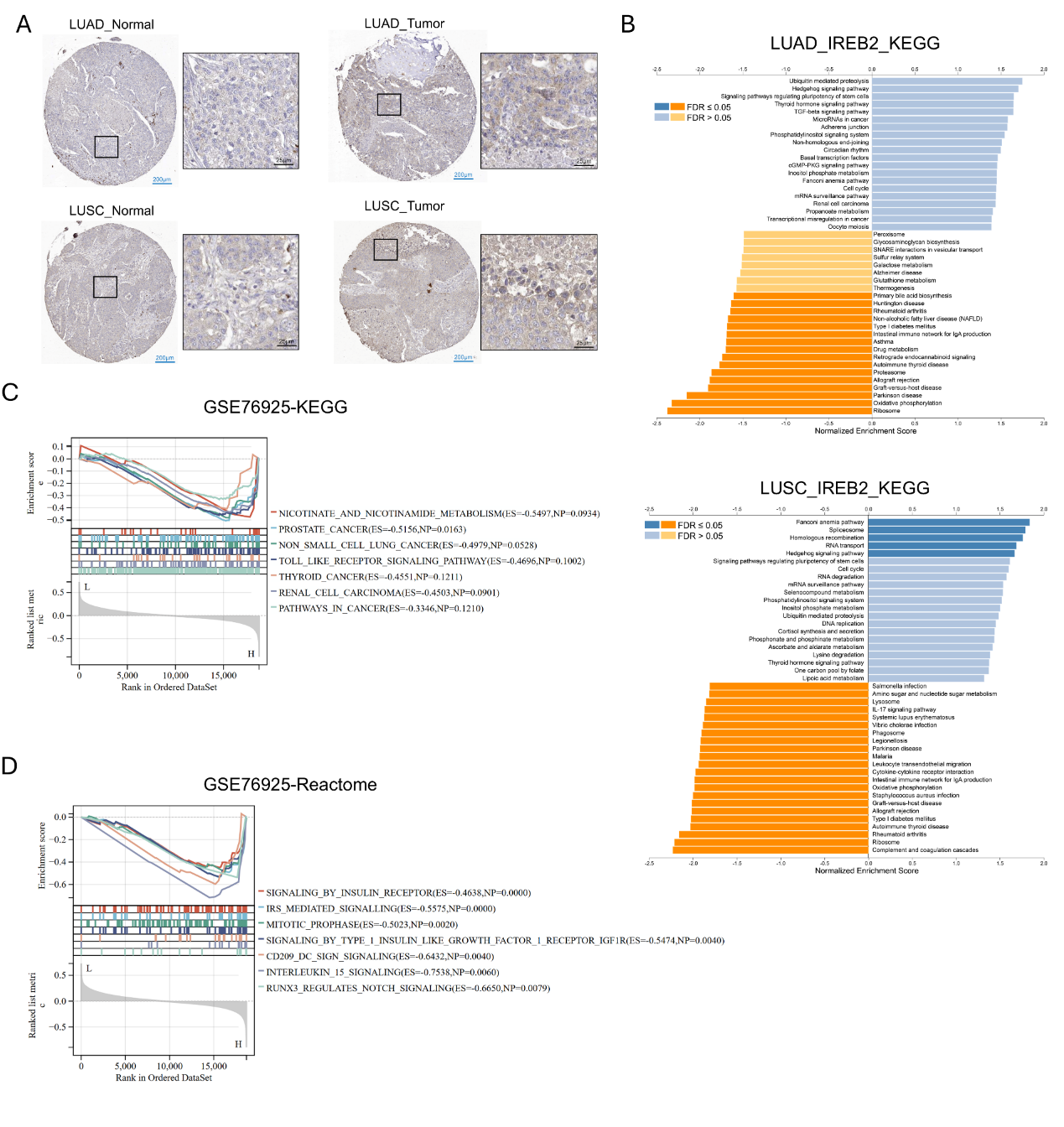


**Figure S8**

**A** Immunohistochemistry results of IREB2 in LUAD and LUSC, utilizing data from the HPA database.

**B** KEGG analysis results of IREB2 in LUAD and LUSC.

**C-D** KEGG (C) and Reactome (D) enrichment results of IREB2 in lung tissue sequencing from COPD patients using GSEA from GSE76925.


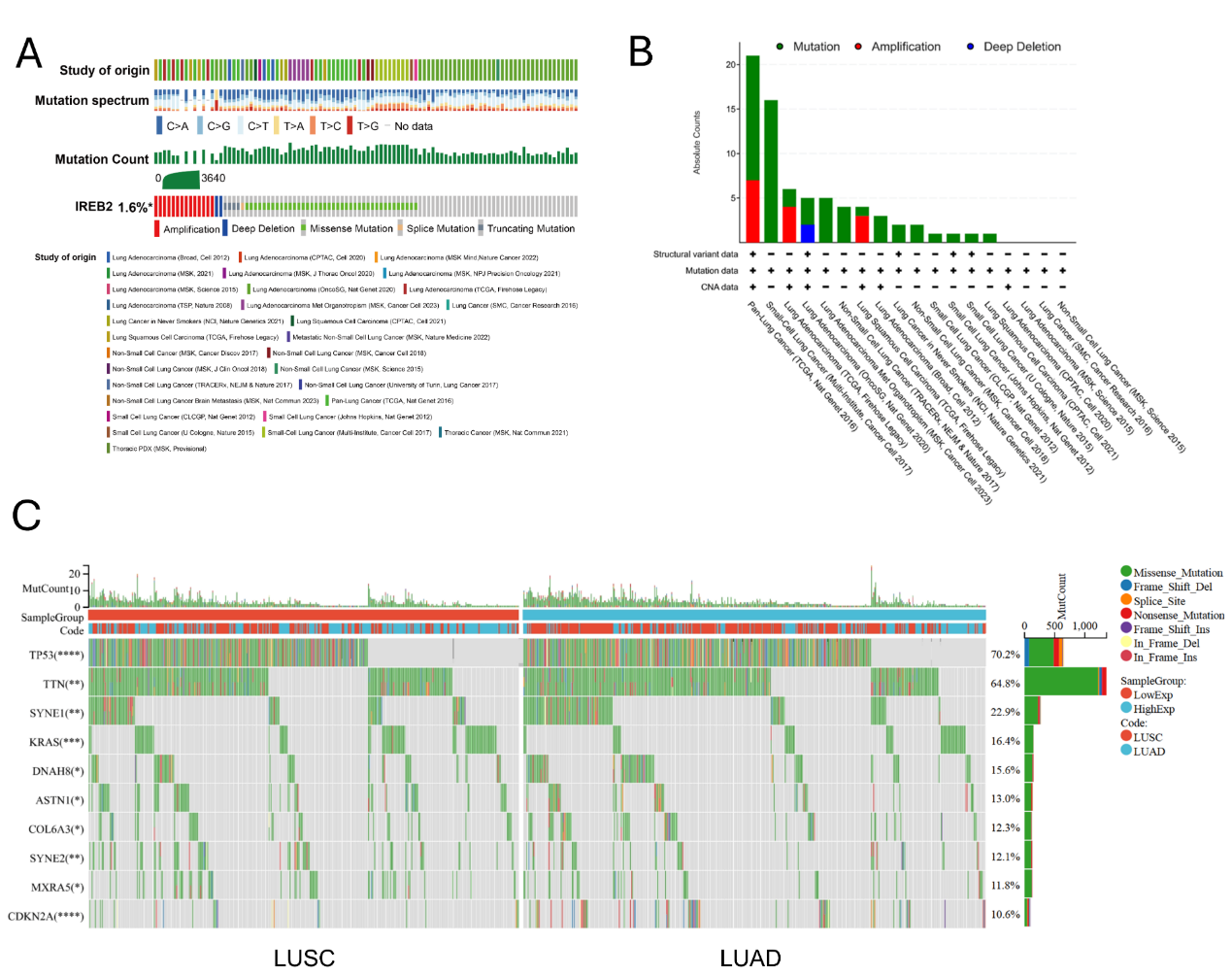


**Figure S9**

**A-B** The mutational plot for IREB2 in lung cancer, visualized with variant type, classification, and frequently mutated genes, using data from the cBioPortal database.

**C** Waterfall plot of the top 10 mutation genes associated with IREB2 in LUAD and LUSC. Two-tailed t-test, * (*P<0.05*), ** (*P<0.01*), *** (*P<0.001*), **** (*P<0.0001*).


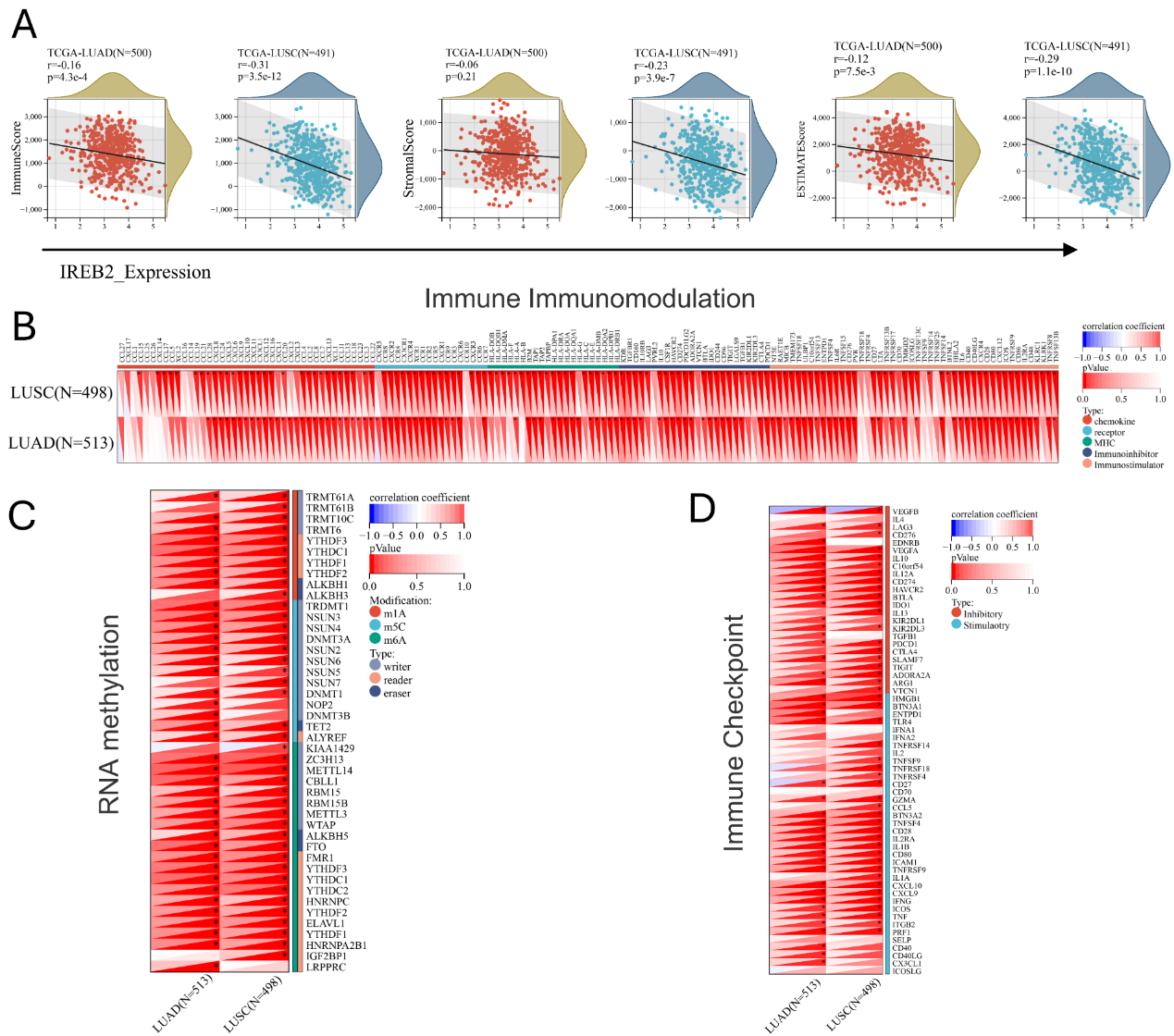


**Figure S10**

**A** Analysis of the relationship between IREB2 and immune infiltration in LUAD and LUSC using ImmunoScore, StromaScore, and ESTIMATEscore. Pearsons *correlation* *analysis*.

**B-D** Correlation of IREB2 with immune immunomodulation-related genes (B), RNA methylation-related genes (B) and Immune checkpoint-related genes (C) in LUAD and LUSC. Pearsons *correlation* *analysis.*


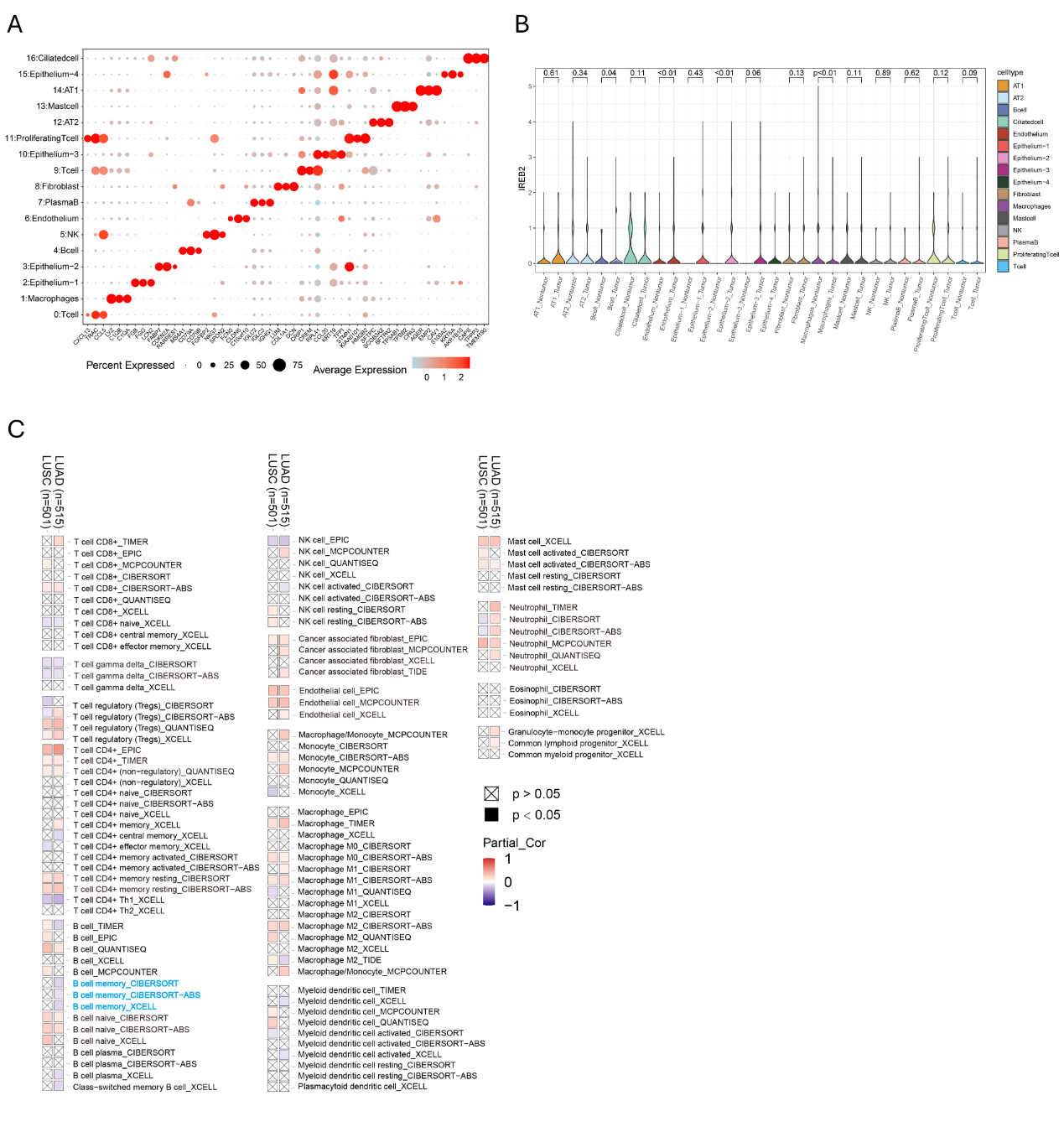


**Figure S11**

**A** Dotplot represented the top 3 marker genes for each cell subpopulation in the single-cell sequencing dataset.

**B** Violin plots visualize the distribution of IREB2 expression across cellular subclusters in tumor and adjacent normal tissues from lung cancer scRNA-seq data. Two-tailed t-test.

**C** Correlation analysis of IREB2 with the degree of immune cell infiltration in LUAD and LUSC using the TIMER2.0 database. Pearsons *correlation* *analysis*.


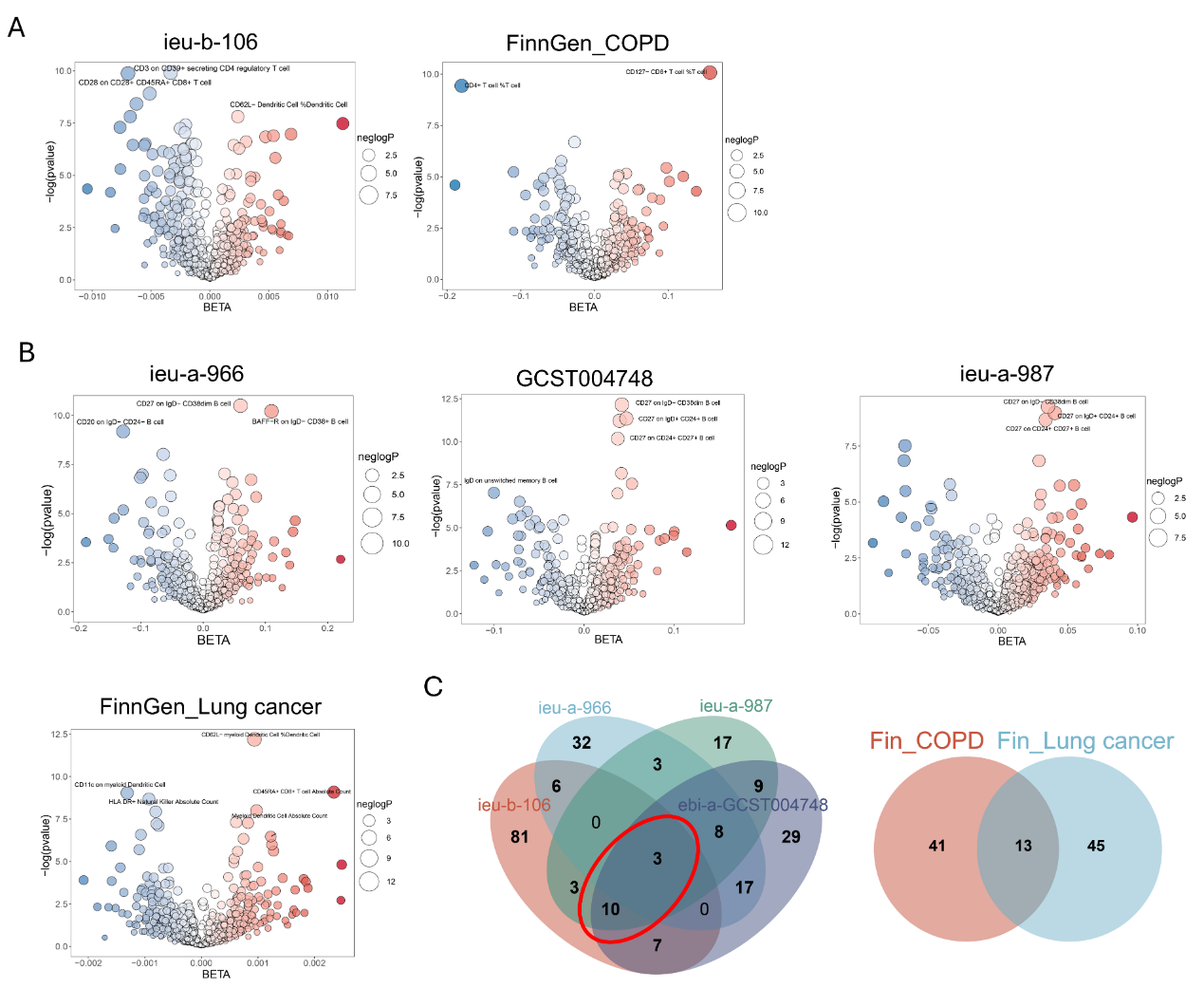


**Figure S12**

**A-B** Volcano plot showing the two-sample MR results for 731 immune cells in COPD (A) and lung cancer (B).

**C** Venn diagram illustrating the screening process for identifying shared immune cells in COPD and lung cancer based on two-sample Mendelian Randomization results for 731 immune cells.


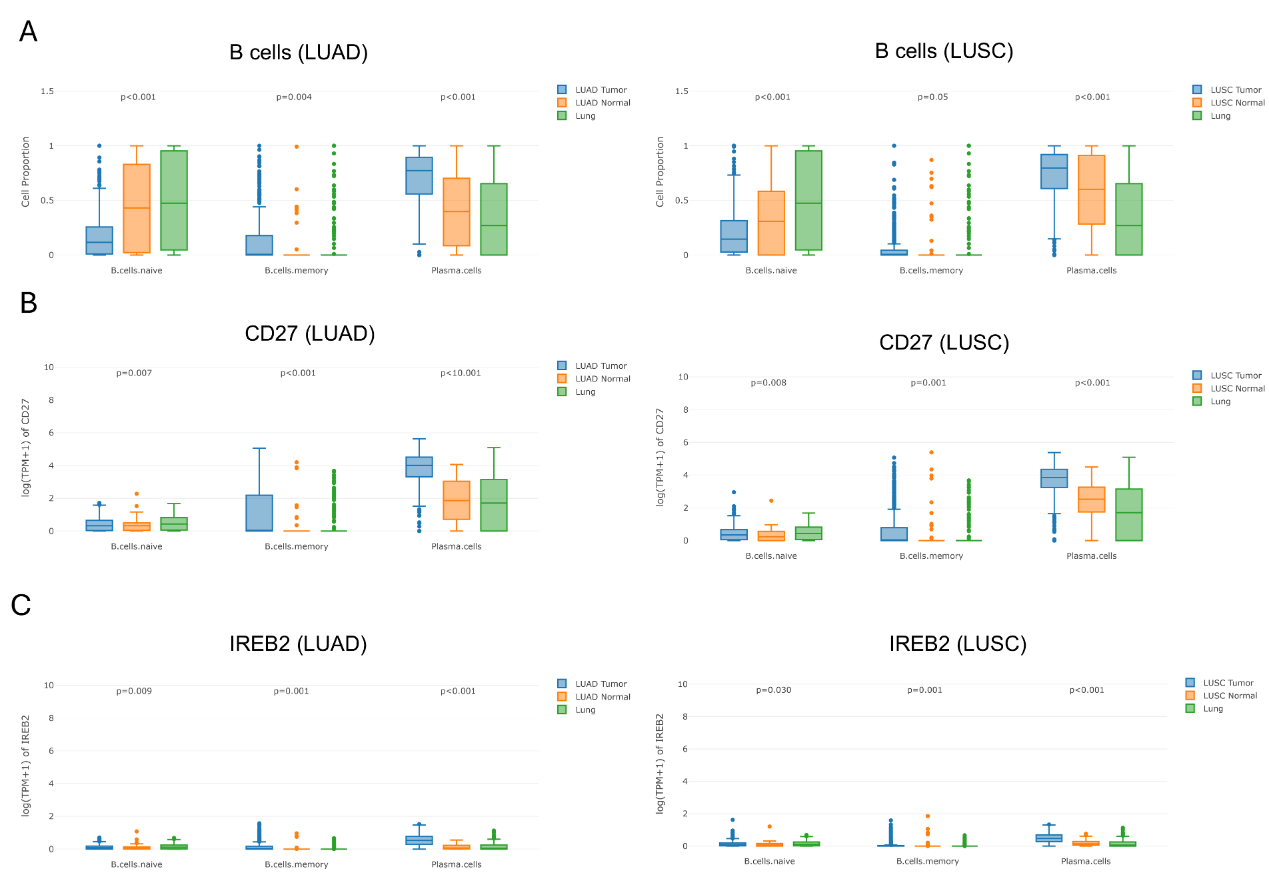


**Figure S13**

**A** Relative abundance of memory B cells, naive B cells and plasma cells in LUSC, LUAD and normal lung tissues, using data from the GEPIA database. *One-way ANOVA analysis*.

**B** Relative abundance of *IREB2* in memory B cells, naive B cells and plasma cells in LUSC, LUAD and normal lung tissues . *One-way ANOVA analysis*.

**C** Relative abundance of *PSMA4* in memory B cells, naive B cells and plasma cells in LUSC, LUAD and normal lung tissues . *One-way ANOVA analysis*.


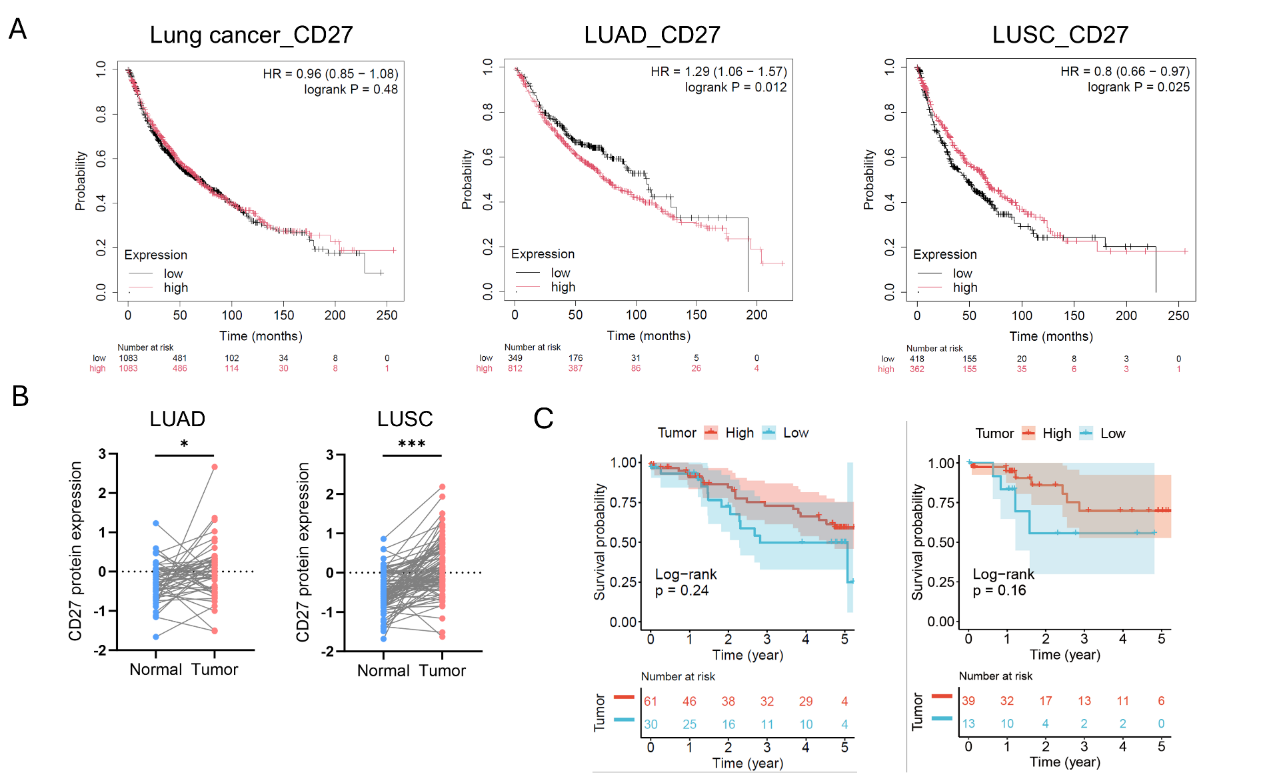


**Figure S14**

**A** Kaplan-Meier plots showing the prognostic significance of CD27 in lung cancer including LUAD and LUSC, using data from the KMplot database.

**B** Protein expression profiles of CD27 in tumor versus adjacent normal tissues from LUAD and LUSC samples derived from the CPTAC database. *Two-tailed t-test*, * (*P<0.05*), *** (*P<0.001*).

**C** Kaplan-Meier survival curves illustrating the prognostic value of CD27 protein expression levels in LUAD and LUSC cohorts, analyzed using CPTAC datasets.


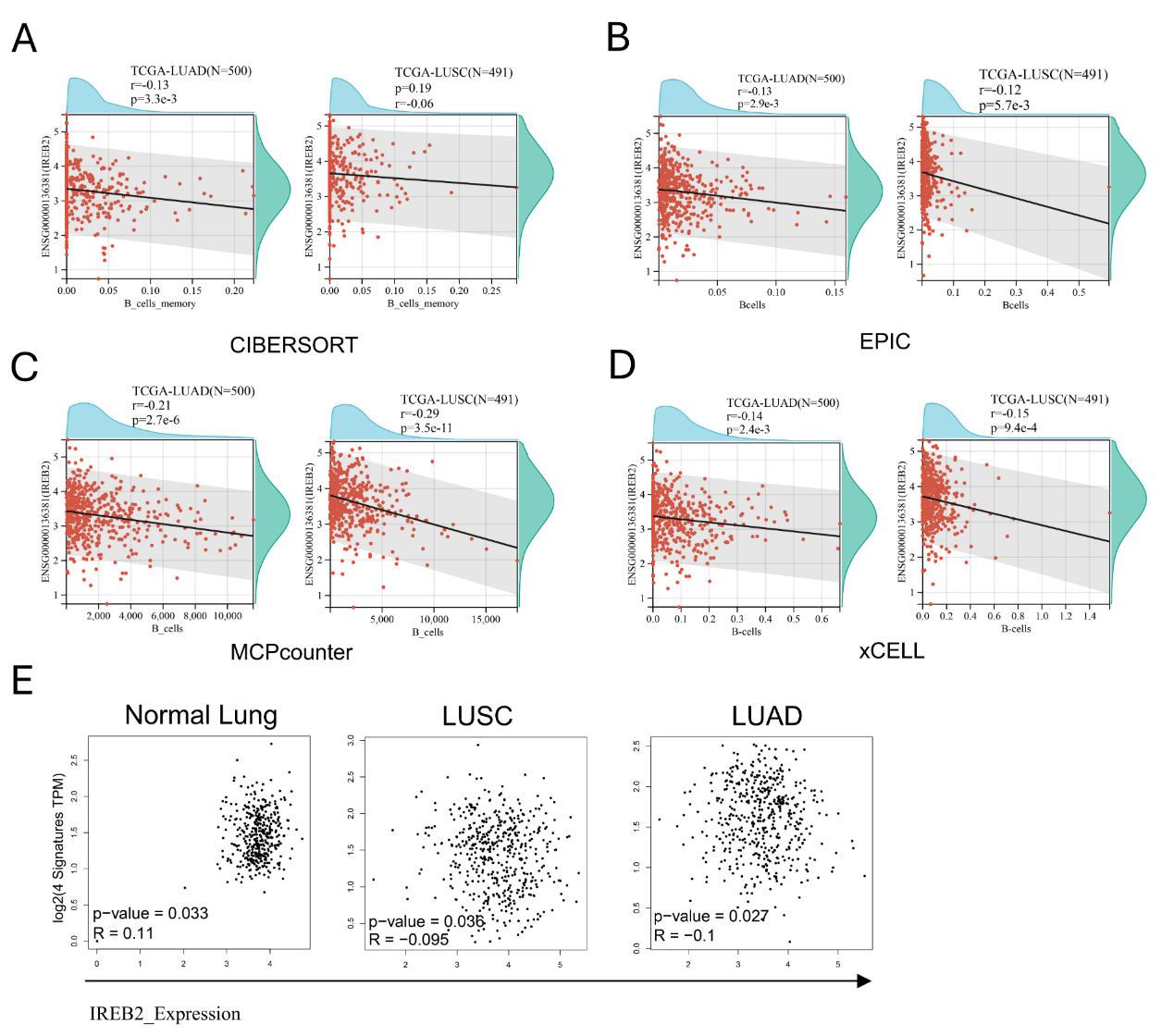


**Figure S15**

**A-D** Correlation analysis of *IREB2* with B cell immune infiltration in LUAD and LUSC using CIBERSORT (A), EPIC (B), MCPcounter (C) and xCELL (D) scoring. Pearsons *correlation* *analysis*.

**E** Correlation analysis of *IREB2* with memory B cell marker genes (CD19, MS4A1, CD27 and CD80) in LUAD and LUSC using the GEPIA2 database. Pearsons *correlation* *analysis*.


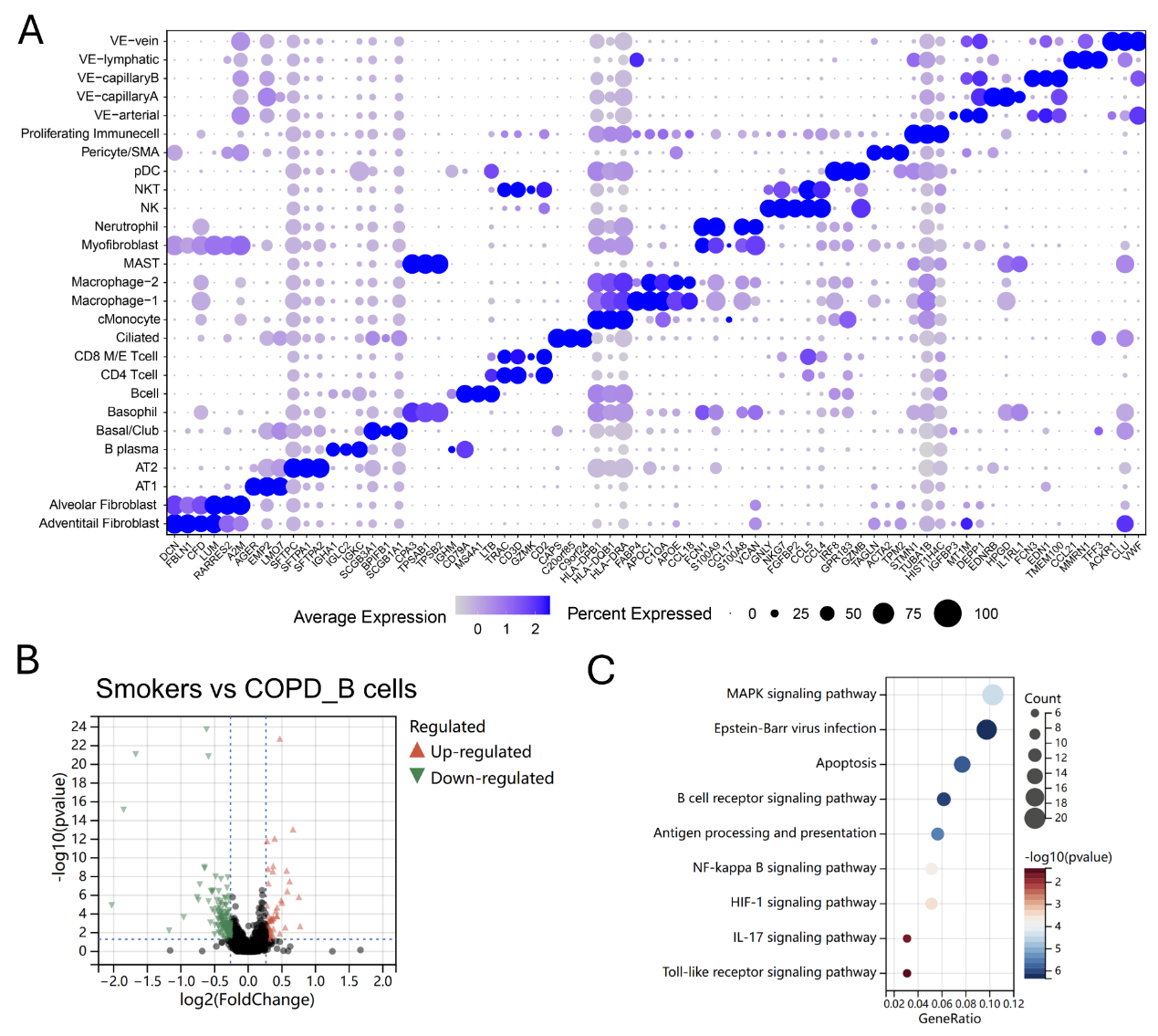


**Figure S16**

**A** UMAP plot illustrating the clustering of cell subpopulations in the single-cell dataset of COPD patients from GSE173896.

**B** Volcano plot showing the DEGs in B cells between COPD patients and smokers.

**C** KEGG enrichment results of DEGs in B cells between COPD patients and smokers.


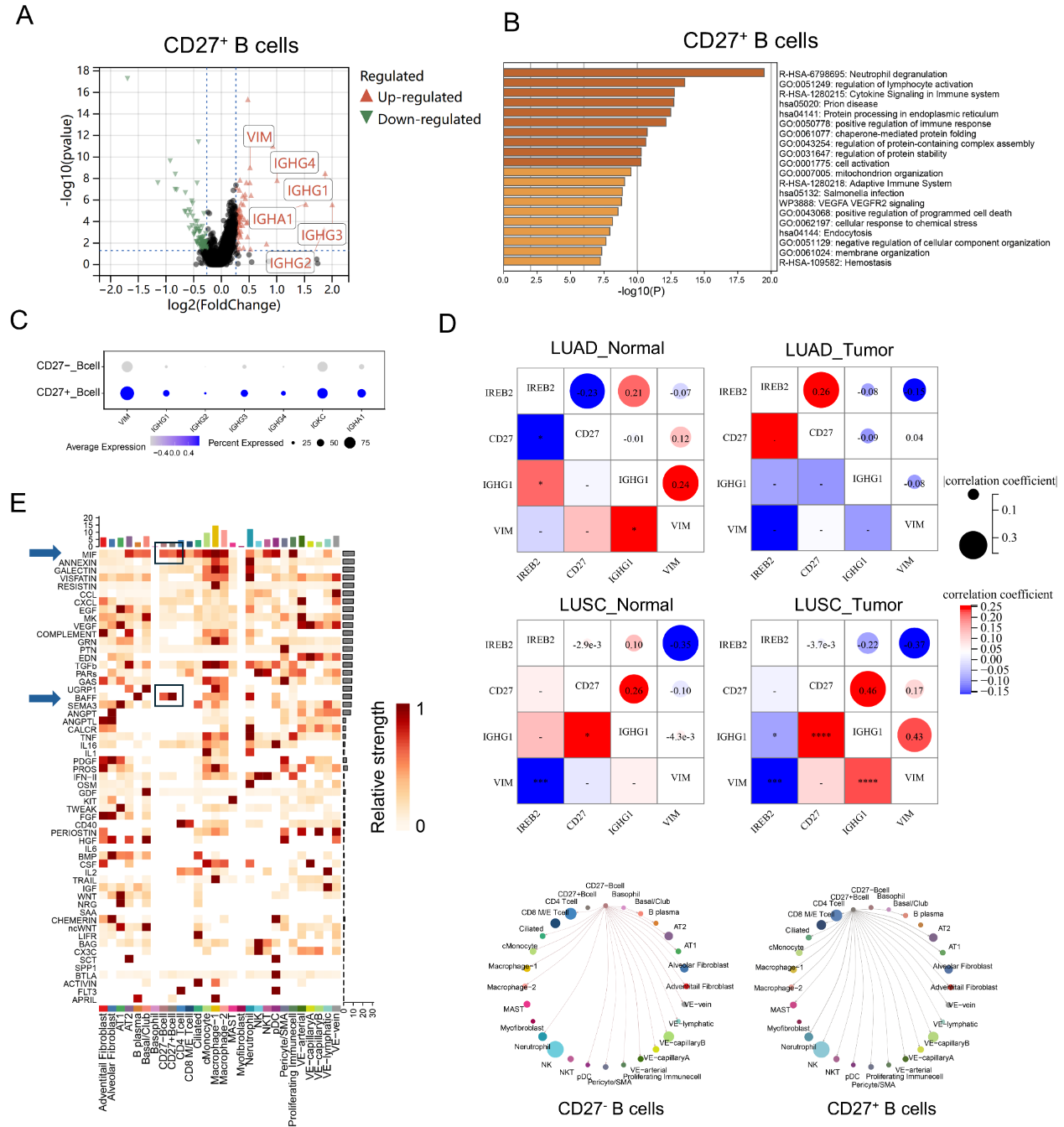


**Figure S17**

**A** Volcano plot showing the DEGs in B cells between COPD patients and smokers.

**B** Volcano plot showing the DEGs between CD27-positive and CD27-negative cells.

**C** Dotplot showing the expression of VIM, IGHG1, IGHG2, IGHG3, IGHG4, IGKC, and IGHA1 in CD27-positive and CD27-negative cells from GSE173896.

**D** Correlation analysis of protein expression levels of IREB2, CD27, IGHG1, and VIM in tumor and paired adjacent non-tumor tissues from LUAD and LUSC was performed using the CPCAT database. Pearson correlation analysis.

**E** Analysis of secretory-type intercellular interactions among various cell types within CD27+ and CD27- B cell clusters, including the degree of enrichment of incoming and outgoing signaling pathways, as identified in single-cell transcriptomes.

**F** Network graphs comparing pulmonary cell interactions in CD27- (top) and CD27+ (bottom) B cells as ligand cells. Each vertex represents a cellular subpopulation; edges signify ligand-receptor interactions. The thickness of the edges quantifies the cumulative expression of ligand-receptor genes, while the size of each vertex reflects Kleinberg centrality, indicating the cell's role in signaling. Cellular subpopulations are differentiated by color and number.


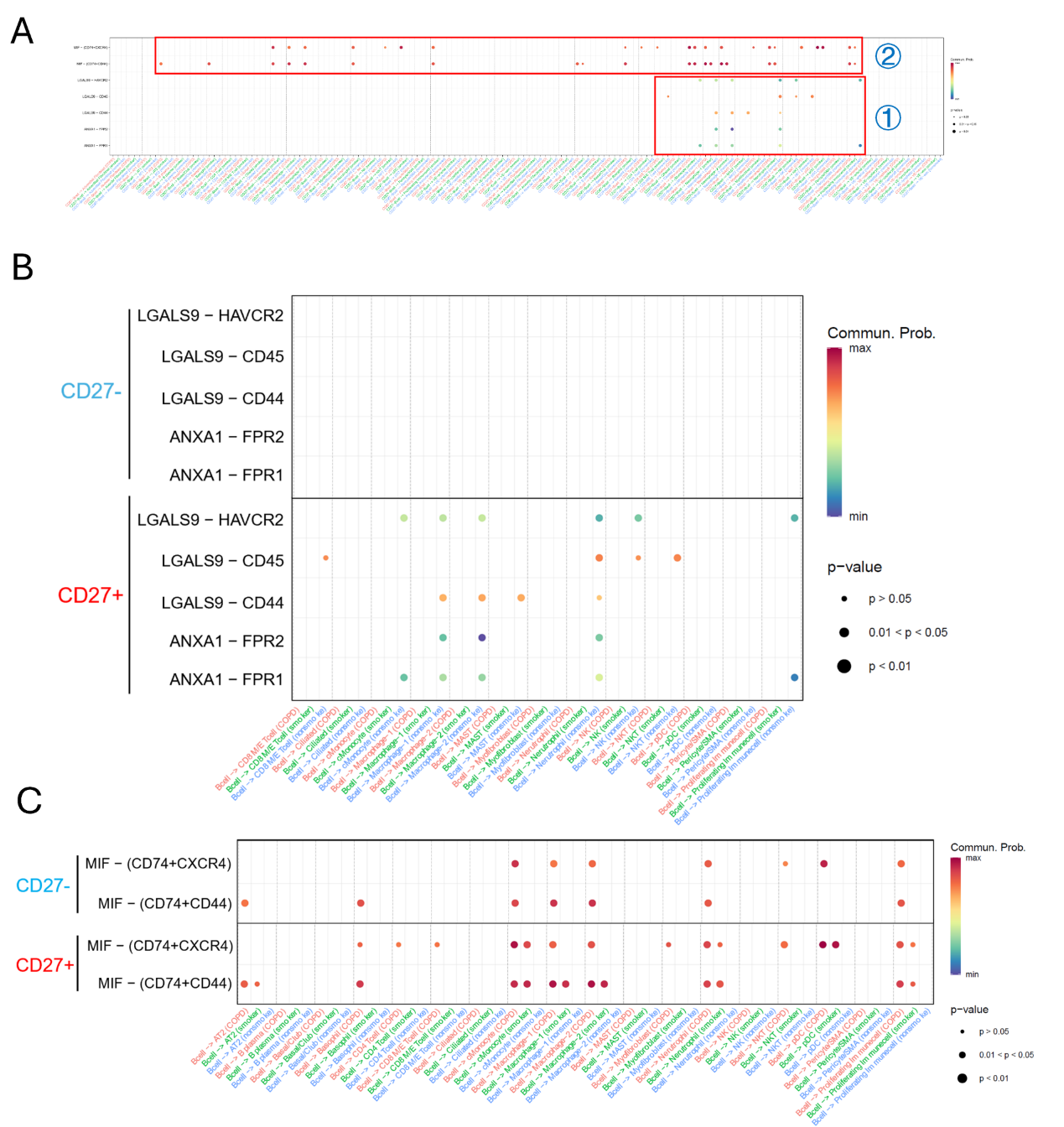


**Figure S18**

**A** Bubble chart visualizing cell-to-cell interactions involving CD27- or CD27+ B cells with other cell types sourced from GSE173896. B cells function as ligand-expressing cells in these interactions. ① and ② Significant ligand-receptor interactions between CD27- and CD27+ B cells.

**B-C** Bubble chart visualizes cell-to-cell interactions including LGALS9, ANXA1 (B) and CD74 (C) involving CD27- or CD27+ B cells with other cell types cells in nonsmokers, smokers and COPD, sourced from GSE173896. B cells function as ligand-expressing cells in these interactions.


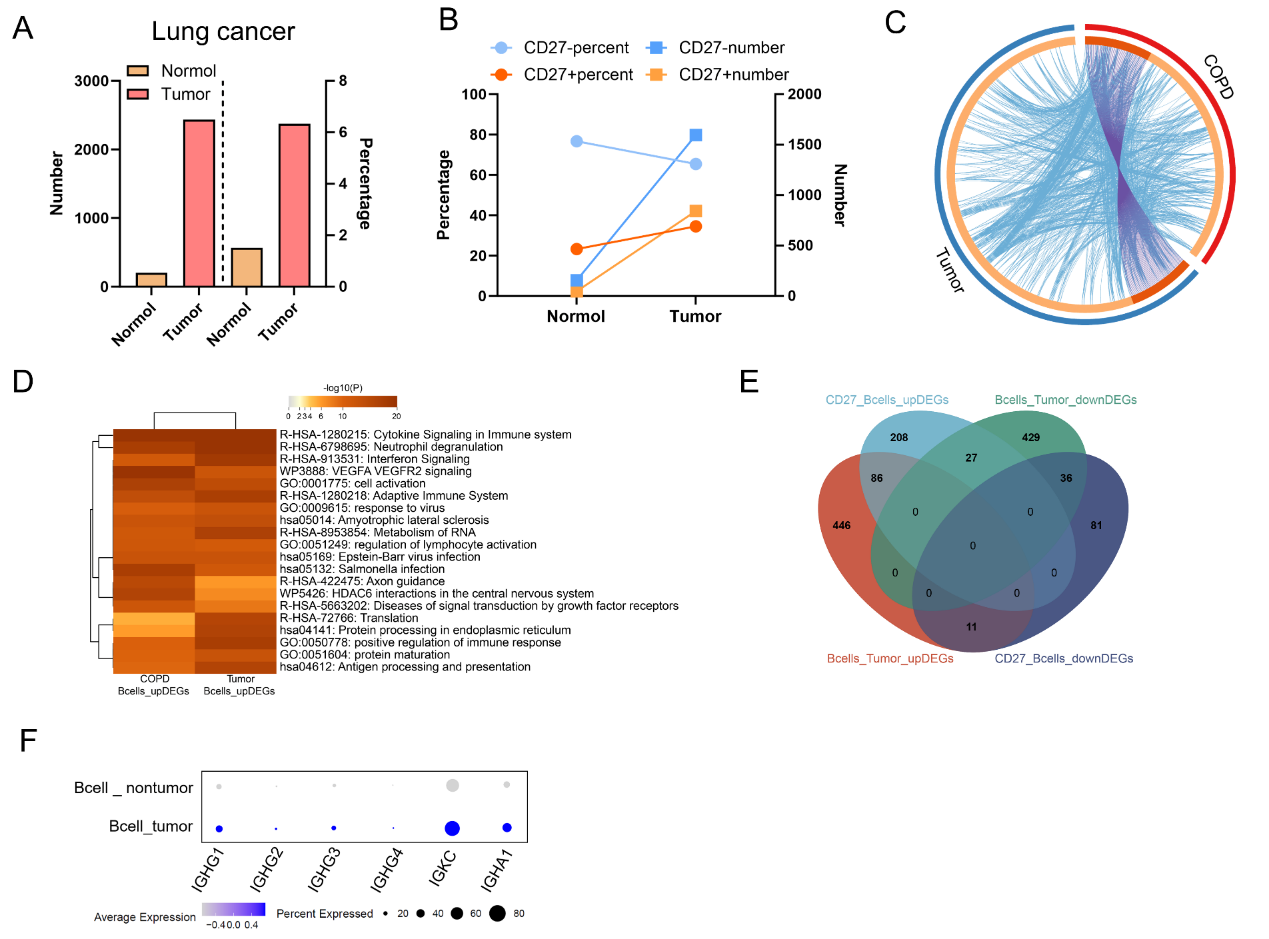


**Figure S19**

**A** Numbers and proportions of B cells in cancerous and adjacent normal tissues, derived from single-cell sequencing data of lung cancer.

**B** Numbers and proportions of CD27-positive and CD27-negative B cells in cancerous and adjacent normal tissues, derived from single-cell sequencing data of lung cancer.

**C** Overlap between gene lists of upregulated DEGs in B cells from COPD and lung cancer: purple curves link identical genes, and blue curves link genes that belong to the same enriched ontology term.

**D** Enrichment analysis results of upregulated DEGs in B cells from COPD and lung cancer, using Metascape.

**E** Average expression and percent expressed of IGHG1, IGHG2, IGHG3, IGHG4, IGKC, and IGHA1 in B cells from adjacent normal and cancerous tissues, derived from single-cell sequencing data of lung cancer.


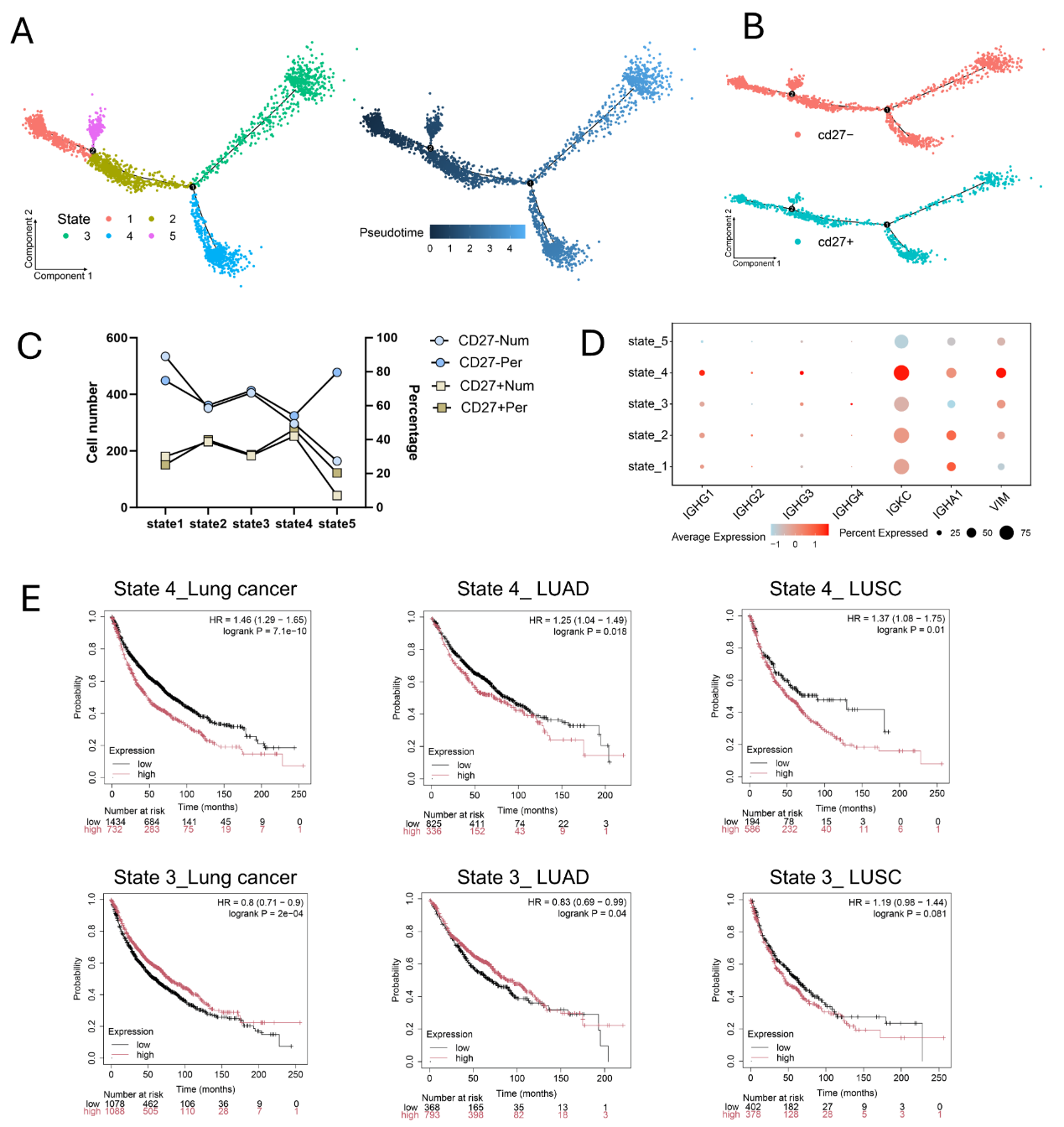


**Figure S20**

**A** Pseudotime trajectory analysis illustrates the developmental progression of B cells from single-cell sequencing data of lung cancer, color-coded by five distinct States.

**B-C** Pseudotime trajectory analysis delineates the developmental progression of CD27+and CD27- B cells (B) and the cell counts and relative proportions of CD27+and CD27- B cells within five distinct States of Pseudotime trajectory (C).

**D** Average expression and percent expressed of IGHG1-4, IGKC, VIM and IGHA1 in five distinct States of Pseudotime trajectory of B cells.

**E** Prognostic analysis of the top 10 markers of State_3 and State_4 B cells identified by Pseudotime trajectory in lung cancer, LUAD, and LUSC using KMplot.


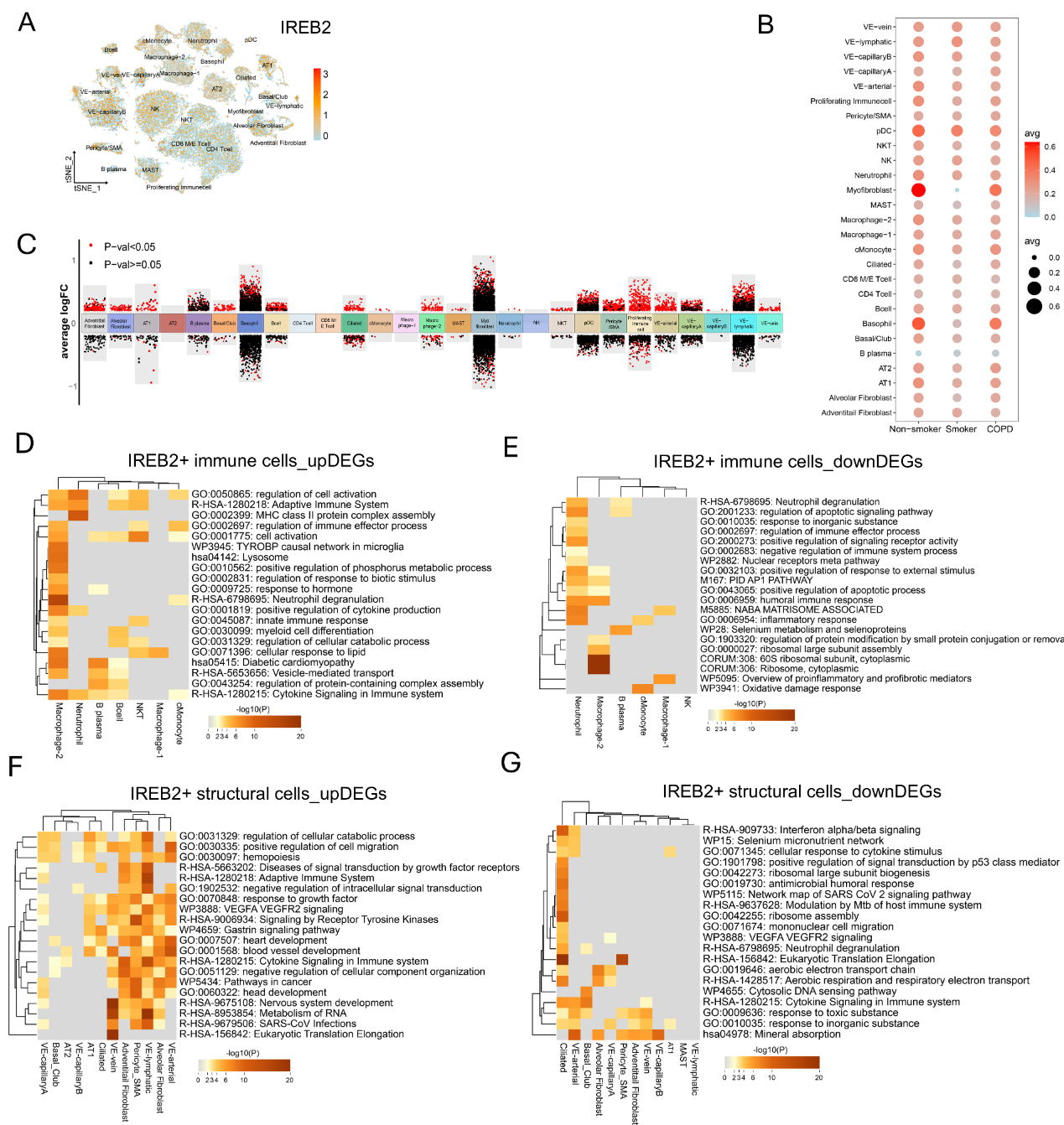


**Figure S21**

**A** UMAP plot showing the expression distribution of IREB2 across cell subpopulations from the GSE173896 dataset.

**B** Dotplot showing the differential expression of IREB2 across different cell subpopulations in nonsmokers, smokers, and COPD patients.

**C** Fold change plot of DEGs between IREB2-positive and IREB2-negative cells across various cell subpopulations.

**D-E** Enrichment analysis results of up-regulated DEGs in IREB2-positive and IREB2-negative pulmonary immune (D) and structural cells (E), using the Metascape database.

**F-G** Enrichment analysis results of down-regulated DEGs in IREB2-positive and IREB2-negative pulmonary immune (F) and structural cells (G), using the Metascape database.


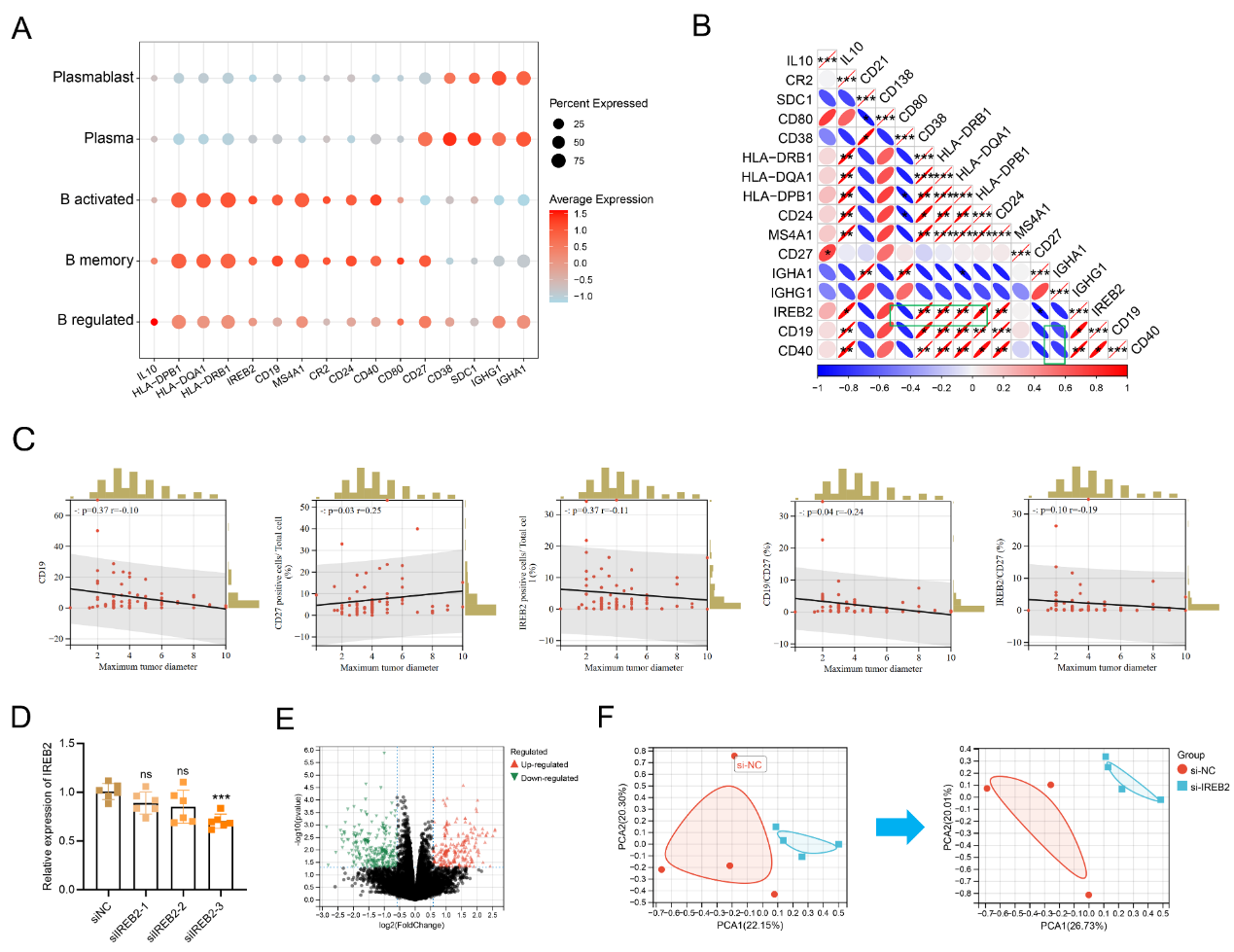


**Figure S22**

**A** Dot plot delineating marker gene expression profiles characteristic of distinct cellular subpopulations within integrated B cells.

**B** Pearson correlation analysis evaluating associations between IREB2 and B cell function/prognosis-related genes in integrated B cells.

**C** Pearson correlation statistics quantifying relationships of CD19⁺/CD27⁺/IREB2⁺ cell proportions and CD19/CD27, IREB2/CD27 ratios with maximum tumor diameter.

**D** qRT-PCR validation of IREB2 knockdown efficiency using three distinct siRNAs in SU-DHL-4 cells.

**E** Volcano plot exhibiting differentially expressed genes in si-IREB2 versus si-NC treated SU-DHL-4 cells from RNA sequencing.

**F** Principal component analysis (PCA) plot illustrating sample distribution of si-NC and si-IREB2 groups (left), with outlier-excluded clustering after quality control (right).


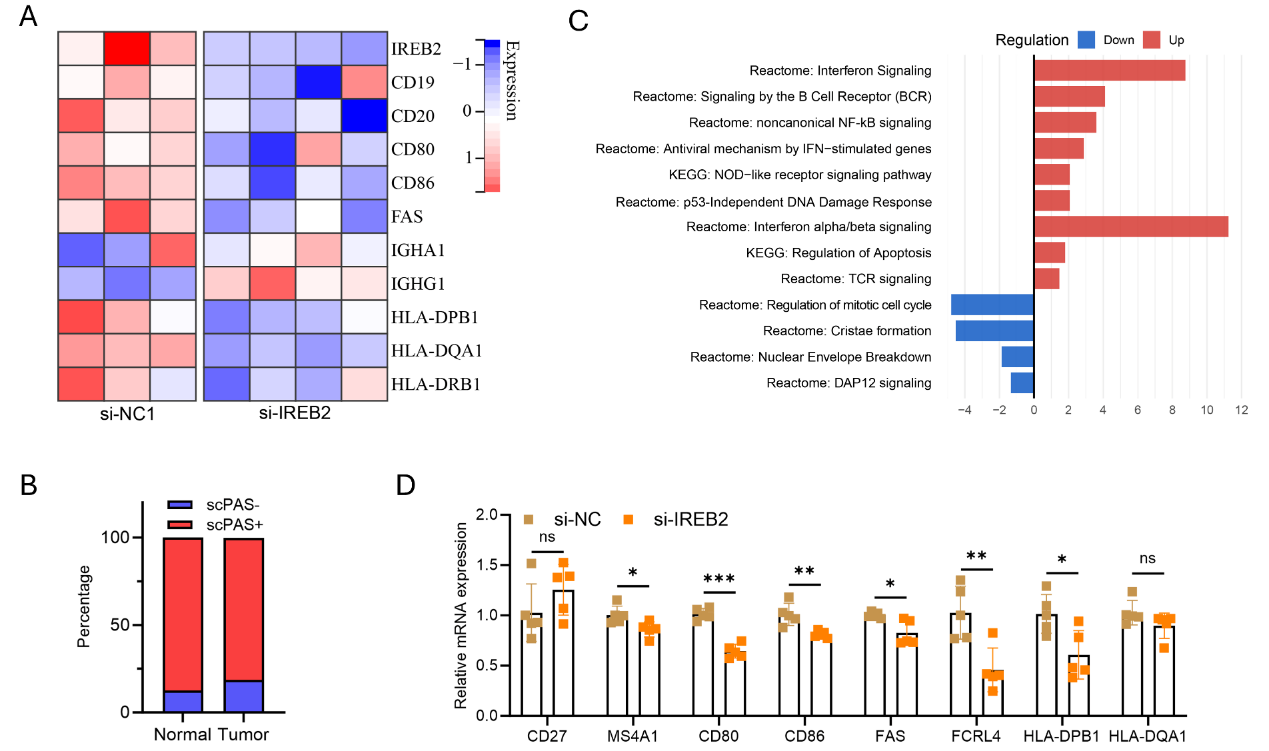


**Figure S23**

**A** Heatmap depicting the expression of selected genes in RNA-seq data from si-NC and si-IREB2 groups.

**B** Proportions of scPAS⁺ and scPAS⁻ B cells in adjacent non-tumor versus tumor tissues.

**C** KEGG and Reactome pathway enrichment analyses of genes differentially expressed between scPAS⁺ and scPAS⁻ cells.

**D** qRT-PCR assessment of mRNA levels of CD27, MS4A1, CD80, CD86, FAS, FCRL4, HLA-DPB1 and HLA-DQA1 in SU-DHL-4 cells following IREB2 knockdown (n = 5). Multiple t-tests.

**Supplementary Table 1. The weighted demographics and prevalence of COPD and lung cancer from 2013 to 2016 from NHANES**

| **Characteristics** | **Non-Lung cancer(N=7160)** | **Lung cancer(N=33)** | **Total(N=7193)** | **pvalue** |
| --- | --- | --- | --- | --- |
| **Age** |  |  |  | <0.001 |
| 20-39 | 3711(34.03%) | 1(0.009%) | 3712(34.04%) |  |
| 40-80 | 7160(65.66%) | 33(0.30%) | 7193(65.96%) |  |
| Mean±SD | 59.30±12.13 | 70.33±10.35 | 59.35±12.14 |  |
| **Sex** |  |  |  | 0.14 |
| Female | 5685(52.13%) | 13(0.12%) | 5698(52.25%) |  |
| Male | 5186(47.56%) | 21(0.19%) | 5207(47.75%) |  |
| **Race** |  |  |  | 0.05 |
| Mexican American | 1668(15.30%) | 1(0.009%) | 1669(15.30%) |  |
| Non-Hispanic Black | 2263(20.75%) | 7(0.06%) | 2270(20.82%) |  |
| Non-Hispanic Whtie | 4108(37.67%) | 20(0.18%) | 4128(37.85%) |  |
| Other Hispanic | 1204(11.04%) | 1(0.009%) | 1205(11.05%) |  |
| Other Race | 1628(14.93%) | 5(0.05%) | 1633(14.97%) |  |
| **BMI** |  |  |  | <0.001 |
| Mean±SD | 29.32±7.11 | 27.41±7.20 | 29.32±7.11 |  |
| **COPD** |  |  |  | <0.001 |
| COPD | 350(3.21%) | 11(0.10%) | 361(3.31%) |  |
| Non-COPD | 10521(96.48%) | 23(0.21%) | 10544(96.69%) |  |
| **Smoke status** |  |  |  | <0.001 |
| Ever Smoke | 4629(42.45%) | 29(0.27%) | 4658(42.71%) |  |
| Never Smoke | 6242(57.24%) | 5(0.05%) | 6247(57.29%) |  |

All *P* values are two-sided.

**Supplementary Table 2. The weighted demographics and prevalence of emphysema/chronic bronchitis and lung cancer from 1999 to 2016 from NHANES**

| **Characteristics** | **non-Lung Cancer(N=29687)** | **Lung Cancer(N=117)** | **Total(N=29804)** | **pvalue** |
| --- | --- | --- | --- | --- |
| **Age** |  |  |  | <0.001 |
| 20-39 | 16184(35.10%) | 4(0.008%) | 16188(35.11%) |  |
| 40-80 | 29803(64.63%) | 120(0.26%) | 29923(64.89%) |  |
| Mean±SD | 49.09±18.02 | 67.69±12.59 | 49.14±18.03 |  |
| **Gender** |  |  |  | 0.03 |
| Female | 23906(51.84%) | 52(0.11%) | 23958(51.96%) |  |
| Male | 22081(47.89%) | 72(0.16%) | 22153(48.04%) |  |
| **Race** |  |  |  | <0.001 |
| Mexican American | 8289(17.98%) | 1(0.002%) | 8290(17.98%) |  |
| Non-Hispanic Black | 9567(20.75%) | 28(0.06%) | 9595(20.81%) |  |
| Non-Hispanic Whtie | 20632(44.74%) | 82(0.18%) | 20714(44.92%) |  |
| Other Hispanic | 3736(8.10%) | 3(0.006%) | 3739(8.11%) |  |
| Other Race | 3763(8.16%) | 10(0.02%) | 3773(8.18%) |  |
| **BMI** |  |  |  | 0.02 |
| Mean±SD | 28.85±6.72 | 27.21±6.78 | 28.84±6.72 |  |
| **Emphysema** |  |  |  | <0.001 |
| Emphysema | 954(2.07%) | 30(0.07%) | 984(2.13%) |  |
| Non-Emphysema | 45033(97.66%) | 94(0.20%) | 45127(97.87%) |  |
| **Chronic Bronchitis** |  |  |  | <0.001 |
| Chronic bronchitis | 2701(5.86%) | 21(0.05%) | 2722(5.90%) |  |
| Non-Chronic bronchitis | 43286(93.87%) | 103(0.22%) | 43389(94.10%) |  |
| **Smoke Status** |  |  |  | <0.001 |
| Ever Smoke | 21052(45.66%) | 107(0.23%) | 21159(45.89%) |  |
| Never Smoke | 24935(54.08%) | 17(0.04%) | 24952(54.11%) |  |

All *P* values are two-sided.

**Supplementary Table 3. The weighted demographics, lung function, and prevalence of lung cancer from 2007 to 2012 from NHANES**

| **Characteristics** | **Non-Lung cancer(N=8291)** | **Lung cancer(N=14)** | **Total(N=8305)** | **pvalue** |
| --- | --- | --- | --- | --- |
| **Age** |  |  |  | 0.006 |
| 20-39 | 5279(39.65%) | 0(0.0e+0%) | 5279(39.65%) |  |
| 40-80 | 8020(60.24%) | 14(0.11%) | 8034(60.35%) |  |
| Mean±SD | 57.04±10.79 | 62.93±7.79 | 57.05±10.79 |  |
| **Gender** |  |  |  | 0.18 |
| Female | 6663(50.05%) | 10(0.08%) | 6673(50.12%) |  |
| Male | 6636(49.85%) | 4(0.03%) | 6640(49.88%) |  |
| **BMI** |  |  |  |  |
| Mean±SD | 29.07±6.81 | 26.78±6.38 | 29.06±6.81 |  |
| **Race** |  |  |  | 0.09 |
| Mexican American | 2107(15.83%) | 0(0%) | 2107(15.83%) |  |
| Non-Hispanic Black | 2903(21.81%) | 4(0.03%) | 2907(21.84%) |  |
| Non-Hispanic Whtie | 5680(42.67%) | 10(0.08%) | 5690(42.74%) |  |
| Other Hispanic | 1434(10.77%) | 0(0%) | 1434(10.77%) |  |
| Other Race | 1175(8.83%) | 0(0%) | 1175(8.83%) |  |
| **FEV_1_/FVC** |  |  |  | <0.001 |
| FEV_1_/FVC<0.7 | 2098(15.76%) | 12(0.09%) | 2110(15.85%) |  |
| FEV_1_/FVC=>0.7 | 11201(84.14%) | 2(0.02%) | 11203(84.15%) |  |
| Mean±SD | 76.05±8.40 | 58.09±12.92 | 76.02±8.44 |  |
| **FEV_1_** |  |  |  | <0.001 |
| Mean±SD | 3.04±0.90 | 1.64±0.56 | 3.04±0.90 |  |
| **FVC** |  |  |  | <0.001 |
| Mean±SD | 3.89±1.09 | 2.81±0.80 | 3.89±1.09 |  |
| **Smoke status** |  |  |  | 0.03 |
| Ever Smoke | 6007(45.12%) | 11(0.08%) | 6018(45.20%) |  |
| Never Smoke | 7292(54.77%) | 3(0.02%) | 7295(54.80%) |  |

All *P* values are two-sided.

**Supplementary Table 4. Weighted demographics and prevalence of lung cancer from 2013 to 2016 in NHANES after performing PSM analysis for COPD status**

| **Characteristics** | **COPD(N=350)** | **Non-COPD(N=350)** | **Total(N=700)** | **pvalue** |
| --- | --- | --- | --- | --- |
| **Age** |  |  |  | <0.001 |
| Mean±SD | 64.91±10.85 | 49.36±9.45 | 57.13±12.80 |  |
| **Gender** |  |  |  | <0.001 |
| Female | 161(23.00%) | 231(33.00%) | 392(56.00%) |  |
| Male | 189(27.00%) | 119(17.00%) | 308(44.00%) |  |
| **Race** |  |  |  | <0.001 |
| Mexican American | 19(2.71%) | 21(3.00%) | 40(5.71%) |  |
| Non-Hispanic Black | 58(8.29%) | 137(19.57%) | 195(27.86%) |  |
| Non-Hispanic White | 237(33.86%) | 129(18.43%) | 366(52.29%) |  |
| Other Hispanic | 15(2.14%) | 15(2.14%) | 30(4.29%) |  |
| Other Race - Including Multi-Racial | 21(3.00%) | 48(6.86%) | 69(9.86%) |  |
| **BMI** |  |  |  | 1 |
| Mean±SD | 30.46±8.30 | 30.28±9.19 | 30.37±9.59 |  |
| **Smokestatus** |  |  |  | 1 |
| Ever smoke | 304(43.43%) | 305(43.57%) | 609(87.00%) |  |
| Never smoke | 46(6.57%) | 45(6.43%) | 91(13.00%) |  |
| **Lungcancer** |  |  |  | <0.001 |
| Lung cancer | 11(1.57%) | 0(0%) | 11(1.57%) |  |
| Non-Lung cancer | 339(48.43%) | 350(50.00%) | 689(98.43%) |  |

All *P* values are two-sided.

**Supplementary Table 5. Weighted demographics and prevalence of lung cancer from 2013 to 2016 in NHANES after performing PSM analysis for Chronic bronchitis status**

| **Characteristics** | **Chronic bronchitis(N=2018)** | **Non-Chronic bronchitis(N=2017)** | **Total(N=29804)** | **pvalue** |
| --- | --- | --- | --- | --- |
| **LungCancer** |  |  |  | <0.001 |
| Lung cancer | 18(0.45%) | 2(0.05%) | 20 (0.50%) |  |
| Non-Lung cancer | 2000(49.57%) | 2015(49.94%) | 4015(99.50%) |  |
| **Age** |  |  |  | 0.06 |
| Mean±SD | 61.25±12.30 | 41.49±2.11 | 15239(51.13%) |  |
| **Gender** |  |  |  | <0.001 |
| Female | 1341(33.23%) | 1330(32.96%) | 4913(16.48%) |  |
| Male | 677(16.78%) | 687(17.03%) | 6209(20.83%) |  |
| **Race** |  |  | 14129(47.41%) |  |
| Mexican American | 190(4.71%) | 401(9.94%) | 2371(7.96%) |  |
| Non-Hispanic Black | 384(9.52%) | 529(13.11%) | 2182(7.32%) |  |
| Non-Hispanic White | 1211(30.01%) | 789(19.55%) |  |  |
| Other Hispanic | 128(3.17%) | 128(3.17%) | 29.17±6.52 |  |
| Other Race -Includin gMulti-Racial | 105(2.60%) | 170(4.21%) | 28.18[12.04,130.21] |  |
| **BMI** |  |  |  | <0.001 |
| Mean±SD | 30.83±8.21 | 31.67±8.64 | 895(3.00%) |  |
| **SmokeStatus** |  |  |  | <0.001 |
| Ever smoke | 1307(32.39%) | 1147(28.43%) | 14835(49.78%) |  |
| Never smoke | 711(17.62%) | 870(21.56%) | 14969(50.22%) |  |

All *P* values are two-sided.

**Supplementary Table 6. Weighted demographics and prevalence of lung cancer from 2013 to 2016 in NHANES after performing PSM analysis for Emphysema status**

| **Characteristics** | **Emphysema(N=895)** | **Non-Emphysema(N=895)** | **Total(N=1790)** | **pvalue** |
| --- | --- | --- | --- | --- |
| **Age** |  |  |  |  |
| Mean±SD | 67.14±11.19 | 53.55±12.60 | 60.34±13.72 |  |
| **Gender** |  |  |  | 0.05 |
| Female | 367(20.50%) | 325(18.16%) | 692(38.66%) |  |
| Male | 528(29.50%) | 570(31.84%) | 1098(61.34%) |  |
| **Race** |  |  |  | <0.001 |
| Mexican American | 50(2.79%) | 66(3.69%) | 116(6.48%) |  |
| Non-Hispanic Black | 120(6.70%) | 166(9.27%) | 286(15.98%) |  |
| Non-Hispanic White | 623(34.80%) | 123(6.87%) | 746(41.68%) |  |
| Other Hispanic | 51(2.85%) | 11(0.61%) | 62(3.46%) |  |
| Other Race - Including Multi-Racial | 51(2.85%) | 529(29.55%) | 580(32.40%) |  |
| **BMI** |  |  |  |  |
| Mean±SD | 28.27±6.90 | 25.56±5.70 | 26.92±6.47 |  |
| **SmokeStatus** |  |  |  | 0.42 |
| Ever smoke | 816(45.59%) | 805(44.97%) | 1621(90.56%) |  |
| Never smoke | 79(4.41%) | 90(5.03%) | 169(9.44%) |  |
| **LungCancer** |  |  |  | <0.001 |
| Lung cancer | 28(1.56%) | 5(0.28%) | 33(1.84%) |  |
| Non-Lung cancer | 867(48.44%) | 890(49.72%) | 1757(98.16%) |  |

All *P* values are two-sided.

**Supplementary Table 7. Weighted demographics and prevalence of lung cancer from 2013 to 2016 in NHANES after performing PSM analysis for COPD diagnosed by lung function**

| **Characteristics** | **COPD (N=1593)** | **Non-COPD (N=1591)** | **Total(N=3184)** | **pvalue** |
| --- | --- | --- | --- | --- |
| **Age** |  |  |  | 0.0015 |
| Mean±SD | 61.35±10.69 | 52.32±11.47 | 56.84±11.97 |  |
| **Gender** |  |  |  | <0.001 |
| Female | 627(19.69%) | 412(12.94%) | 1039(32.63%) |  |
| Male | 966(30.34%) | 1179(37.03%) | 2145(67.37%) |  |
| **Race** |  |  |  | <0.001 |
| Mexican American | 128(4.02%) | 187(5.87%) | 315(9.89%) |  |
| Non-Hispanic Black | 314(9.86%) | 396(12.44%) | 710(22.30%) |  |
| Non-Hispanic White | 950(29.84%) | 671(21.07%) | 1621(50.91%) |  |
| Other Hispanic | 111(3.49%) | 152(4.77%) | 263(8.26%) |  |
| Other Race - Including Multi-Racial | 90(2.83%) | 185(5.81%) | 275(8.64%) |  |
| **BMI** |  |  |  |  |
| Mean±SD | 27.85±6.06 | 26.32±4.76 | 27.08±5.50 |  |
| **Smokestatus** |  |  |  | <0.001 |
| Ever smoke | 1121(35.21%) | 1406(44.16%) | 2527(79.37%) |  |
| Never smoke | 472(14.82%) | 185(5.81%) | 657(20.63%) |  |
| **FEV_1_** |  |  |  | <0.001 |
| Mean±SD | 2.36±0.78 | 3.18±0.76 | 2.77±0.87 |  |
| **FVC** |  |  |  | <0.001 |
| Mean±SD | 3.71±1.11 | 4.07±0.97 | 3.89±1.05 |  |

All *P* values are two-sided.

**Supplementary Table 8. Odds Ratio (OR) analysis of COPD, emphysema, chronic bronchitis, lung function, and lung cancer**

| **Independent variables** | **Model 1^1^** | **Model 2^2^** | **Model 3^3^** |
| --- | --- | --- | --- |
| COPD | 14.37(6.95-29.72) ^***^ | 14.37(6.95-29.72) ^***^ | 23.74(1.39- 404.55)^*^ |
| FEV_1_/FVC<0.7 | 32.03(7.16-143.23) ^***^ | 32.03(7.16-143.23) ^***^ | 25.15(1.48 to 425.29)^*^ |
| Emphysema | 15.28(10.02-23.29) ^***^ | 15.28(10.02-23.29) ^***^ | 5.74(2.20 to 14.95)^**^ |
| Chronic bronchitis | 3.05(1.87-4.99) ^***^ | 3.05(1.87-4.99) ^***^ | 9.06(2.10 to 39.13)^*^ |

^1^Model 1: Age20-80

^2^Model 2: Age40-80

^3^Model 3: Age40-80 after PSM analysis

Abbreviation: OR: Odds ratio

**P<0.05, ***P<0.001*. All *P* values are two-sided.

**Supplementary Table 9.** **Demographic and clinical baseline characteristics of peripheral blood specimens in the ECOPD cohort**

| **Characteristics** | **COPD (N=55)** | **Non-COPD (N=49)** | **Total(N=104)** | **pvalue** |
| --- | --- | --- | --- | --- |
| **Age** |  |  |  | <0.001 |
| Mean±SD | 65.67±6.48 | 52.32±11.47 | 62.78±7.72 |  |
| **Gender** |  |  |  | <0.001 |
| Female | 1(0.96%) | 28(26.92%) | 29(27.88%) |  |
| Male | 54(51.92%) | 21(20.19%) | 75(72.12%) |  |
| **BMI** |  |  |  |  |
| Mean±SD | 21.86±3.50 | 23.83±3.27 | 22.79±3.52 |  |
| **Smokestatus** |  |  |  | <0.001 |
| Never smoke | 2(1.92%) | 34(32.69%) | 36(34.62%) |  |
| Ever Smoke | 31(29.81%) | 3(2.88%) | 34(32.69%) |  |
| Current smoke | 22(21.15%) | 12(11.54%) | 34(32.69%) |  |
| **Smoke index** |  |  |  |  |
| Mean±SD | 38.12±29.49 | 7.98±13.82 | 23.92±27.81 |  |
| **pre_FEV_1_** **(**Mean±SD) | 1.71±0.55 | 2.23±0.61 | 1.95±0.63 | <0.001 |
| **pre_FVC (**Mean±SD) | 3.30±0.67 | 2.93±0.84 | 3.12±0.77 | <0.001 |
| **pre_FEV_1_/FVC% (**Mean±SD) | 51.31±10.21 | 76.51±6.60 | 63.18±15.32 | <0.001 |
| **post_FEV_1_ (**Mean±SD) | 1.84±0.57 | 2.33±0.62 | 2.07±0.64 | <0.001 |
| **post_FVC (**Mean±SD) | 3.43±0.68 | 2.96±0.82 | 3.21±0.78 | <0.001 |
| **post_FEV_1_/FVC% (**Mean±SD) | 53.22±10.42 | 79.12±5.95 | 65.42±15.56 | <0.001 |

Abbreviation: pre_FEV_1_: ​pre-bronchodilator Forced Expiratory Volume in 1 second​; post_FEV_1_: post-bronchodilator Forced Expiratory Volume in 1 second​.

All *P* values are two-sided.

**Supplementary Table 10. Multiple linear regression analysis​ of peripheral blood IREB2 and PSMA4 expression ​versus​ lung function decline rate ​in the ECOPD cohort**

| **Variable** | **IREB2** | | **PSMA4** | |
| --- | --- | --- | --- | --- |
|  | OR (95% CI) | P Value | OR (95% CI) | P value |
| pre-FEV_1_ annual decline **^a^** | 67.0 (15.0 to 119.0) | **0.013** | 51.6 (-25.6 to 128.8) | 0.194 |
| pre-FVC annual decline **^a^** | 114 (23.4 to 204.6) | **0.015** | 144 (24.7 to 264.8) | **0.020** |
| pre- FEV_1_/FVC annual decline **^a^** | -0.8 (-2.3 to 0.7) | 0.303 | -0.85 (-3.2 to 1.5) | 0.475 |
| post-FEV_1_ annual decline **^a^** | 56.1 (2.0 to 110.3) | **0.045** | 0.12 (-79.9 to 80.2) | 0.997 |
| post-FVC annual decline **^a^** | 59.1 (-22.8 to 141.2) | 0.161 | 79.5 (-29.2 to 188.1) | 0.155 |
| post- FEV_1_/FVC annual decline **^a^** | -0.9 (-2.5 to 0.6) | 0.227 | -1.2 (-3.6 to 1.2) | 0.333 |

Abbreviation: pre_FEV_1_: ​pre-bronchodilator Forced Expiratory Volume in 1 second​; post_FEV_1_: post-bronchodilator Forced Expiratory Volume in 1 second​.

**^a^**Adjusted for age, sex, smoking status, smoke index, BMI and Baseline lung function

All *P* values are two-sided.

**Supplementary Table 11. Demographic and clinical baseline characteristics of peripheral blood specimens in the ECOPD cohort**

| **Characteristics** | **LUSC(N=37)** | **LUAD(N=37)** | **Total(N=74)** | **pvalue** |
| --- | --- | --- | --- | --- |
| **Sex** |  |  |  | 0.001 |
| Female | 5(6.76%) | 19(25.68%) | 24(32.43%) |  |
| Male | 32(43.24%) | 18(24.32%) | 50(67.57%) |  |
| **Age** |  |  |  |  |
| Mean±SD | 61.89±7.32 | 57.41±7.04 | 59.65±7.48 |  |
| **Degree of differentiation** |  |  |  | 0.35 |
| Well | 5(6.76%) | 10(13.51%) | 15(20.27%) |  |
| Moderately | 19(25.68%) | 15(20.27%) | 34(45.95%) |  |
| Moderately-poorly | 0(0.0e+0%) | 1(1.35%) | 1(1.35%) |  |
| Poorly | 13(17.57%) | 11(14.86%) | 24(32.43%) |  |
| **Maximum tumor diameter** |  |  |  |  |
| Mean±SD | 4.51±2.20 | 3.82±1.87 | 4.16±2.06 |  |
| **Survival state** |  |  |  | 0.54 |
| death | 12(16.22%) | 8(10.81%) | 20(27.03%) |  |
| live | 16(21.62%) | 20(27.03%) | 36(48.65%) |  |
| unknow | 9(12.16%) | 9(12.16%) | 18(24.32%) |  |

Abbreviation: LUAD: lung adenocarcinomas; LUSC: lung squamous cell carcinomas.

All *P* values are two-sided.

**Supplementary Table 12.** **Primers used in experiments.**

| **Primer name** | **Forward primer (5’-3’)** | **Reverse primer (5’-3’)** | **Application** |
| --- | --- | --- | --- |
| GAPDH | CAGCCTCAAGATCATCAGCA | ACAGTCTTCTGGGTGGCAGT | qRT-PCR |
| IREB2 | GACGCCCCAAAAGCAGGATA | AAGAACACGGGCAGGGAAAA | qRT-PCR |
| PSMA4 | TCCGTGGACATCTCAGGTCT | TCTGCTGCAAGCAAAACACC | qRT-PCR |
| CD80 | GGGAAATGTCGCCTCTCTGAA | CAAAACAGGCAGGGCTGATG | qRT-PCR |
| CD27 | GTGCTAACTCCAGAGGCCAG | AAGGATCACACTGAGCAGCC | qRT-PCR |
| CD86 | TGATGGCCTTCCTGCTCTCT | CAGGGTCCAACTGTCCGAAT | qRT-PCR |
| FCRL4 | AGGTGGAGCTTCAGTCGTTG | CCTGGGACTTTGGACAAGGG | qRT-PCR |
| FAS | GGACCCTCCTACCTCTGGTT | CTCCTTCCCTTCTTGGCAGG | qRT-PCR |
|  |  |  |  |
